# Spinocerebellar ataxia 38: structure–function analysis shows ELOVL5 G230V is proteotoxic, conformationally altered and a mutational hotspot

**DOI:** 10.1007/s00439-023-02572-y

**Published:** 2023-05-18

**Authors:** Enza Ferrero, Eleonora Di Gregorio, Marta Ferrero, Erika Ortolan, Young-Ah Moon, Antonella Di Campli, Lisa Pavinato, Cecilia Mancini, Debasmita Tripathy, Marta Manes, Eriola Hoxha, Chiara Costanzi, Elisa Pozzi, Matteo Rossi Sebastiano, Nico Mitro, Filippo Tempia, Donatella Caruso, Barbara Borroni, Manuela Basso, Michele Sallese, Alfredo Brusco

**Affiliations:** 1 Department of Medical Sciences, University of Torino, Via Santena 19, 10126 Turin, Italy; 2 Unit of Medical Genetics, Città della Salute e Della Scienza Hospital, Turin, Italy; 3 Experimental Zooprophylactic Institute of Piedmont, Liguria and Aosta Valley, Turin, Italy; 4 Department of Molecular Medicine, Inha University College of Medicine, Incheon, South Korea; 5 Institute of Protein Biochemistry, Italian National Research Council, Naples, Italy; 6 Department of Innovative Technologies in Medicine and Dentistry, G. d’Annunzio University of Chieti-Pescara, Chieti, Italy; 7 Genetics and Rare Diseases Research Division, Bambino Gesù Children’s Hospital, Rome, Italy; 8 Department of Cellular, Computational and Integrative Biology, University of Trento, Trento, Italy; 9 Department of Clinical and Experimental Sciences, University of Brescia, Brescia, Italy; 10 Neuroscience Institute Cavalieri Ottolenghi, Orbassano and Department of Neuroscience, University of Torino, Turin, Italy; 11 Department of Neurology, Cremona Hospital, Cremona, Italy; 12 Department of Molecular Biotechnology and Health Sciences, University of Torino, Turin, Italy; 13 Department of Pharmacological and Biomolecular Sciences, University of Milan, Milan, Italy; 14 Centre for Advanced Studies and Technology, G. d’Annunzio University of Chieti-Pescara, Chieti, Italy

## Abstract

Fatty acid elongase ELOVL5 is part of a protein family of multipass transmembrane proteins that reside in the endoplasmic reticulum where they regulate long-chain fatty acid elongation. A missense variant (c.689G>T p.Gly230Val) in *ELOVL5* causes Spinocerebellar Ataxia subtype 38 (SCA38), a neurodegenerative disorder characterized by autosomal dominant inheritance, cerebellar Purkinje cell demise and adult-onset ataxia. Having previously showed aberrant accumulation of p.G230V in the Golgi complex, here we further investigated the pathogenic mechanisms triggered by p.G230V, integrating functional studies with bioinformatic analyses of protein sequence and structure. Biochemical analysis showed that p.G230V enzymatic activity was normal. In contrast, SCA38-derived fibroblasts showed reduced expression of ELOVL5, Golgi complex enlargement and increased proteasomal degradation with respect to controls. By heterologous overexpression, p.G230V was significantly more active than wild-type ELOVL5 in triggering the unfolded protein response and in decreasing viability in mouse cortical neurons. By homology modelling, we generated native and p.G230V protein structures whose superposition revealed a shift in Loop 6 in p.G230V that altered a highly conserved intramolecular disulphide bond. The conformation of this bond, connecting Loop 2 and Loop 6, appears to be elongase-specific. Alteration of this intramolecular interaction was also observed when comparing wild-type ELOVL4 and the p.W246G variant which causes SCA34. We demonstrate by sequence and structure analyses that ELOVL5 p.G230V and ELOVL4 p.W246G are position-equivalent missense variants. We conclude that SCA38 is a conformational disease and propose combined loss of function by mislocalization and gain of toxic function by ER/Golgi stress as early events in SCA38 pathogenesis.

**Supplementary Information:**

The online version contains supplementary material available at 10.1007/s00439-023-02572-y.

## Introduction

The spinocerebellar ataxias (SCAs) are group of genetic neurodegenerative disorders characterized by autosomal dominant inheritance, loss of cerebellar Purkinje cells and adult-onset, progressive ataxia (Ashizawa et al. 2018; Klockgether et al. 2019). To date (early 2023), about 50% of SCA patients have received a genetic diagnosis, leading to the gene-based identification of 50 types of SCA. Each SCA is characterized by a distinct mutation (missense, nucleotide repeat or deletion) in a specific protein-coding gene (Suppl. Table 1). Among the challenges posed by the SCAs are: (1) identifying the causative gene in undiagnosed cases; (2) understanding the unique vulnerability of cerebellar Purkinje cells (Huang and Verbeek 2019) and (3) elucidating the underlying pathogenic mechanisms with a view to (4) defining new therapeutic approaches.

We previously reported that SCA type 38 (SCA38) was caused by a missense variant (c.689G>T p.Gly230Val) in *ELOVL5* which encodes an ER transmembrane enzyme required for lipid biosynthesis (Di Gregorio et al. 2014). Absent in healthy humans, p.G230V was found in three Italian families with inherited adult-onset ataxia, the variant segregating with the disease across four generations in each family (Di Gregorio et al. 2014). The clinical manifestations of SCA38 begin around the fourth decade, with gait ataxia and nystagmus, progressing to upper limb ataxia, dysarthria, dysphagia, and ophthalmoparesis. (Borroni et al. 2016; Brusco et al. 2019; Di Gregorio et al. 2014). Among the distinguishing features of SCA38 are *pes cavus* (foot deformity with high arch) without paresis, hearing loss and hyposmia, and anxiety disorder (Brusco et al. 2019). Patients eventually become wheelchair-bound and treatment is supportive, although diet supplementation with docosahexaenoic acid (DHA) has been shown to slow disease progression (Manes et al. 2017, 2019). Neuropathology and brain imaging show Purkinje cell degeneration and cerebellar atrophy, while the brainstem and cerebral cortex appear normal (Borroni et al. 2016; Di Gregorio et al. 2014). ELOVL5 protein is expressed in the target organ, where it is found in Purkinje cell somata in the proximal part of the dendritic tree and also in deep cerebellar nuclei (Di Gregorio et al. 2014; Hoxha et al. 2017).

**Fig. 1:**
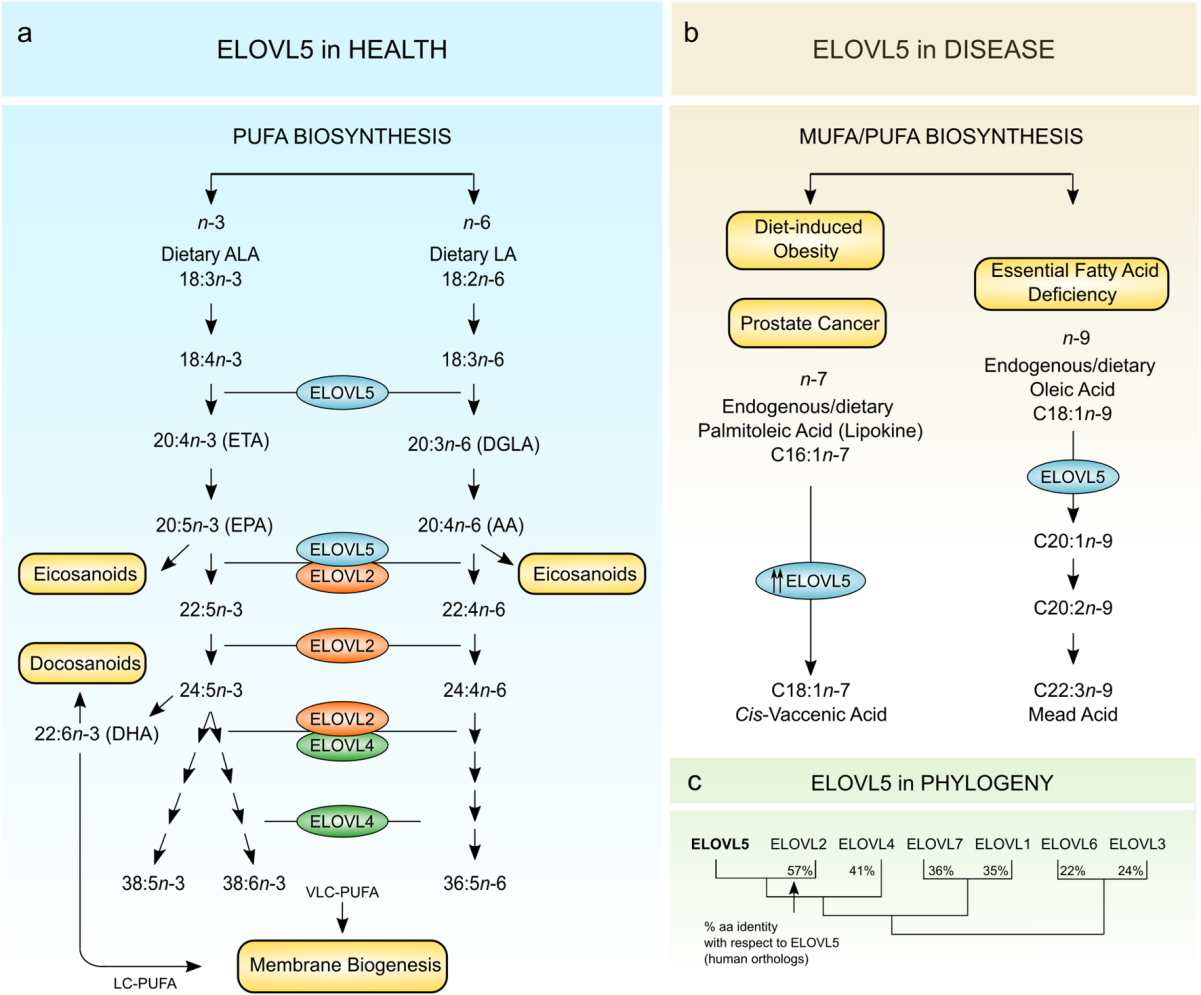
Outline of ELOVL5 activities in health and disease. **a** Schematic representation of PUFA biosynthetic pathways highlighting the role of ELOVL5 in converting C18 and C20 PUFA substrates in both n-3 and n-6 lineages. ALA, α-linolenic acid; LA, linoleic acid; ETA, eicosatetraenoic acid; EPA, eicosapentaenoic acid; DGLA, dihomo-γ-linolenic acid; and AA, arachidonic acid. PUFAs generated in these pathways are incorporated into structural lipids (membrane components) and functional lipids (bioactive mediators). **b** ELOVL5 also elongates substrates of the n-7 and n-9 series in non-physiological conditions such as: (i) essential fatty acid deficiency, where ELOVL5 had been reported to elongate C18:1n-9 to mead acid (C20:3n-9), which acts by replacing the missing PUFAs in membrane biosynthesis (Hayashi et al. 2020); (ii) in prostate cancer, ELOVL5 synthesizes the monounsaturated fatty acid *cis*-vaccenic acid (C18:1), which serves to maintain redox homeostasis (Centenera et al. 2021); in diet-induced obesity, murine Elovl5 regulates gluconeogenesis through the mTORC2-Akt-FOXO1 pathway (Hwang et al. 2018). **c** A multiple sequence alignment of human ELOVL1-7 was generated using the MAFFT alignment program, and used to produce a neighbour-joining phylogenetic tree illustrating the relationship between the human ELOVLs. The aa similarity matrix, generated using the SIAS (Sequences Identities and Similarities) program, showed the % aa sequence identity of the other human ELOVLs with respect to human ELOVL5

ELOVL5 (EC 2.3.1.199) is a long-chain fatty acyl-CoA elongase that resides in microsomal ER membranes together with numerous other enzymes that participate in the lipid biosynthetic pathway (Jacquemyn et al. 2017). ELOVL5 is broadly expressed (Ohno et al. 2010) but mostly studied for its role in liver lipid synthesis (Moon et al. 2001; Tripathy et al. 2014). ELOVL5 catalyzes the first elongation step in the synthesis of polyunsaturated fatty acids (PUFAs), adding a two-carbon unit to its substrates in the n-3 and n-6 pathways (Hoxha et al. 2017; Kihara 2012; Moon et al. 2009) (Fig. 1a). ELOVL5 is required for the synthesis of essential long-chain PUFAs (20–24 carbons) such as arachidonic acid (AA, C20:4n-6), eicosapentaenoic acid (EPA, C20:5n-3) and DHA (C22:6n-3) (Brenna and Kothapalli 2021). These PUFAs accrue in the brain, which has little capacity to synthesize them (Bazinet and Laye 2014; Lukiw and Bazan 2008), and are vital for maintaining structure and function of neuronal and glial cells (Bazinet and Laye 2014; Kerdiles and Calon 2017). Of note, of four SCA38 patients carrying the p.G230V variant and whose blood was available for analysis, we found significantly reduced serum DHA and AA with respect to healthy controls (Di Gregorio et al. 2014), and DHA supplementation with 600 mg/day was found to stabilize clinical symptoms and improve cerebellar metabolism (Manes et al. 2017, 2019). Although this effect may be addressing a deficit specific to SCA38, omega-3 treatment has been reported to be broadly beneficial in age-related cognitive decline, in other forms of ataxia and in neurodegenerative disorders (Kerdiles and Calon 2017; Ricca et al. 2020). Besides its physiological role as an enzyme, ELOVL5 is implicated in fatty acid deficiency (Hayashi et al. 2020), cancer (Boot et al. 2017; Centenera et al. 2021; Lee et al. 2020), and obesity-related diabetes (Hwang et al. 2018) (Fig. 1b).

In this study, we investigated the molecular mechanisms of Mendelian dominance triggered in SCA38 by p.G230V. A toxic gain of function hypothesis is suggested by our previous experiments in which heterologous expression of p.G230V consistently showed protein mislocalization and accumulation in the Golgi complex. In addition, we observed upregulation of the C/EBP homologous protein (CHOP) (Di Gregorio et al. 2014), a cell stress sensor induced by the unfolded protein response (UPR) leading to ER stress-induced apoptosis (Tabas and Ron 2011). The human ELOVLs are reported to form homo- and hetero-oligomers (Nie et al. 2021; Okuda et al. 2010) (Suppl. Figure 1) so dominant negative effects warrant consideration.

ELOVL5 is one of seven fatty acid elongases (ELOVL1-7) encoded in the human genome (Deak et al. 2019; Jump 2009). The ELOVLs are closely related paralogs (Fig. 1c; Suppl. Figure 2a; Suppl. Tables 1, 2) descended by gene duplication (Monroig et al. 2016) and part of a larger eukaryotic family of *ELO* domain (Pfam 01151)-containing elongases first described in yeast (Wallis et al. 2002). During evolution, the ELOVLs acquired distinctive individual properties in terms of substrate specificity and tissue expression, but all maintain the functional requirement for ER localization (Jacquemyn et al. 2017; Jakobsson et al. 2006; Ohno et al. 2010).

To date, only human ELOVL7 has been crystallized [Protein Data Bank (PDB) 6Y7F], revealing a barrel-shaped protein formed by seven transmembrane (TM) helices surrounding a central tunnel that accepts the acyl chains, with the fatty acid -CH_3_ end oriented towards the ER (Nie et al. 2021). The study further identified the residues critical for ELOVL catalysis and substrate binding, and revealed the catalytic mechanism. Here we compared p.G230V and wild-type ELOVL5 proteins by functional studies and protein modelling which point to alterations in organelle morphology, protein trafficking and protein conformation and stability, ultimately leading to ER stress and toxicity in vitro. In addition, we show by comparative protein modelling that the p.G230V causative variant of SCA38 is positionally equivalent to the (c.736T>G p.W246G) variant in *ELOVL4*, which causes SCA34 (Ozaki et al. 2015), thus identifying an elongase mutational hotspot and a shared conformational defect.

## Materials and methods

### Bioinformatic sequence analyses

The seven human ELOVL protein sequences were downloaded from the NCBI protein resource (https://www.ncbi.nlm.nih.gov/protein/), aligned using the MAFFT online program (v7.490) (Katoh et al. 2019) with the L-INS-I iterative refinement method, parameters set at BLOSUM62, gap opening penalty 1.53, offset value 0.0, and this alignment was used to generate a neighbour-joining tree using 192 conserved AAs and the JTT substitution model. To calculate the % sequence identity and similarity, we used the SIAS program (http://imed.med.ucm.es/Tools/sias.html) using the Blosum 62 matrix. The topological map of *Homo sapiens* ELOVL5 (NCBI/Uniprot accession no. Q9NYP7.1) was generated using the PROTTER program (Omasits et al. 2014) which incorporates the identification of N- and C-termini, and transmembrane domains. To generate the multiple sequence alignment (MSA) of ELOVL5, ELOVL2 and ELOVL4, a representative vertebrate dataset (mammals, birds, fish) of proteins was downloaded from the NCBI protein resource and aligned using MAFFT as described above. See Suppl. Table 3 for links to the webservers/databases used in this study.

### Cell culture, mammalian expression vectors and transfection

Primary fibroblasts were isolated from forearm skin biopsies obtained from a symptomatic 55-year-old female SCA patient carrying the (c.689G>T p.Gly230Val) variant and recruited from one of the Italian families described previously (Di Gregorio et al. 2014). As controls, primary fibroblasts were also isolated from three age- and origin-matched healthy individuals with their informed consent. After overnight incubation in Dulbecco’s modified Eagle’s medium (DMEM) supplemented with 10% FCS and 160 μg/mL collagenase, cells were cultured in DMEM with 10% FCS, 100 IU/mL penicillin, 50 μg/mL streptomycin, 2 mM glutamine, 1 mM sodium pyruvate and 1% non-essential amino acids (Thermo Fisher Scientific) at 37 °C, 5% CO_2_. Fibroblasts from passages 5 to 9 were used for all experiments, and cells from the SCA38 patient and healthy controls at the same passage were compared. The study was approved by the local Institutional Review Board (Comitato Etico Interaziendale A.O.U. Città della Salute e della Scienza di Torino). COS-7 transfected cells were obtained in our laboratory as previously described (Di Gregorio et al. 2014) and used as model systems for cell biology studies including western blot and imaging. Briefly, COS-7 cells (from laboratory ATCC-derived stocks) were grown in DMEM, 10% FCS, L-Glutamine (2 mM), 100 IU/mL penicillin and 50 μg/mL streptomycin.

Expression plasmids were prepared by cloning full-length wild-type human ELOVL5 cDNA in frame with a C-terminal HaloTag into the pReceiver-M50 Expression Clone (GeneCopoeia). This construct was modified by PCR-induced mutagenesis to introduce the c.689G>T point mutation in order to express the cDNA encoding p.G230V. Primers used were: 5′-gcacattccctcttgtttggttgtatttcca-3′ and 5′-tggaaatacaaccaaacaagagggaatgtgc-3′. Cells were transfected using Lipofectamine 2000 according to the manufacturer’s instructions (Life Technologies). Stable transfectants were selected in 700 μg/mL G418 (InvivoGen) started 24 h after transfection and maintained for 7 days.

### Enzymatic assay and lipid profiling

Primary hepatocytes were isolated from *Elovl5*^*−/−*^ mice livers according to published protocols (Moon et al. 2009). On day 0, cells were attached for 4 h before infection with adenovirus. Adenoviruses expressing either wild-type or p.G230V ELOVL5 were generated using ViraPower Adenoviral Expression System (ThermoFisher Scientific) and transduced into hepatocytes. On day 1, ELOVL5 substrate fatty acids C18:3, n-6 or C18:4, n-3 were added to the medium (final concentration 150 μM). On day 2, lipids were extracted from cells and analysed by gas–liquid chromatography as previously described (Moon et al. 2009), and whole cell lysates were prepared for protein analysis by western blot. Elongation activity was expressed as the ratio of C20:3, n-6 to C18:3, n-6, and of C20:4, n-3 to C18:4, n-3. Western blots were treated with anti-ELOVL5 rabbit polyclonal antibody (#C15621; 1:1000 dilution, Assay Biotechnology, Fremont, CA, USA) and IRDye 800 goat anti-rabbit IgG secondary antibody (Li-Cor Biosciences, Lincoln, NE, USA). Band intensities were measured using Li-Cor Odyssey infrared imaging system. For comparative analysis of hepatocyte lipid composition, on day 0 primary hepatocytes were isolated from wild-type and *Elovl5*^*−/−*^ mice. Cells were allowed to adhere for 4 h, followed by infection with adenovirus expressing either wild-type or p.G230V ELOVL5. No substrate fatty acids were added to the medium. On day 2, lipids were extracted from the cells and whole cell lysates were prepared for protein analysis, as described previously (Moon et al. 2009).

### Golgi apparatus immunofluorescence

Cells (5–8 × 10^4^) were seeded on fibronectin-coated coverslips in 24-well plates and maintained with complete DMEM medium at 37 °C. After 24 h, COS-7 transfected cells were incubated with HaloTag TMR Direct Ligand (Promega) at a final concentration of 1 μM. The Golgi area was immunostained with the mouse monoclonal anti-GM130 antibody (BD Bioscences, 6108223) and Alexa Fluor 488 goat anti-mouse secondary antibody (Life Technologies, A11017). Samples were analysed with an Olympus FV300 laser-scanning confocal microscope equipped with a blue argon (488 nm) laser, a green helium–neon (543 nm) laser, and FluoView 300 software (Olympus Biosystems). Cells were imaged with a 60 × oil-immersion objective (1.4 NA). Z-stack images of optical sections were digitally recorded, 3D reconstructed with ImageJ software and the cell surface area was measured for each cell. For each experimental point, at least 120 *cis*-Golgi acquisitions were analyzed and three technical replicas were performed. The results are shown graphically on an arbitrary scale (au).

### VSV infection and transport pulse protocol

To evaluate the impact of ELOVL5 expression on Golgi protein transit, COS-7 cells stably expressing empty pReceiver-M50 vector (mock), WT or G230V ELOVL5 were used. Infection of cells with vesicular stomatitis virus (VSV), synchronized transport of G-glycoprotein of VSV (VSVG) and immunofluorescence (IF) microscopy with a LSM510 laser scanning confocal microscope (Zeiss) were performed as described previously (Pulvirenti et al. 2008; Ruggiero et al. 2014). VSVG was stained using the anti-VSVG antibody P5D4 (Sigma, SAB4200695) after cell permeabilization (Pulvirenti et al. 2008). VSVG immunofluorescence was quantified by integration of the IF signal in the Golgi area and IF signal of the rest of the cell by ImageJ (Schneider et al. 2012). The Golgi area was defined by anti-GM130 antibody. Data are expressed as the ratio of IF between the Golgi and the rest of the cell at 5 min and 30 min after the release of the traffic block. All experiments were carried out in triplicate, and immunofluorescence was quantified in at least 20 cells (from three wells) per point per experiment. Sampling of cells was performed randomly.

### Protein analysis

To analyze the effects of proteosome inhibition on ELOVL5 protein expression, primary fibroblasts were plated (8 × 10^4^) in 12-well plates with complete DMEM medium at 37 °C. After 24 h, cells were treated with MG132 (2 μM) (M7449, Sigma) for 24 h. MG132 was dissolved in DMSO (Sigma). For ER stress induction, COS-7 cells were either untreated, treated with 2 μg/mL tunicamycin (Sigma) for 4 h at 37 °C, or treated with 1 µM thapsigargin (Sigma) for 24 h at 37 °C. Total proteins were extracted from fibroblasts or COS-7 cells after lysis in RIPA buffer (50 mM TrisHCl pH 8.0, 150 mM NaCl, 1% Triton X-100, 0.5% sodium deoxycholate, 0.1% SDS) supplemented with a protease inhibitor cocktail (Sigma). Proteins were suspended in 4 × LDS sample buffer and 10 × Sample Reducing Agent, according to the manufacturer’s instructions (Life Technologies). Equal amounts of protein extract (10–15 μg) were electrophoresed on 4–12% Bis–Tris Protein Gels (Life Technologies) and then blotted onto nitrocellulose (Bio-Rad) in Tris/glycine buffer with 20% methanol at 4 °C for 90 min. Bands were detected with WesternBreeze™ Chemiluminescent Detection Kit (Invitrogen, Thermo Fisher Scientific) followed by image capture with a ChemiDoc™ XRS+ System and densitometry analysis with Image Lab™ Software (Bio Rad). The following primary antibodies were used in the experiments: ELOVL5 (Assay Biotech, C15621, 1:500); XBP-1 (Santa Cruz Biotechnology, M-186, 1:200); ATF4 (Santa Cruz Biotechnology, sc-200, 1:3000) CHOP (Cell Signaling, P35638,1:1000), vinculin (Millipore, AB6039, 1:5000). Anti-HaloTag (Promega, G928A, 1:1000) was used to detect the HaloTag protein in stable transfected cell lines.

### RNA extraction and analysis

Total RNA was extracted using Direct-Zol RNA MiniPrep system (Zymo Research); genomic DNA was removed by treatment with DNase I (Sigma-Aldrich), according to the manufacturer’s protocol. The cDNAs were generated using the M-MLV Reverse Transcriptase kit (Invitrogen, Thermo Fisher Scientific). Expression of *ELOVL5* was measured by quantitative PCR using the FAM-labeled Universal Probe Library system (UPL#31, Roche Diagnostics) and specific primers (forward: 5′-cccttccatgcgtccata-3′; reverse: 5′-gattgtcagcacaaactgaagc-3′). VIC-labeled pre-designed TaqMan gene expression assay for TATA-Binding protein (TBP) (Applied Biosystems) was used as normalizer. Reactions were carried out in triplicate on an ABI 7500 Fast real-time PCR machine using the ABI 2 × TaqMan Universal PCR Master Mix II, according to the manufacturer’s instructions (Thermo Fisher Scientific). XBP1 splicing was analysed by semi-quantitative PCR (5 min at 98 °C; 35 cycles of 95 °C for 30 s, 60 °C for 30 s, 72 °C for 30 s; 72 °C for 5 min) using KAPA2G Fast HotStart Taq (Merck). To detect the spliced and unspliced forms of XBP1 (XBP1u/s) and the endogenous control GAPDH, the following primer pairs were used: XBP1u/s forward 5′-gccgggtctgctgagtcc-3′, reverse 5′-tgactgggtccaagttgtcc-3′; GAPDH forward 5′-accatcttccaggagcgaga-3′, reverse 5′-gggccatccacagtcttctg-3′. Primers were designed based on African green monkey sequences. PCR products were loaded on a 2% agarose gel (1 × Tris–Borate-EDTA Buffer, 2% agarose, 0.1% EuroSafe Nucleic Acid Stain) using 6 × purple gel loading dye (New England BioLabs). Images were acquired using the ChemiDoc Imaging System (BioRad) and bands were quantified using the Image Lab (BioRad) volume tool.

### Toxicity assay and immunofluorescence analysis of primary mouse cortical neurons

Primary cortical neurons were cultured from embryonic E15.5 C57BL/6J mice as previously described (Basso et al. 2012; Tripathy et al. 2017). Primary cortical neurons were seeded in 96-well tissue culture plates at a concentration of 2.5 × 10^4^ cells/well. The human ELOVL5 cDNA was inserted into the SYN-GFP vector (LentiLox 3.7 backbone) using primers containing selected restriction sites (5′-ggtggatccatggaacattttgatgca and 5′-accgcatgctcaatccttccgcagctt), under the control of the synapsin I promoter to generate the Syn-eGFP_ELOV5 WT and mutant plasmids. At DIV1, immature neurons were transduced with viral particles (at MOI 1) expressing only GFP (Mock) or GFP with ELOVL5-WT or G230 variant. At day in vitro (DIV) 7, neurons were fixed in 4% paraformaldehyde and processed by immunocytochemistry for following washes after the secondary antibodies (anti-rabbit Alexa568-Thermo Fisher, A10042, 1:1000; anti-mouse Alexa488-Thermo Fisher, A32766, 1:1000), cells were incubated with Hoechst dye (1:1500 in PBS) for 10 min. Plates were imaged with OperettaTM High-Content Screening system (PerkinElmer). In each well, images were acquired in 12 preselected fields with an LWD 20 × objective over three channels with the appropriate filter settings and analyzed using Harmony software version 4.1 (PerkinElmer). In each assay, the total number of GFP^+^ or MAP2^+^ live neurons in each condition was counted. Experiments were carried out in triplicate and repeated at least three times independently. For immunofluorescence analysis, neurons were grown on poly-d-lysine coated coverslips. At DIV8, the coverslips were fixed using 4% paraformaldehyde, cells were permeabilized with PBS 0.1% Triton X-100 for 5 min, blocked with PBS 10% FBS, 0.05% Triton X-100 for 1 h and then incubated overnight with the primary antibodies diluted in PBS 0.01% FBS. The next day, after three washes in PBS, the Alexa-Fluor conjugated secondary antibodies (1:1000) in PBS 0.01% FBS were added for 1 h in the dark. The coverslips were then washed with PBS and mounted on glass slides with Prolong Gold antifade mounting media containing the nuclear stain DAPI (Life Technologies). Slides were imaged with a Zeiss AxioObserver.Z1 microscope. The following antibodies were used in the experiments: ELOVL5 (AssaybioTech, C15621, 1:500), GFP (Merck-Millipore, MAB3580, 1:1000), MAP2 (Merck-Millipore, AB5622, 1:500) and the Thermo Fisher Scientific’s Alexa Fluor secondary antibodies (Alexa568 anti-rabbit A10042; Alexa488 anti-mouse A32766; Alexa633 anti-chicken A21103).

### In silico protein modelling and stability prediction

3D models of human ELOVL5 (wild-type and variant), ELOVL4 (wild-type and variant) and ELOVL2 were generated by homology to human ELOVL7 (PDB 6Y7F) and pdb files generated using Protein Homology/analogY Recognition Engine (PHYRE) version 2 (Kelley et al. 2015). To generate overlapping 3D structures for comparative analysis, pdb files (available upon request) were imported into UCSF Chimera (alpha version 1.16, build 42318) (Pettersen et al. 2004). Disulfide bond lengths were also measured in Chimera. To predict changes in protein stability, we used the following three online tools: I-Mutant2.0 (Capriotti et al. 2005), MAESTRO (Laimer et al. 2015), INPS-MD (Savojardo et al. 2016) and SAAFEC-SEQ (Li et al. 2021). Links to the webservers used are available in Suppl. Table 1.

### Statistical analysis

Data are expressed and statistical analyses were performed as described in the figure legend for each analysis. Statistical significance was determined using Prism 7 software as indicated. A *p* value of < 0.05 was considered statistically significant (*p* ≤ 0.05).

## Results

### Assessing the potential impact of p.G230V on ELOVL5 structure and function

**Fig. 2:**
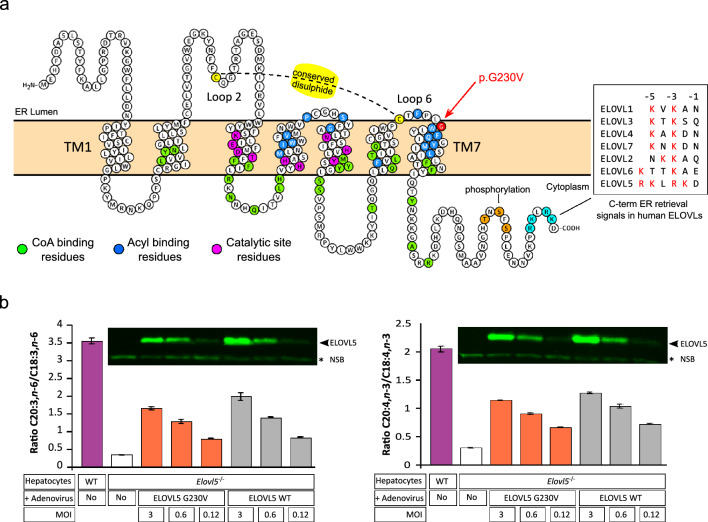
Schematic structure of ELOVL5 protein and comparative analysis of enzymatic activities of wild-type ELOVL5 and p.G230V **a** Two-dimensional prediction of ELOVL5 protein structure using the Protter program, showing the main structural and functional features including the seven transmembrane domains (TM1 and TM7 are labelled) and the intervening loops (Loop 2 and Loop 6 are labelled). The position of G230 is indicated by the red arrow. The color-coded features listed on top right are: Phos (orange residues), showing the three putative phosphorylation sites (T281, S283, S285) reported to regulate ELOVL5 activity and protein stability (Hayashi et al. 2020); ER signal (blue), C-terminal residues that form dibasic retrieval signals (red residues, shown in detail in lower right box); G230V (red), residue 230 mutated in SCA38; disulphide (yellow), the elongase-conserved intramolecular disulphide bond formed between Cys96 and Cys225; CoA (green), residues that interact with the CoA component of the substrate (Nie et al. 2021); Acyl (blue, white text), residues that interact with the acyl component of the substrate (Nie et al. 2021); Catalytic (crimson), residues that regulate the catalytic activity, including the histidine motif. The transmembrane domains were detected by the Phobius program within Protter. **b** Analysis of ELOVL5 elongation activity in mouse hepatocytes isolated from WT (*Elovl5*^+*/*+^) and *Elovl5*^−/−^ mice, incubated with C18 substrate (C18:3, n-6 left panel; C18:4, n-3 right panel). Hepatocytes from *Elovl5*^−/−^ mice were transduced with adenoviruses to express either human wild-type (WT) ELOVL5 or p.G230V (G230V). Hepatocytes were infected with decreasing amounts of adenovirus (MOI, multiplicity of infection). Insert above the histograms shows a representative western blot showing WT and p.G230V expression in hepatocyte transfectants used for lipid analysis. *Non-specific band used as loading control

We first generated a 2D map of ELOVL5 to locate G230 within the context of the principal topological hallmarks of the protein (Fig. 2a) which are: (1) the seven transmembrane helical domains (TM1-TM7) and their six intervening loops (Loop 1-Loop 6); (2) the His motif (HxxHH) catalytic site; (3) two cysteine residues, C96 and C225, which are highly conserved among eukaryotic elongases and form an intramolecular disulphide bond connecting Loop 2 and Loop 6, facing towards the ER lumen; (4) the dibasic (lysine/arginine) ER retention/retrieval signals in the C-terminal tail and (5) three putative phosphorylation sites (T281, S283, S285) reported to regulate ELOVL5 activity and protein stability (Hayashi et al. 2020).

G230 is located at the junction where Loop 6 ends and joins TM7. The region encompassing TM6/Loop 6/TM7 is known to be important for substrate preference, as previously demonstrated by Denic and Weismann (Denic and Weissman 2007). They showed that a yeast elongase homolog could accommodate shorter or longer fatty acid substrates by shortening or lengthening the TM6/TM7 helices, which act as a molecular calliper gauging PUFA length. The ELOVL7 3D crystallographic structure added a further level of definition, describing 51 residues responsible for catalysis and substrate binding in the human ELOVLs (Nie et al. 2021). These residues include: (1) 10 residues involved in catalysis, all 10 conserved in human ELOVL1 to ELOVL7, indicating they are essential for biological function; (2) 25 residues that interact with the CoA substrate; and (3) 16 residues required for interaction with the acyl substrate (Fig. 2a). However, G230 or its equivalent residue in the alignment with the other human ELOVLs was not among these 51 residues responsible for catalysis or substrate binding, suggesting that the p.G230V missense substitution may impact upon structure rather than enzymatic activity.

### p.G230V is not biochemically disruptive

To examine whether or not the p.G230V variant affected ELOVL5 catalytic activity, we compared elongase activity of wild-type and p.G230V variant proteins individually expressed in hepatocytes obtained from *Elovl5*-deficient mice, an established model for the study of ELOVL5 activity (Moon et al. 2009). At 24 h post-adenoviral transduction, the ELOVL5 substrates γ-linolenic acid (C18:3, n-6) and stearidonic acid (C18:4, n-3) were added to the culture medium. After 24 h incubation, lipid fractions were extracted and analyzed by gas–liquid chromatography to measure conversion to C20 products, as previously described (Moon et al. 2001). The results were expressed as the C20:C18 ratio (Fig. 2b), and showed no significant difference in elongase activity between wild-type and p.G230V-expressing hepatocytes. In addition, the ratios decreased proportionally with the decrease in expression of transfected protein, confirming the assay detected ELOVL5 elongase activity. These results were corroborated by profiling the fatty acid composition of hepatocyte transfectants where no major differences emerged between wild-type and p.G230V transfectants (Suppl. Figure 3).

### p.G230V overexpression enlarges the Golgi apparatus and alters ER-Golgi trafficking

Residency of ELOVL5 within microsomal ER membranes is essential for its function (Jacquemyn et al. 2017; Logan et al. 2014), and is likely obtained by a combination of mechanisms such as retention through physical properties of the TM domains (Cosson et al. 2013) and retrieval from the Golgi apparatus through the COPI-binding C-terminal dibasic motif (Kokubun et al. 2019). In fact, loss of this motif in ELOVL4 by deletion results in Stargardt Disease 3 (STGD3; MIM #600110) (see below). Although the p.G230V substitution does not affect the retrieval motif, we have previously demonstrated by heterologous expression and confocal fluorescence microscopy that ELOVL5 G230V staining co-localized with the *cis*-Golgi marker GM130 (Di Gregorio et al. 2014).

**Fig. 3:**
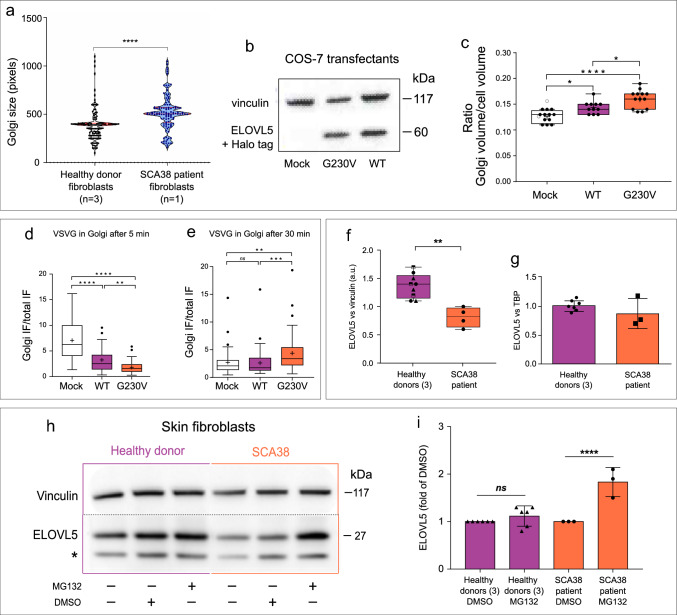
Effects of p.G230V expression on Golgi size, intracellular trafficking and proteasomal inhibition **a** violin plot of Golgi complex size in SCA38-derived and fibroblasts derived from three healthy donors determined by GM130 Golgi marker staining. Each dot represents a single cell. Red lines: median, 25th to 75th percentiles. ****p < 0.0001 (two-tailed, Mann–Whitney test). **b** Western blot analysis of ELOVL5 protein expression in COS-7 cells transfected with empty vector control (mock), Halo-tagged wild-type (WT) or p.G230V showing that the relative ELOVL5 overexpression dosage is comparable in WT versus G230V-transfected cells. Vinculin was used as loading control. **c** Golgi complex volume in COS-7 cells transfected with empty vector control (mock), or Halo-tagged WT or p.G230V ELOVL5. Confocal microscopy Z-stack images were 3D-reconstructed with ImageJ software. Data are expressed as the ratio between the volume of the Golgi and the rest of the cell, and are plotted as the pooled results of three independent experiments. For each technical replicate, at least 130 *cis*-Golgi acquisitions/condition were analysed. Each dot is the mean value of at least 30 measurements per field. Center line: median; box limits: 25th to 75th percentiles; whiskers, min to max. *p < 0.05, ****p < 0.0001 (ANOVA with Tukey’s multiple comparisons test). (d,e) Box plot showing the ratio between Golgi fluorescence and total cell fluorescence in COS-7 transfectants (empty vector control, WT or p.G230V) infected with VSV ts045 temperature-sensitive mutant. Cells were shifted to the permissive temperature of 32 °C and fixed after 5 min or 30 min followed by staining with anti-VSVG antibody. VSVG immunofluorescence was quantified by integration of the IF signal in the Golgi and IF signal of the rest of the cell by ImageJ. The Golgi area was defined by staining with anti-GM130 antibody. Data are expressed as the ratio of IF between the Golgi and the rest of the cell at 5 min (**d**) and 30 min (**e**) after the release of the traffic block. Data are plotted as the pooled results of three independent experiments in which IF was quantified in at least 20 cells (from three wells) per point per technical replicate. Centre line: median; box limits: 25th to 75th percentile; whiskers: inter-quartile range; circles represent the outliers. ****p < 0.0001;***p < 0.001, **p < 0.01 (non-parametric Kruskal–Wallis test followed by a Dunn’s multiple comparisons test). **f** Analysis of ELOVL5 expression in untreated SCA38-derived skin fibroblasts and healthy donor fibroblasts (see representative bands in lanes 1 and 4 of panel **h**). Band intensities relative to vinculin were quantified using ImageLab. Different symbols indicate fibroblasts from different healthy donors. **p < 0.001 (unpaired *t* test). **g** Quantitative real-time PCR analysis of ELOVL5 mRNA expression in SCA38 skin fibroblasts and healthy donor-derived fibroblasts. TATAA box-binding protein (TBP) was used as reference gene. **h** SCA38-derived skin fibroblasts and healthy donor-derived fibroblasts treated with the proteasome inhibitor MG132 (2 μM, 24 h), the vehicle DMSO or left untreated were analysed by western blot for ELOVL5 expression. The asterisk indicates an uncharacterized immunoblot signal, as previously reported (Di Gregorio et al. 2014). Vinculin was used as loading control. Shown is a representative western blot of several performed. **i** ELOVL5 expression was determined by densitometry and plotted as the fold change relative to DMSO (mean ± SD of technical replicates, *n* = 3–6), normalized to the corresponding vinculin. ****p < 0.0001 (Sidak’s multiple comparison test)

To examine the potential impact of the p.G230V variant on Golgi size, primary skin fibroblasts were derived from a SCA38 patient carrying the (c.689G>T p.Gly230Val) substitution, and from three different healthy donors. Golgi size was determined by GM130 staining and confocal microscopy analysis. SCA38-derived fibroblasts showed a significant increase in Golgi size with respect to healthy controls (Fig. 3a).

To verify that expression of p.G230V impacted on Golgi size, COS-7 cells were transfected with Halo-tagged p.G230V or wild-type ELOVL5 (Fig. 3b), and the Golgi volume was quantified by fluorescent confocal 3D reconstruction. The Golgi volume was significantly greater in p.G230V transfectants than in wild-type, with the caveat that overexpression of wild-type ELOVL5 also led to some increase in Golgi size with respect to mock-transfected cells (Fig. 3c).

To analyse the impact of p.G230V expression on anterograde ER-to-Golgi traffic, we monitored the passage of the vesicular stomatitis virus G protein (VSVG) through the Golgi by co-localization with the *cis*-Golgi marker GM130 by immunofluorescence. The VSVG assay was performed using COS-7 cells stably expressing vector only, p.G230V or wild-type ELOVL5, and infected with the VSVG ts045 temperature-sensitive variant. At 40 °C, proteins are unfolded and retained in the ER but fold correctly and exit the ER in a synchronous wave when the temperature is lowered to the permissive temperature of 32 °C, commuting rapidly to the Golgi complex. After 5 min from removing the traffic block, a large amount of VSVG reached the Golgi in mock-transfected cells, while a smaller amount was transported to the Golgi in wild-type ELOVL5 cells and even less in p.G230V cells (Fig. 3d). This suggests a possible alteration of ER functions following overexpression of ELOVL5. After 30 min, the amount of VSVG in the Golgi was significantly higher in p.G230V cells than in mock and wild-type ELOVL5 cells (Fig. 3e), suggesting altered Golgi transport function in cells overexpressing ELOVL5 p.G230V. Transport of VSVG to the plasma membrane was unaffected in both transfectants (data not shown).

### ELOVL5 protein expression is restored by proteasomal inhibition

Under physiological conditions, ⁓ 15% of newly synthesized proteins are misfolded and destroyed by the ubiquitin–proteasome system (UPS), with the percentage increasing in the presence of conformationally mutated proteins (DePristo et al. 2005; Sun and Brodsky 2019).

To analyse the impact of p.G230V on ELOVL5 protein degradation by the UPS, SCA38- and healthy donor-derived fibroblasts were treated with 2 μM MG132 proteasome inhibitor for 24 h and ELOVL5 protein levels were analysed by western blot. In untreated SCA38 p.G230V fibroblasts, total ELOVL5 protein was reduced by ~ 40% with respect to healthy donors (Fig. 3f). By quantitative RT-PCR, global ELOVL5 mRNA expression levels were not significantly different between SCA38 and healthy fibroblasts (Fig. 3g). Treatment with MG132 considerably increased ELOVL5 protein levels with respect to DMSO vehicle control but did not significantly change ELOVL5 expression in fibroblasts from healthy donors (Fig. 3h, i). These results suggest that by reducing p.G230V protein degradation, MG132 treatment increased the ELOVL5 signal in the SCA38 sample. Attempts to confirm this in COS-7 cells with single-allele transfectants was not possible due to MG132 toxicity (data not shown).

### ELOVL5 G230V activates the unfolded protein response

Accumulation of misfolded proteins in the ER triggers membrane-bound stress sensors that activate signalling pathways known as the unfolded protein response (UPR), whose goal is to decrease protein load in the ER and increase the protein folding capacity (Moore and Hollien 2012; Scheper and Hoozemans 2015; Walter and Ron 2011).

**Fig. 4:**
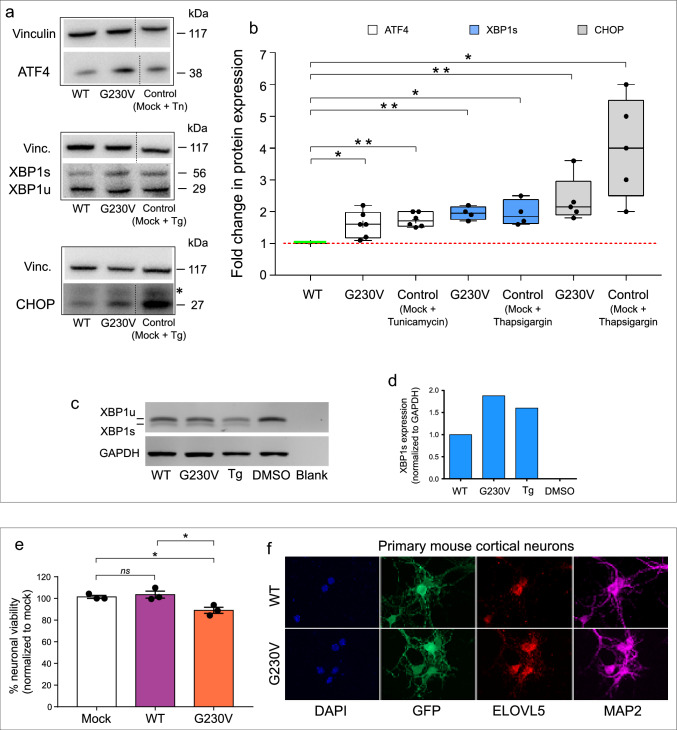
ER stress and neuronal toxicity induced by p.G230V. **a** Representative western blots of several performed showing expression of UPR markers ATF4, XBP1 and CHOP in COS-7 cells stably expressing WT ELOVL5 or p.G230V. Tunicamycin (TM)-treated mock cells (20 μg/μl for 4 h at 37 °C) and thapsigargin (TG)-treated cells (1 μM for 24 h at 37 °C) were used as controls for ER stress induction and UPR activation, respectively. Vinculin was used as loading control. **b** ATF4, XBP1 and CHOP protein expression was determined by densitometry and plotted as fold change of expression of each protein relative to its expression in COS-7/WT ELOVL5, normalized to the corresponding vinculin. Relative fold change values from all blots were displayed as a scatter plot. The control sample (COS-7/WT ELOVL5) is represented by a green horizontal line. Technical replicates of samples are shown (*n* = 4–6) **p < 0.01; *p < 0.05 (ANOVA followed by a Dunnett’s multiple comparisons test). **c** Representative image of semi-qPCR analysing XBP1 unspliced and spliced (XBP1 u/s) isoforms known to differ by 26 bp but easily distinguishable after a 40 min electrophoresis in 2% agarose gel. Lanes 1–2 show COS-7/WT and COS-7/G230V, respectively; lanes 3–4 show COS-7 cells treated with thapsigargin (TG) as positive control of UPR induction and DMSO as vehicle control. Lane 5, blank negative control. **d** Histogram showing XBP1 spliced isoform normalized to GAPDH expression. **e** Analysis of cell viability in mouse primary cortical neurons expressing only SYN-GFP vector (Mock) or GFP fused to wild-type ELOVL5 (WT) or p.G230V. Graph represents mean ± SEM. Experiments were carried out in triplicate (12 images/well) and repeated three times independently. Median value of each technical replicate is shown. *p < 0.05, 1-way ANOVA with Tukey’s post hoc test. **f** Primary mouse cortical neurons transduced with lentiviruses expressing GFP and ELOVL5 protein (WT or G230V variant). Cells were stained with anti-Microtubule Associated Protein 2 (MAP2) as a marker of mature neurons

To see if p.G230V expression leads to activation of the UPR, we analysed expression by western blot of the canonical UPR sensors ATF4, XBP1s and CHOP in COS-7 cells stably expressing wild-type or p.G230V ELOVL5. Expression of ATF4, XBP1s and CHOP was increased in cells expressing G230V with respect to cells expressing wild-type ELOVL5. As controls, the UPR was pharmacologically induced in mock-transfected COS-7 cells by treatment with the ER stressors tunicamycin or thapsigargin (Fig. 4a, b).

The spliced/unspliced status of XBP1 was also analysed by semi-quantitative PCR (Creedican et al. 2019), showing increased XBP1s in cells expressing p.G230V similar to the effect on XBP1s expression induced by the control ER stressor thapsigargin (Fig. 4c, d). These results suggest that ELOVL5 G230V expression induces ER stress and activation of the UPR.

### ELOVL5 G230V is toxic in cortical neurons

The preceding results demonstrate that p.G230V undergoes both abnormal intracellular trafficking and toxic ER accumulation in the principal in vitro cellular models used here. However, to evaluate if p.G230V expression is toxic in cells of neuronal origin, we adopted an established model using primary mouse cortical neurons (Tripathy et al. 2017). Following isolation and culture, on day in vitro 1 (DIV1), cells were transduced with lentiviruses expressing GFP and an ELOVL5 protein (wild type or mutant) through two independent synapsin I promoters. Neuronal viability was evaluated on DIV7 by quantifying fluorescent neurons using the Operetta High Content Imaging System. The results show that expression of wild-type ELOVL5 did not affect neuronal viability whereas expression of p.G230V decreased neuronal viability by 10% (Fig. 4e), indicating a slight but significant neurotoxic effect of p.G230V.

The transduced cells were further stained with antibody against the mature neuron marker Microtubule Associated Protein 2 (MAP2) to confirm the differentiated phenotype of the cells. ELOVL5 and GFP expression was verified by confocal microscopy acquisition, that showed a good transduction rate in MAP2 positive neurons (Fig. 4f).

### Absolute evolutionary conservation of Gly230 in ELOVL5 and in its closest paralog ELOVL2

**Fig. 5:**
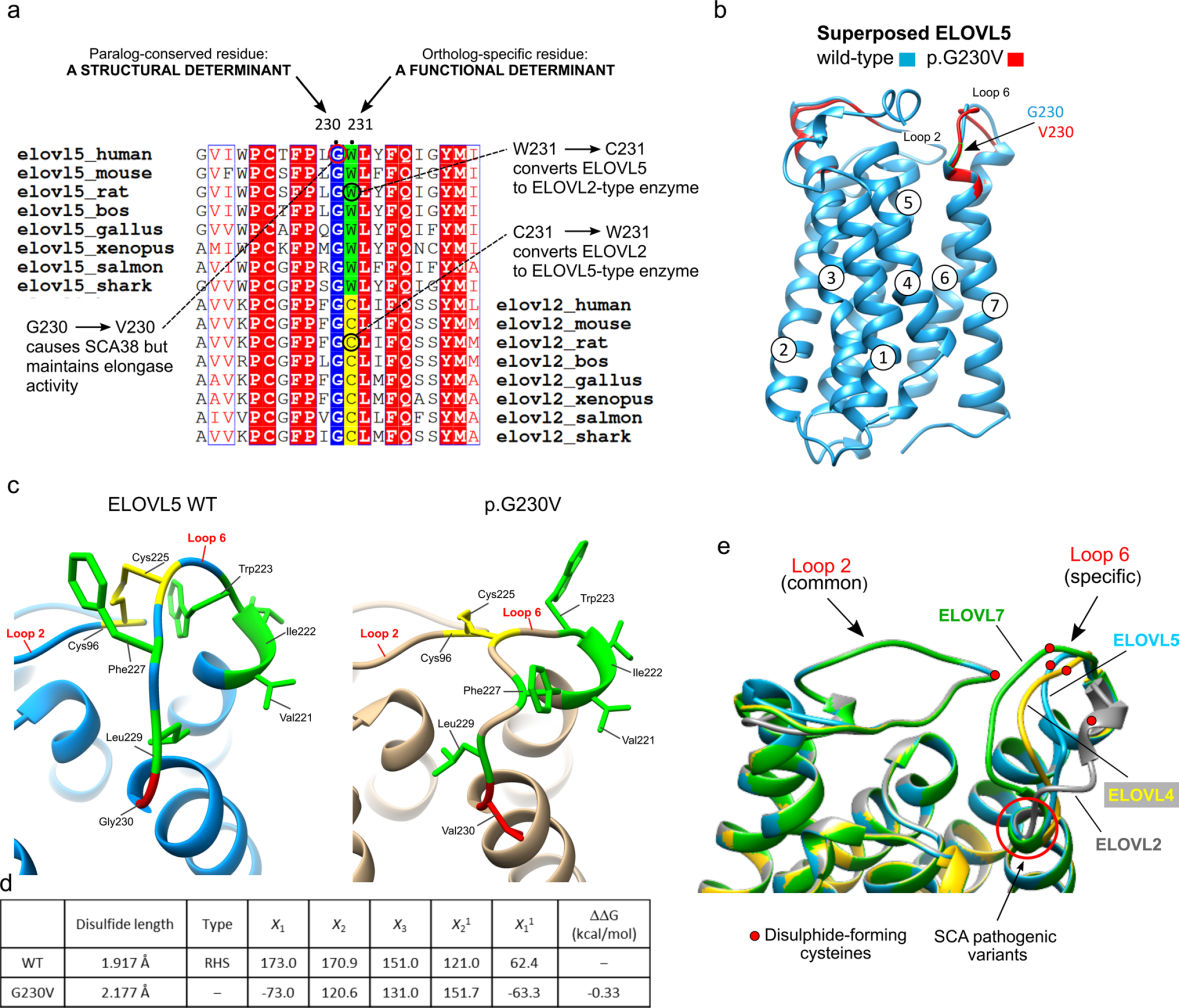
Evolutionary conservation of G230 in ELOVL5 and ELOVL2, and impact of p.G230V on protein conformation. **a** Relevant section of the multiple sequence alignment (MSA) of vertebrate ELOVL5 orthologs and its closest paralog ELOVL2. The full MSA is available in the Supporting Information. The MSA highlights that G230 is common to the paralogs ELOVL5 and ELOVL2, suggesting its relevance for structural integrity; the adjacent residue (ELOVL5 W231; ELOVL2 C217) is ortholog-specific, pointing to a functional role specific to a particular ELOVL protein. This is supported by the fact that changing Elovl5 W231 (in rat) to C231 modifies the substrate preference of ELOVL5 to that of ELOVL2, and vice versa (Gregory et al. 2013). **b** Overlay of the 3D structures of wild-type (WT) ELOVL5 (blue) and p.G230V (red) proteins highlighting the position of G230V, the correct superposition of Loop 2 which contrasts with the shift in position of Loop 6. **c** Enlargement of Loop 2/Loop 6 intramolecular bond, showing how the disulphide bond changes in p.G230V (in gold, on right) with respect to the bond in the wild-type protein (WT, blue, on left). Hydrophobic residues of Loop 6 are shown in green to highlight the positional change of their side chains. The conserved cysteines that form the disulphide bond are shown in yellow. **d** Table illustrates the impact of G230V substitution on the length and form of the conserved disulphide bond, and on protein stability. The length of the bonds were calculated and visualized in UCSF Chimera. The PDB files of wild-type and p.G230V were uploaded in the PDBsum home page (http://www.ebi.ac.uk/thornton-srv/databases/pdbsum/) to generate protein structural details including disulphide bond conformation. The program calculated the conformation of the conserved disulphide bond in wild-type and p.G230V ELOVL5, measuring the five dihedral angles (*X*_1_, *X*_2_, *X*_3_, *X*_2_^1^ and *X*_2_^2^) that form this bond. The conformation favoured by wild-type ELOVL5 is the right-handed spiral (RHS), which is lost in p.G230V. Negative free energy variation indicates a loss of protein stability, the result shown here was calculated with SAAFEC-SEQ. **e** The protein structures of native ELOVL5 (blue), ELOVL7 (green), ELOVL4 (yellow) and ELOVL2 (grey) were superimposed and zoomed to highlight the shared conformation of Loop 2 in the four elongases, in contrast to the individual ELOVL-specific conformation of Loop 6. The red dots represent the position of the conserved Cys residues that form the intramolecular disulphide bond. The position of the pathogenic variants ELOVL5 G230V and ELOVL4 W246G are shown (red arrow)

Taken together, the functional assays excluded an intrinsic defect in catalytic activity of p.G230V and showed toxic effects mediated by p.G230V, pointing to a conformational disorder. The evolutionary conservation of the wild-type residue is one of the main criteria used to calculate the pathogenic potential of a missense variant (Iqbal et al. 2020; Richards et al. 2015). A multiple sequence alignment (MSA) was generated using a representative dataset of 30 ELOVL5 orthologs from the vertebrate lineage, corresponding to the phylogenetic distribution of *ELOVL5* (Monroig et al. 2016). The MSA shows absolute conservation of G230 among ELOVL5 orthologs (Fig. 5a; Suppl. Tables 4, 5).

*ELOVL5* originated from the duplication of a common *ELOVL2/5* ancestral gene at the vertebrate root (Monroig et al. 2016), and ELOVL5 and ELOVL2 are reciprocal closest paralogs (57% aa identity; 71% aa similarity). In agreement with current models of enzyme evolution (Yang et al. 2020) and as demonstrated by Monroig et al*.* (Monroig et al. 2016), ELOVL5 and ELOVL2 ancestrally catalysed the same reactions but evolved to catalyse successive metabolic steps, with ELOVL2 preferring C20–C24 substrates. The elegant work of Gregory et al*.*, using chimeric proteins and point mutations, succeeded in changing the substrate preference of ELOVL5 (rat) from C18 to C22 PUFAs, by changing just one amino acid: Trp231 (Gregory et al. 2013). This is one of the 16 ELOVL residues identified by Nie et al*.* as involved in acyl binding (Nie et al. 2021). Alignment of ELOVL5 and ELOVL2 proteins again showed absolute conservation of a glycine residue in ELOVL2 that aligned with G230 in ELOVL5 (Fig. 5a; Suppl. Tables 6, 7), defining G230 as a paralog-conserved residue. This rigid conservation is highly significant because mutating a paralog-conserved residue risks being more deleterious than changing a site that is not paralog-conserved (Lal et al. 2020; Patthy 2008).

As ELOVL5 and ELOVL2 are likely to be very similar in 3D structure but distinct in their substrate preference, their shared evolutionary constraint for this position-specific glycine again points to a requirement for a common structural feature (Patthy 2008). In protein topology, glycine is a special amino acid because it only has a hydrogen atom as side chain, affording flexibility and allowing glycine to occupy positions in protein structures that are forbidden to all other amino acids (Barnes and Gray 2003). Unlike glycine, valine is bulky, branched and hydrophobic and the substitution is predicted to modify protein hydrophobicity by ProtScale analysis (Gasteiger et al. 2003) (Suppl. Figure 2b), likely affecting the tight turn formed by Loop 6 as it joins TM7.

### p.G230V shows a shift in the conserved intramolecular disulphide bond

Exploiting the principle that homologous proteins show similar 3D structures, we generated the structure of ELOVL5 by homology modelling to its paralog ELOVL7 (PDB 6Y7F) (Nie et al. 2021). Human ELOVL5 and ELOVL7 show 36% overall aa sequence identity, rising to 43% identity/60% similarity in the *ELO* domain. The models of wild-type and p.G230V ELOVL5 shown here (Fig. 5b) were obtained using the Phyre2 protein modelling program (Kelley et al. 2015) (81% of the sequence modelled with 100% confidence) but other modelling methods such as Tasser (Yang et al. 2015), Swiss Model (Waterhouse et al. 2018), and the pre-compiled AlphaFold (Jumper et al. 2021) gave highly similar results (data not shown).

When wild-type and p.G230V protein structures were superimposed, they almost completely overlapped except for an evident positional shift in Loop 6 in the variant protein (Fig. 5b, c). Positional changes in Loop 6 can have potentially profound consequences. ELOVL5 has a cytoplasmic face, where the methyl (*ω*) end of the acyl chain enters the substrate tunnel, which is closed at the ER end by a disulphide link between C96 in Loop 2 (connecting TM2-TM3) and C225 in Loop 6 (connecting TM6-TM7). As mentioned, in eukaryotic elongases, these disulphide-forming cysteines are highly conserved, their typical function being to support structural stability (Bechtel and Weerapana 2017; Dombkowski et al. 2014; Wiedemann et al. 2020). The ER is endowed both with a redox buffering environment and proteins dedicated to disulphide bond formation and protein folding (Matsusaki et al. 2020; Oka and Bulleid 2013). The positional shift in Loop 6 in p.G230V moves C225 away from C96 (whose position remains unaltered as for all of Loop 2), the end result being that the disulphide bond increases in length from 1.917 Å in wild-type to 2.177 Å in p.G230V, as measured using the UCSF Chimera program for the analysis of molecular structures (Pettersen et al. 2004). In addition, the bond angles between the two half-cystines are modified and no longer form a right-handed spiral, indicating stressed torsional angles (Fig. 5d). Presuming the unique disulphide bond in ELOVL5 endows the protein with greater stability, we analysed the change in fold stability (ΔΔG) caused by the G230V substitution using four different machine learning methods: I-mutant (decreased stability: − 0.19 kcal/mol), Maestro (decreased stability: + 0.15 where > 0.0 is destabilizing), INPS-MD (decreased stability: − 0.7 kcal/mol) and SAAFEC-SEQ (decreased stability: − 0.33 kcal/mol). In all cases, the ΔΔG of the variant protein showed a small but consistent-across-programs decrease in stability (Fig. 5d). Most disease variants modify protein stability by 1–3 kcal/mol (Yue et al. 2005)and variations < 0.5 kcal/mol are to be considered as fully disruptive (Capriotti et al. 2008). The fact that the overall stability is moderately (but consistently) predicted to be affected supports our results showing conserved biochemical function but altered trafficking, ER stress and proteasome inhibition data.

To further investigate the relevance of the conformational change in p.G230V, next we generated protein structures for ELOVL2 and ELOVL4, the two closest paralogs to ELOVL5. ELOVL4 was also modelled as it too is a Mendelian disease-causing gene, as will be discussed below. The structures were again generated by homology modelling to the archetype ELOVL7. When the four proteins were superimposed for structure comparison, we found that the conformation of Loop 2 overlapped precisely in all four ELOVLs, whereas the conformation of Loop 6 was clearly elongase-specific (Fig. 5e). This also suggests that the length of the conserved disulphide connecting Loop 2 and Loop 6 is also elongase-specific and likely contributes to the substrate specificity of each ELOVL, seeing as the substrate binding tunnel is formed by two ‘domains’ of three helices: one unit is formed by TM2–4 (containing LOOP 2), the other by TM5–7 (containing Loop 6) (Nie et al. 2021). Therefore, mutations that lead to modifications of the disulphide length may impact on protein conformation and function, and lead to disease.

### SCA38 p.G230V and SCA34 p.W246G are positional equivalent missense variants

Alongside *ELOVL5*, *ELOVL4* is currently the only other elongase involved in an autosomal dominant cerebellar ataxia: SCA34. The ataxic phenotype associated with *ELOVL4* is more complex as five point mutations have been identified, leading to three distinct clinical outcomes: one variant (p.W246G in Loop 6) causes a pure cerebellar ataxia; three variants (p.L168F in TM4, p.Q180P in Loop 4 and p.T233M TM6) cause ataxia with the skin disorder erythrokeratodermia and one variant (p.I171T in TM4) leads to ataxia, erythrokeratodermia and retinitis (Cadieux-Dion et al. 2014; Giroux and Barbeau 1972; Ozaki et al. 2015). Other variants cause an autosomal dominant retinal disease, Stargardt Disease 3 (STGD3; MIM #600110), a form of macular dystrophy (Bernstein et al. 2001; Maugeri et al. 2004; Zhang et al. 2001). The tissue distribution of the *ELOVL4-*associated phenotypes reflects precisely the high expression of *ELOVL4* in skin, cerebellum and photoreceptor cells of the retina. In addition, *Elovl4*-deficient mice die soon after birth because of deleterious alterations in the skin permeability barrier (Li et al. 2007).

**Fig. 6:**
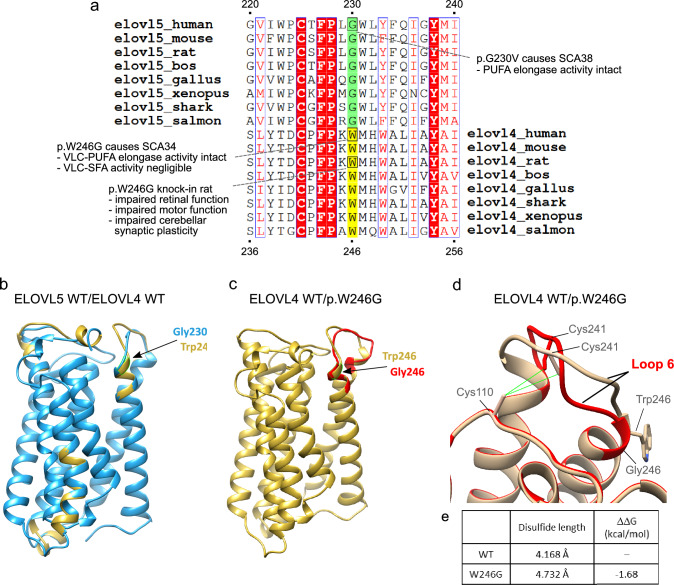
Comparative analysis of ELOVL5 and ELOVL4 proteins showing G230V and W246G are positional-equivalent mutations. **a** Relevant section of the MSA of vertebrate ELOVL5 orthologs and its second paralog ELOVL4, highlighting the Trp (W) residue that aligns with G230 of ELOVL5. The full MSA is available in the Supporting Information. **b** Superposition of ELOVL5 (blue) and ELOVL4 (gold) wild-type proteins highlighting overlapping position of ELOVL5 G230 and ELOVL4 W246. **c** Overlap of ELOVL4 wild-type (gold) and p.W246G (red) protein structures showing their discordant Loop 6 structure. **d** Enlargement of the Loop 2/Loop 6 intramolecular bond, showing how the disulphide bond changes in wild-type (gold) and p.W246 (red) ELOVL4 proteins. **e** Table illustrates the impact of the W246G substitution on disulphide bond length and protein stability. The length of the bonds were calculated and visualized in UCSF Chimera. The PDB files of wild-type and p.W246G were uploaded in the PDBsum home page (http://www.ebi.ac.uk/thornton-srv/databases/pdbsum/) to generate protein structural details including disulphide bond conformation. The program could only calculate the conformation of the conserved disulphide bond in wild-type ELOVL4 and that the right-handed spiral conformation is lost in p.W246G (not shown). The change in protein stability shown here was calculated with SAAFEC-SEQ, the negative free energy variation indicating loss of protein stability

Here we focused on the ELOVL4 p.W246G variant as this is associated with a pure cerebellar ataxia (SCA34) and was previously noted to be located close to ELOVL5 p.G230V in SCA38 (Ozaki et al. 2015). ELOVL4 is the next closest paralog to ELOVL5 (41% aa identity; 54% aa similarity) after ELOVL2. The multiple sequence alignment of ELOVL5 and ELOVL4 orthologous proteins shows that ELOVL4 W246 is ortholog-conserved and in fact occupies the same column of the alignment as G230 in ELOVL5 (Fig. 6a; Suppl. Tables 8–10). Thus, like ELOVL5 G230, ELOVL4 W246 is also highly constrained and likely to be essential for structure/function. Unlike G230 in ELOVL5 which cannot establish specific protein-stabilizing interactions, W246 likely forms pi-stacking interactions with other close (< 4.5 Å) hydrophobic residues which often stabilize protein structure, further impacting the substitution of W246 with G246.

The positional correspondence between G230 in ELOVL5 and W246 in ELOVL4 was confirmed by superimposing the wild-type ELOVL5 and ELOVL4 structures (Fig. 6b), showing that G230 and W246 overlapped precisely, indicating that p.G230V and p.W246G are indeed position-equivalent missense variants. By superimposing the structures of wild-type and p.W246G ELOVL4, we observed a clear shift in the position of Loop 6 in the variant protein, while Loop 2 remained perfectly superimposed (Fig. 6c, d). The length of the conserved disulphide bond length was increased in p.W246G (with the caveat that 3.0 Å is taken as the cutoff for disulphides in the PDB database) and protein stability was predicted to be strongly decreased (Fig. 6e). These comparisons further suggest that the conformation of Loop 6 is modified by p.W246G.

## Discussion

### “Neurodegenerative diseases stand between us and the hope of successful aging” (Kerdiles and Calon 2017)

SCA38 is an extremely rare disease, with 21 patients carrying the ELOVL5 p.G230V variant recorded to date (Brusco et al. 2019; Di Gregorio et al. 2014). Other variants in ELOVL5 have been identified, such as p.Leu72Val in a French family (Di Gregorio et al. 2014) which has not been further validated, and p.Tyr260Cys (Gazulla et al. 2020) which, however, is classified as benign and is found in healthy adults (https://varsome.com) (Kopanos et al. 2019). Notwithstanding its rarity, SCA38 adds to the enormous burden of neurodegenerative disorders (NDDs), a leading cause of morbidity and death, with enormous social and economic costs (Kovacs 2019; Peden and Ironside 2012). As many of the NDDs are currently intractable, understanding their molecular origins is a major challenge and a pre-requisite for treatment development (Ciechanover and Kwon 2015; Mallucci et al. 2020).

The scope of this work was to further our understanding of how the ELOVL5-G230V pathogenic protein leads to SCA38. To this end, we adopted a two-pronged experimental approach, in vitro functional and computational, whose results suggest that more than one mechanism of Mendelian dominance is involved in SCA38 pathogenesis.

The loss-of-function (LoF) hypothesis that p.G230V had lost enzymatic activity was dismissed as the biochemical assay showed that the variant protein was as active as wild-type in PUFA elongation. Perhaps this result was not surprising as, at the protein level, missense variants rarely cause gross alteration of protein structure (Iqbal et al. 2020; Vihinen 2021) and G230 was not predicted to affect either the active site or substrate binding. At the cellular/organismal level, even if the variant allele lost elongase activity, we would expect the wild-type allele to be haplosufficient (Karczewski et al. 2020), bearing in mind the robustness of metabolic pathways (Matias Rodrigues and Wagner 2009) and that most enzymatic defects require recessive mutations (Veitia et al. 2018; Wilkie 1994).

However, there are other avenues to LoF, notably LoF by mislocalization whereby a pathogenic protein is not targeted to its native milieu, instead accumulating ex loco and possibly forming insoluble aggregates, leading to protein depletion and LoF (Kokubun et al. 2019; Suk and Rousseaux 2020; Yang and Hu 2016). Such a mechanism occurs in Amyotrophic Lateral Sclerosis, a progressive NDD in which loss of function by nuclear-to-cytoplasmic mislocalization of TAR DNA binding protein 43 kDa (TDP-43) plays a major causative role (Atkinson et al. 2021; Suk and Rousseaux 2020). In SCA38, there is ER-to-Golgi mislocalization, the Golgi complex becoming a sinkhole where p.G230V is trapped and non-functional (Jacquemyn et al. 2017; Logan et al. 2014). So we propose that LoF by mislocalization is one pathogenetic mechanism.

Chronic accumulation of p.G230V within the Golgi complex will be detrimental, especially in post-mitotic cells such as neurons. Indeed, the Golgi complex is vital for neuronal development and maintenance, its vesicular trafficking regulating axon and dendritic growth (Ori-McKenney et al. 2012), and Golgi complex dysfunction is frequently observed in NDDs (Liu et al. 2021; Machamer 2015). In turn, disruption of the Golgi complex in the brain (e.g., by targeted deletion of GM130 in mouse) led to the phenotypic triad also observed in SCAs, i.e., Purkinje cell loss, cerebellar atrophy and ataxia (Liu et al. 2017). In addition, p.G230V accumulation may trigger the Golgi-associated protein quality control mechanisms (Briant et al. 2017), including the endosome and Golgi-associated degradation pathway (EGAD) (Schmidt et al. 2019). The EGAD substrates are known to include ER-resident membrane proteins involved in lipid biosynthesis, and play a role in regulating sphingolipid metabolism (Schmidt et al. 2019). Hence, toxic gain-of-function (GoF) by Golgi accumulation and dysfunction is proposed to be a second pathogenetic mechanism.

A clue to potential mechanism(s) driving p.G230V entrapment comes from the human binary interactome map by Luck et al*.* (2020). An hypothesis-free approach based experimentally on yeast two-hybrid assays, the interactome map revealed unknown links between proteins not previously known to co-localize, including the interaction between ELOVL5 and GOSR2/membrin, a *cis*-Golgi protein involved in forming the SNARE (soluble *N*-ethylmaleimide-sensitive factor attachment protein receptor) complex that fuses opposing lipid bilayers in vesicle fusion, thus playing a critical role in ER-to-Golgi trafficking (Lowe et al. 1997; Praschberger et al. 2017). Note that missense mutations in GOSR2 lead to a neurological phenotype of progressive epilepsy and ataxia (Praschberger et al. 2017). The physiological significance of ELOVL5 binding to GOSR2 is currently unknown and warrants further studies, including the effect of the G230V substitution on binding. Interestingly, the interactome map also reports that ELOVL4, but none of the other ELOVLs, is also a GOSR2 binding partner (Luck et al. 2020).

Proteotoxicity is one of the most frequently shared features of NDDs, including many of the SCAs (Klockgether et al. 2019; Matus et al. 2013; Scheper and Hoozemans 2015; Schroder and Kaufman 2005; Soto and Pritzkow 2018). Our previous (Di Gregorio et al. 2014) and current functional assays suggest that p.G230V undergoes proteasomal degradation and activates the UPR. Although these overexpression-based studies are exaggerated and accelerated versions of what is occurring in vivo*,* nonetheless they consistently showed a more deleterious effect of p.G230V expression with respect to wild-type, unlike the enzymatic assay that couldn’t distinguish between the two. The observation, by structural analysis, that the conserved disulphide bond is altered and likely de-stabilized in p.G230V provides an explanation for protein misfolding, with ER stress triggering numerous downstream events (UPR, ER-associated degradation, ER-phagy) (Yang et al. 2021). Hence, a toxic gain of function by ER stress-induced proteotoxicity is proposed as the third pathogenic mechanism, and the best supported mechanism according to our current data.

A potential fourth pathogenic mechanism, represented by a dominant negative effect, remains speculative, although supported by the fact that the human ELOVLs have been characterized biochemically as homo- and heterodimers (Okuda et al. 2010) and the ELOVL7 crystal structure is represented as an antiparallel dimer (Nie et al. 2021). It is a fact that the heterozygous missense p.G230V variant causes disease, whereas the heterozygous loss of *Elovl5* in mice does not appear to (Hoxha et al. 2017), indicating that ELOVL5 is haplosufficient and the effects of a single p.G230V missense change are more severe than having one null allele (Veitia 2007). The eventual phenotypic convergence between SCA38 individuals and *Elovl5* KO mice, both of which develop late-onset ataxia and hyposmia (Hoxha et al. 2017), suggests that in SCA38 there must be < 50% functional ELOVL5, compatible with a dominant negative effect. It is also worth remembering that the *Elovl5* KO mouse phenotype is obtained not just by deletion of the genes but must be forced by a diet lacking the Elovl5 downstream PUFAs (Hoxha et al. 2017).

Golgi entrapment of p.G230V could lead to depletion of ELOVL5 functional enzyme in the ER and to decreased levels of DHA and AA. We previously observed decreased levels of DHA and AA in SCA38 sera but this observation was limited to an analysis of two patients (Di Gregorio et al. 2014). As ELOVL5 expression is regulated through a negative feedback loop, reduced DHA and AA will trigger expression of the transcription factor sterol regulatory element-binding protein (SREBP) which induces ELOVL5 expression (Jacquemyn et al. 2017; Moon et al. 2001, 2009), creating a vicious circle if the Golgi does indeed behave as a sinkhole for at least half of the ELOVL5 produced by SCA38 cells (more if wild-type/p.G230V heterodimers are also mistrafficked). If we consider age-dependent proteotoxicity of mutant ELOVL5 to be the underlying cause of SCA38, a logical question to ask is why does oral administration of DHA stabilize disease manifestations in SCA38 patients? As demonstrated by Shikama et al*.* (2015), expression of *ELOVL5* is activated by the transcription factor SREBP-1 (sterol regulatory element-binding protein 1) which is in turn negatively regulated by the presence of PUFAs. Thus, one explanation would be that dietary DHA reduces expression of wild-type and mutant ELOVL5 alleles, preventing the continual increase of the unfolded protein load, alongside supplying a necessary factor that is mostly derived from non-brain sources.

The final part of our study creates a convergent framework for the elongase-mediated spinocerebellar ataxias SCA38 (by ELOVL5 p.G230V) and SCA34 (by ELOVL4 p.W246G), demonstrating by direct structural comparison that ELOVL5 G230V and ELOVL4 W246G are position-equivalent pathogenic missense variants. Prior to this study, the only connections that could be established between SCA38 by p.G230V and SCA34 by p.W246G were that the causative genes were both ER-expressed fatty acid elongases and the variant residues seemed close. Otherwise, ELOVL4 and ELOVL5 have quite discordant biological features, such as tissue distribution and enzymatic products (Table 1). The phenotype of the knockout mice are also very different: homozygous loss of *Elovl4* is severe, causing perinatal lethality with defective epidermal permeability barrier (Li et al. 2007). In contrast, *Elovl5*-deficient mice are born healthy and fertile but develop a SCA38-like phenotype with progressive ataxia, hyposmia and Purkinje cell loss (Hoxha et al. 2017). The pathological features of the variant proteins also appear to be quite distinct: (1) p.G230V is mislocalized to the Golgi unlike p.W246G which is reported as cytoplasmatic (Gyening et al. 2023) with no indication of mislocalization; (2) the mutation does not disrupt the enzymatic activity of p.G230V whereas that of p.W246G is modified: PUFA elongation is normal whereas saturated fatty acid elongation is reduced, but mostly affecting the retina (Agbaga et al. 2020; Gyening et al. 2023). Thus the identification of p.G230V and p.W246G as positional equivalent missense variants that modify the protein conformation is a first point of contact between their respective pathogenic pathways. Indeed, in view of the functional dissimilarities between ELOVL5 p.G230V and ELOVL4 p.W246G, we believe protein misfolding and proteotoxic ER stress induction in Purkinje cells is the prime mover in SCA38 and SCA34 pathogenesis.

**Table 1:** Compared features of ELOVL5/p.G230V/SCA38 and ELOVL4/p.W246G/SCA34

Disease	SCA38	SCA34
Gene, location	*ELOVL5*, 6p12.1	*ELOVL4*, 6q14.1
Inheritance	Autosomal dominant	Autosomal dominant
Ethnicity	Italian	Japanese
Brain MRI	Cerebellar atrophy	Cerebellar and pontine atrophy
Phenotype	Gait, speech and limb ataxia, hyposmia	Gait, speech and limb ataxia

Wild-type protein	ELOVL5	ELOVL4
Function	Fatty acid elongase	Fatty acid elongase
Products	C20–C22 PUFAs	C28–C38 VLC-PUFAs and VLC-SFAs
Cellular localization	ER	ER
Tissue distribution	Ubiquitous	Skin, retina, cerebellum, testis
Phenotype knockout mouse	SCA38-like disease	Neonatal lethal

Pathogenic protein	p.G230V	p.W246G
Enzymatic activity	Normal	Altered
Localization	Altered	Normal
Conformational change predicted	Yes	Yes

Compelling evidence for the relevance of the Loop 2–Loop 6 intramolecular disulphide bond comes from comparison of the native ELOVL protein structures that show complete overlap in Loop 2 conformation but ELOVL-specific conformation of Loop 6, at least in the ELOVLs examined here, i.e., ELOVL2, 4, 5 and 7. This suggests that each ELOVL protein requires the conserved disulphide to assume the correct native conformation, each differing from one ELOVL to another probably also to help define their substrate specificity. A guiding example of the role of disulphides in heptahelical TM proteins is one of the G protein-coupled receptors: gonadotropin-releasing hormone receptor type I (GnRHR). Human GnRHR has an intramolecular disulphide bond, connecting the N-terminal tract with Loop 4, whose integrity is required for correct intracellular trafficking to reach the plasma membrane (Conn and Ulloa-Aguirre 2011; Tao and Conn 2018).

Ultimately, ELOLV5 p.G230V and ELOVL4 p.W246G exacerbate the ‘Herculean task’ (Pfenninger 2009) facing Purkinje cerebellar neurons in maintaining the extensive lipid membrane network both within the cell and on the Purkinje cell surface, the beauty of these cells coming at the price of their vulnerability. Similarly, with bodies calibrated for reproductive fitness and not for growing old, the cerebellum’s capacity to neutralize proteotoxicity decreases with ageing (Balchin et al. 2016; Kokubun et al. 2019), and is exacerbated by mutant proteins such as ELOVL5 p.G230V and ELOVL4 p.W246G, reaching a threshold beyond which accumulation causes damage to vulnerable Purkinje cells.

Like most NDDs, the SCAs are characterized by a lack of available disease-modifying treatments (Ashizawa et al. 2018; Bushart et al. 2016; Mallucci et al. 2020), although new therapeutic strategies are on the horizon (Ashizawa et al. 2018; Ciechanover and Kwon 2015; Hachem et al. 2020; Hartl 2017; Mallucci et al. 2020; Tao and Conn 2018). Elucidating the underlying pathogenic mechanisms of NDDs is crucial for defining new therapeutic approaches, and our study underlines how challenging this is, with several pathogenetic mechanisms contributing to causing autosomal dominant SCA38.

## Supplementary Information

Below is the link to the electronic supplementary material.

Supplementary file1 (PDF 740 KB)

## Data Availability

The datasets generated during and/or analysed during the current study are available from the corresponding author on reasonable request.
