# Supplementary material for: Spinocerebellar ataxia 38: structure–function analysis shows ELOVL5 G230V is proteotoxic, conformationally altered and a mutational hotspot
Source: Hum Genet. 2023 May 18;142(8):1055–76. doi: 10.1007/s00439-023-02572-y (PMC10449689; doi:10.1007/s00439-023-02572-y)
Supplement: Supplementary file 1 — Supplementary file1 (PDF 740 KB) [file 439_2023_2572_MOESM1_ESM.pdf]

Spinocerebellar ataxia 38: structure-function analysis shows ELOVL5 G230V is proteotoxic, conformationally altered and a mutational hotspot

Ferrero et al.

Supplementary information

Enza Ferrero[1], Eleonora Di Gregorio[2], Marta Ferrero[3], Erika Ortolan[1], Young-Ah Moon[4], Antonella Di Campli[5,6], Lisa Pavinato[1], Cecilia Mancini[1,7], Debasmita Tripathy[8], Marta Manes[9], Eriola Hoxha[10], Chiara Costanzi[11], Elisa Pozzi[1], Matteo Rossi Sebastiano[12], Nico Mitro[13], Filippo Tempia[10], Donatella Caruso[13], Barbara Borroni[8], Manuela Basso[9], Michele Sallese[14], Alfredo Brusco[1,2]

1. Department of Medical Sciences, University of Torino, Torino, Italy
2. Unit of Medical Genetics, Città della Salute e della Scienza Hospital, Torino, Italy
3. Experimental Zooprophylactic Institute of Piedmont, Liguria & Aosta Valley, Torino, Italy
4. Department of Molecular Medicine, Inha University College of Medicine, Incheon, South Korea
5. Institute of Protein Biochemistry, Italian National Research Council, Napoli, Italy
6. Department of Innovative Technologies in Medicine and Dentistry, G. d'Annunzio University of Chieti-Pescara, Chieti, Italy
7. Genetics and Rare Diseases Research Division, Bambino Gesù Children's Hospital, Rome, Italy
8. Department of Clinical and Experimental Sciences, University of Brescia, Brescia, Italy
9. Department of Cellular, Computational and Integrative Biology, University of Trento, Trento, Italy Neuroscience Institute Cavalieri Ottolenghi, Orbassano and Department of Neuroscience, University of Torino, Torino, Italy
10. Department of Neurology, Cremona Hospital, Cremona, Italy
11. Department of Molecular Biotechnology and Health Sciences, University of Torino, Torino, Italy.
12. Department of Pharmacological and Biomolecular Sciences, University of Milan, Milan, Italy
13. Centre for Advanced Studies and Technology, G. d'Annunzio University of Chieti-Pescara, Chieti, Italy

The corresponding author is: Professor Alfredo Brusco, University of Torino, Department of Medical Sciences, Via Santena 19, 10126, Torino, Italy. Fax: +39 011 236 5926. Email: alfredo.brusco@unito.it

Spinocerebellar ataxia 38: structure-function analysis shows ELOVL5 G230V is proteotoxic, conformationally altered and a mutational hotspot

Ferrero et al.

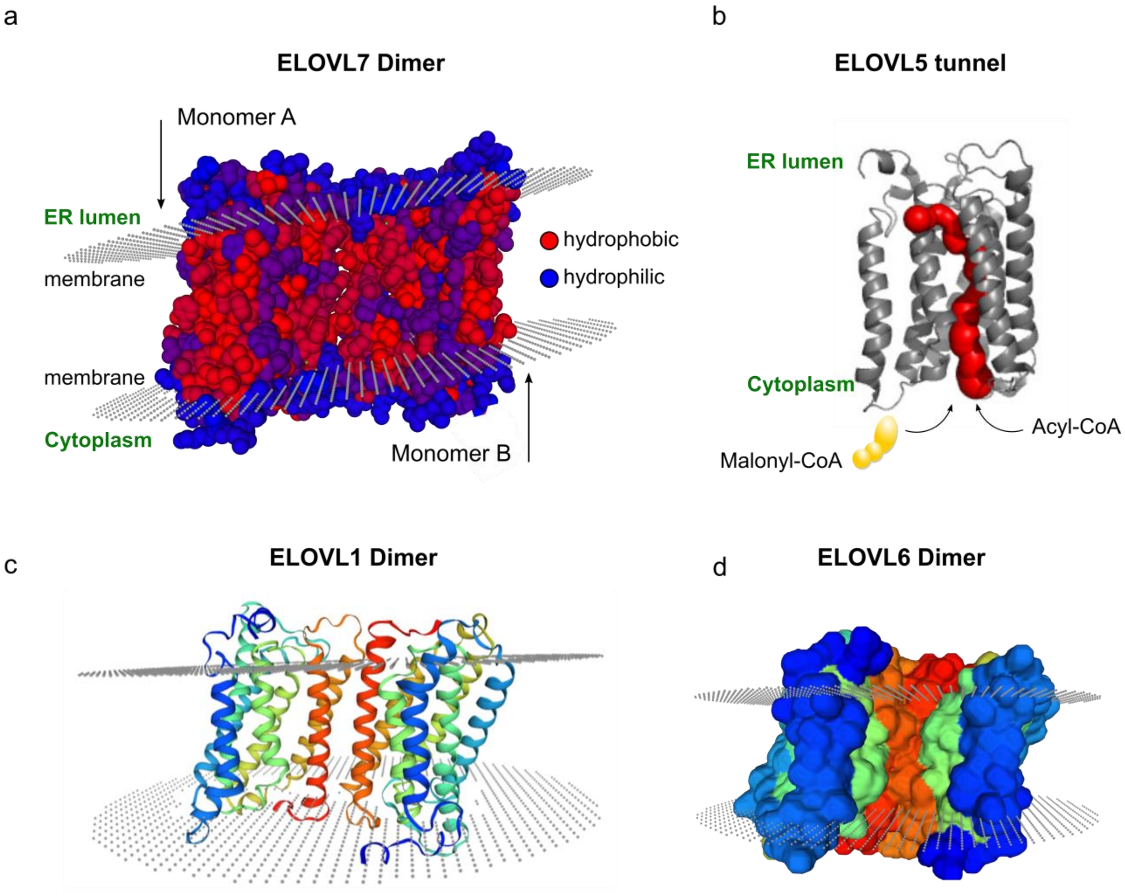

Supplementary Figure 1

Suppl. Fig. 1. Dimeric structure of human elongases. To date, three of the human elongases (ELOVL7, ELOVL1 and ELOVL6) are represented as homodimers in the Swiss-Model website (https://swissmodel.expasy.org/). (a) ELOVL7 homodimer (UniProtKB accession no. A1L3X0) is shown. Monomer A and B are represented in antiparallel array; hydrophobic or hydrophilic nature of the resides are indicated in this space-filling model. (b) Pore analysis of human ELOVL5 3D protein in PDBsum showing substrate-binding tunnel. The catalytic mechanism is a ping-pong type, two-step mechanism as described by Nie et al. (c) Cartoon model of ELOVL1 dimer (Q9BW60) with rainbow color scheme. (d) Surface model of ELOVL6 dimer (Q9H5J4) with rainbow color scheme.

Spinocerebellar ataxia 38: structure-function analysis shows ELOVL5 G230V is proteotoxic, conformationally altered and a mutational hotspot

Ferrero et al.

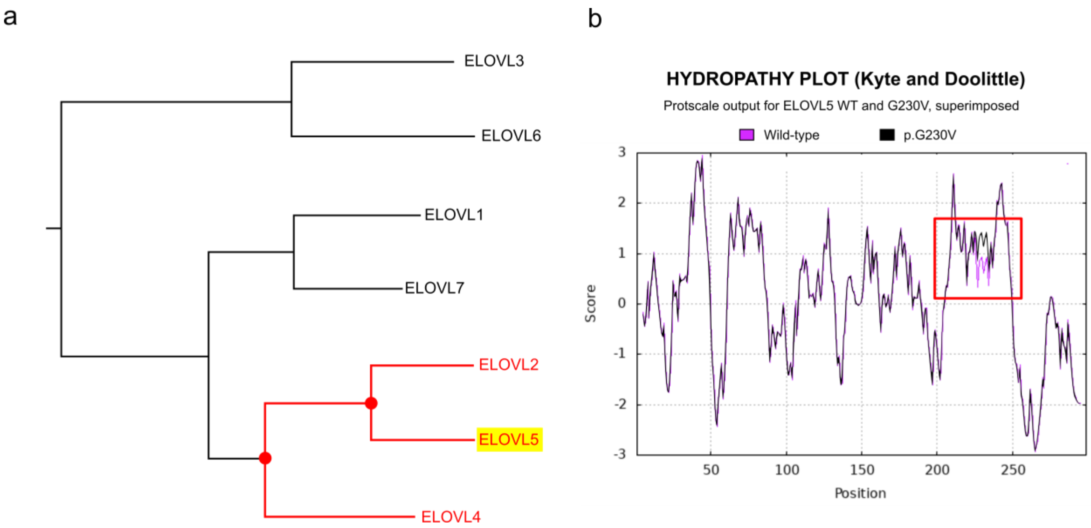

Supplementary Figure 2

Suppl. Fig. 2. (a) Neighbour-joining tree illustrating the phylogenetic relationship between the seven human ELOVL proteins. ELOVL2 and ELOVL4 proteins are the most closely related to ELOVL5, as highlighted by the red lines of the cladogram. The duplication nodes are indicated (●). Tree construction is detailed in the Materials and Methods. (b) Kyte-Doolittle hydropathy plot of wild-type (mauve line) and p.G230V (black line) ELOVL5 proteins showing increased hydrophobicity due to the G230V substitution.

Spinocerebellar ataxia 38: structure-function analysis shows ELOVL5 G230V is proteotoxic, conformationally altered and a mutational hotspot

Ferrero et al.

Supplementary Figure 3

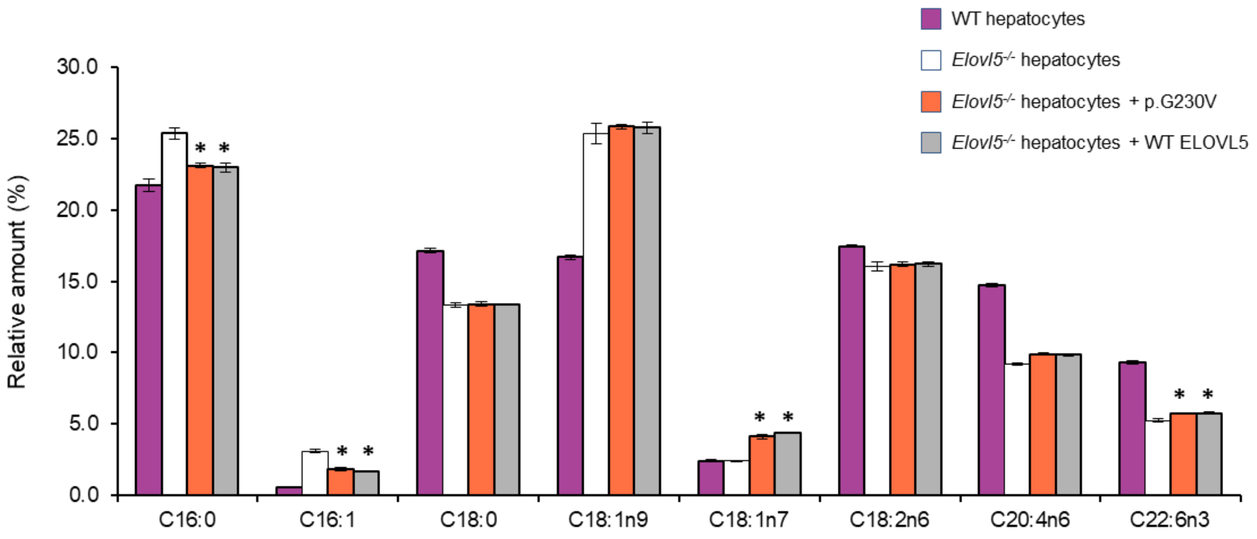

Suppl. Fig. 3. Similar lipid profiles in hepatocytes from *Elovl5*$^{-/-}$ mice transfected with wild-type or variant ELOVL5. On day 0, primary hepatocytes were isolated from wild-type and *Elovl5*$^{-/-}$ mice. Cells were attached for 4 h and then infected with adenovirus to express either wild-type ELOVL5 or ELOVL5 p.G230V. ELOVL5 substrate fatty acids were not added to the medium. On day 2, lipids were extracted from the cells and whole cell lysates were prepared for protein expression and lipid composition as described in Moon et al., 2009. * indicates $p<0.05$ by Student t-test when comparing *Elovl5*$^{-/-}$ hepatocytes and *Elovl5*$^{-/-}$ hepatocytes + p.G230V or *Elovl5*$^{-/-}$ hep+WT ELOVL5. No difference was detected when comparing *Elovl5*$^{-/-}$ hepatocytes + p.G230V and *Elovl5*$^{-/-}$ hepatocytes + WT ELOVL5.

Spinocerebellar ataxia 38: structure-function analysis shows ELOVL5 G230V is proteotoxic, conformationally altered and a mutational hotspot

Ferrero et al.

Supplementary Table 1 Multiple sequence alignment of human ELOVL proteins

```
ELOVL1          ----MEA-------------------VVNLYQEVMKHADPRIQGYPLMGSPLLMTSILLTY
ELOVL7          ----MAF------------SDLTSRTVHLYDNWIKDADPRVEDWLLMSSPLPQTILLGFY
ELOVL2          ----MEHL-----------KAFDDEINAFLDNMFGPRDSRVRGWFMLDSYLPTFFLTVMY
ELOVL5          ----MEH--------------FDASLSTYFKALLGPRDTRVKGWFLLDNYIPTFICSVIY
ELOVL4          ----MGLLDSEPGSVLNVVSTALNDTVEFYRWTWSIADKRVENWPLMQSPWPTLSISTLY
ELOVL3          MVTAMNV------------SHEVNQLFQPYNFELS---KDMRPF-FEEYWATSFPIALIY
ELOVL6          ----MNM------------SVLT---LQEYEFEKQFNENEAIQW-MQENWKKSFLFSALY
                    *                                      : :             *

ELOVL1          VYFVLSLGPRIMANRKPFQLRGFMIVYNFSLVALSLY--------IVYEFLMSGWLSTYT
ELOVL7          VYFVTSLGPKLMENRKPFELKKAMITYNFFIVLFSVY--------MCYEFVMSGWGIGYS
ELOVL2          LLSIW-LGNKYMKNRPALSLRGILTLYNLGITLLSAY--------MLAELILSTWEGGYN
ELOVL5          LLIVW-LGPKYMRNKQPFSCRGILVVYNLGLTLLSLY--------MFCELVTGVWEGKYN
ELOVL4          LLFVW-LGPKWMKDREPFQMRLVLIIYNFGMVLLNLF--------IFRELFMGSYNAGYS
ELOVL3          LVLIA-VGQNYMKERKGFNLQGPLILWSFCLAIFSILGAVRMWGIMGTVLLTGGLKQTV-
ELOVL6          AAFIF-GGRHLMNKRAKFELRKPLVLWSLTLAVFSIFGALRTGAYMVYILMTKGLKQSV-
                   :   * . * .:  :. :  :  :.: :. :.          :   :.

ELOVL1          WRCDPVDYSNSPEALRMVRVAWLFLFSKFIELMDTVIFILRKKDGQVTFLHVFHHSVLPW
ELOVL7          FRCDIVDYSRSPTALRMARTCWLYYFSKFIELLDTIFFVLRKKNSQVTFLHVFHHTIMPW
ELOVL2          LQCQDLTSAGEA-DIRVAKVLWWYYFSKSVEFLDTIFFVLRKKTSQITFLHVYHHASMFN
ELOVL5          FFCQGTRTAGES-DMKIIRVLWWYYFSKLIEFMDTFFFILRKNNHQITVLHVYHHASMLN
ELOVL4          YICQSVDYSNNVHEVRIAAALWWYFVSKGVEYLDTVFFILRKKNNQVSFLHVYHHCTMFT
ELOVL3          --CFINFIDNST----VKFWSWVFLLSKVIELGDTAFIILRKR--PLIFIHWYHHSTVLV
ELOVL6          --CDQGFYNGPV----SKFWAYAFVLSKAPELGDTIFIILRKQ--KLIFLHWYHHITVLL
                  *                  : : .**  *  ** :::***.   : .:* :**  :

ELOVL1          SWWWGVKIAPGGMGSFHAMINSSVHVIMYLYYGLSAFGPVAQPYLWWKKHMTAIQLIQFV
ELOVL7          TWWFGVKFAAGGLGTFHALLNTAVHVVMYSYYGLSALGPAYQKYLWWKKYLTSLQLVQFV
ELOVL2          IWWCVLNWIPCGQSFFGPTLNSFIHILMYSYYGLSVF-PSMHKYLWWKKYLTQAQLVQFV
ELOVL5          IWWFVMNWVPCGHSYFGATLNSFIHVLMYSYYGLSSV-PSMRPYLWWKKYITQGQLLQFV
ELOVL4          LWWIGIKWVAGGQAFFGAQLNSFIHVIMYSYYGLTAFGPWIQKYLWWKRYLTMLQLIQFH
ELOVL3          YTSFGYKNKVPAGGWF-VTMNFGVHAIMYTYYTLKAANVKPPKML--PMLITSLQILQMF
ELOVL6          YSWYSYKDMVAGGGWF-MTMNYGVHAVMYSYYALRAAGFRVSRKF--AMFITLSQITQML
                      :    . . *   :*  :* :** ** *          :     :*  *: *:

ELOVL1          LVSLHISQYYFM---SSCNYQYPVIIHLIWMYGTIFFMLFSNFWYHSYTK---GKRLP--
ELOVL7          IVAIHISQFFFM---EDCKYQFPVFACIIMSYSFMFLLLFLHFWYRAYTK---GQRLP--
ELOVL2          LTITHTMSAVVK----PCGFPFGCLI-FQSSYMLTLVILFLNFYVQTY------RKKPMK
ELOVL5          LTIIQTSCGVIW----PCTFPLGWLY-FQIGYMISLIALFTNFYIQTYNKKGASRRKDHL
ELOVL4          VTIGHTALSLYT----DCPFPKWMHW-ALIAYAISFIFLFLNFYIRTYKE---PKKPKAG
ELOVL3          VGAIVSILTYIWRQDQGCHTTMEHLFWSFILY-MTYFILFAHFFCQTYIR---PKVKA--
ELOVL6          MGCVVNYLVFCWMQHDQCHSHFQNIFWSSLMY-LSYLVLFCHFFFEAYI----GKMRK--
                :                *             *    . ** :*: .:*      :

ELOVL1          RALQQNGAPGIA----------------------KVKAN
ELOVL7          KTVKN----GTC----------------------KNKDN
ELOVL2          KDMQE--PPAGKEVKNGFSKAYFTAANGVMNK--KAQ--
ELOVL5          KDHQN----GSMAAVNGHTNSFSPLENNVKPR--KLRKD
ELOVL4          KTAMN----GISANGVSKSEKQLMIENGKKQKNGKAKGD
ELOVL3          ----------------------------------KTKSQ
ELOVL6          ----------------------------------TTKAE
                    .:
```

Multiple sequence alignment of human ELOVL1-7 aligned by MAFFT online program. Cysteine residues that form the conserved intramolecular disulfide bond connecting Loop 2 and Loop 6 are colored in cyan. The catalytic histidine-rich active site is showed in yellow. The green column contains the ELOVL5 G230 residue that is substituted to V230 in SCA38. The output order reflects the results of the alignment. Sequence accession numbers are: ELOVL1 (Q9BW60.1)

279aa; ELOVL2 (Q9NXB9.2) 296aa; ELOVL3 (Q9HB03.2) 270aa; ELOVL4 (Q9GZR5.1) 314aa; ELOVL5 (Q9NYP7.1) 299aa; ELOVL6 (Q9H5J4.1) 265aa; ELOVL7 (A1L3X0.1) 281aa.

Supplementary Table 2 Percent of amino acid sequence identity and similarity between the human ELOVL proteins

Amino Acid Identity (%)

**ELOVL1**	100						
**ELOVL2**	32.61	100					
**ELOVL3**	25.55	22.59	100				
**ELOVL4**	41.21	41.21	25.18	100			
**ELOVL5**	35.12	57.09	24.44	41.13	100		
**ELOVL6**	23.77	21.88	45.66	23.77	22.26	100	
**ELOVL7**	57.34	38.07	24.44	44.48	36.29	26.79	100
	**ELOVL1**	**ELOVL2**	**ELOVL3**	**ELOVL4**	**ELOVL5**	**ELOVL6**	**ELOVL7**

Amino Acid Similarity (%)

**ELOVL1**	100						
**ELOVL2**	45.16	100					
**ELOVL3**	36.29	35.18	100				
**ELOVL4**	51.61	54.05	38.14	100			
**ELOVL5**	47.31	68.24	39.25	53.84	100		
**ELOVL6**	35.09	34.71	56.22	35.84	36.98	100	
**ELOVL7**	69.53	45.55	36.29	54.44	45.19	38.49	100
	**ELOVL1**	**ELOVL2**	**ELOVL3**	**ELOVL4**	**ELOVL5**	**ELOVL6**	**ELOVL7**

The percent amino acid identity and similarity were calculated using the SIAS programme as detailed in the methods.

Spinocerebellar ataxia 38: structure-function analysis shows ELOVL5 G230V is proteotoxic, conformationally altered and a mutational hotspot

Ferrero et al.

Supplementary Table 3 Links or addresses of webservers/databases used in this study

Ataxia website:	https://neuromuscular.wustl.edu/ataxia/domatax.html
NCBI PDB structure database:	https://www.ncbi.nlm.nih.gov/structure/
NCBI Protein sequence database	https://www.ncbi.nlm.nih.gov/protein/
Mafft protein sequence alignment and tree	https://mafft.cbrc.jp/alignment/server/
SIAS percent aa sequence identity & similarity	http://imed.med.ucm.es/Tools/sias.html
Pfam protein family database	https://www.ebi.ac.uk/interpro/
Protter proteoform visualization	https://wlab.ethz.ch/protter/start/
Phyre2 protein modelling	http://www.sbg.bio.ic.ac.uk/~phyre2/html/page.cgi?id=index
UCSF Chimera protein modelling	https://www.cgl.ucsf.edu/chimera/
I-Mutant protein stability predictor	https://folding.biofold.org/i-mutant/i-mutant2.0.html
MAESTRO protein stability predictor	https://biwww.che.sbg.ac.at/
INPS-MD prediction server	https://inpsmd.biocomp.unibo.it/welcome/default/index
SAAFEC-SEQ stability predictor	http://compbio.clemson.edu/SAAFEC-SEQ/
I-Tasser protein modelling	https://zhanggroup.org/I-TASSER/
Swiss Model protein modelling	https://swissmodel.expasy.org/
AlphaFold Protein Structure Database	https://alphafold.ebi.ac.uk/
Kyte & Doolittle Hydropathy plot	https://web.expasy.org/protscale/
Human genomic variant search engine	https://varsome.com
PDBsum protein structure database	http://www.ebi.ac.uk/thornton-srv/databases/pdbsum/

Spinocerebellar ataxia 38: structure-function analysis shows ELOVL5 G230V is proteotoxic, conformationally altered and a mutational hotspot

Ferrero et al.

Supplementary Table 4 ELOVL5 Protein sequences used to generate the MSA

	**Accession no.**	**Species**	**Length** (Amino acids)
1	Q9NYP7.1	*Homo sapiens* (Human)	299
2	NP_599016.2	*Mus musculus* (Mouse)	299
3	NP_599209.1	*Rattus norvegicus* (Rat)	299
4	NP_001040062.1	*Bos taurus* (Bovine)	299
5	NP_001186126.1	*Gallus gallus* (Chicken)	295
6	NP_001011248.1	*Xenopus tropicalis* (Frog)	295
7	NP_001306343.1	*Macaca fascicularis* (Macaque)	299
8	NP_001130024.1	*Salmo salar* (Salmon)	294
9	NP_001290303.1	*Larimichthys crocea* (Croaker)	294
10	NP_001297348.1	*Anas platyrhynchos* (Duck)	295
11	XP_036724720.1	*Balaenoptera musculus* (Blue whale)	299
12	XP_036269542.1	*Pipistrellus kuhlii* (Bat)	299
13	XP_007892243.1	*Callorhinchus milii* (Elephant shark)	290
14	XP_020389096.1	*Rhincodon typus* (Whale shark)	293
15	AFR36912.1	*Esox lucius* (Pike)	295
16	NP_956747.1	*Danio rerio* (Zebrafish)	291
17	JAV42152.1	*Castor canadensis* (Beaver)	299
18	BBL33563.1	*Hypoclinemus mentalis* (Sole)	294
19	XP_037750142.1	*Chelonia mydas* (Turtle)	295
20	XP_037638316.1	*Sebastes umbrosus* (Rockfish)	294
21	XP_037375770.1	*Talpa occidentalis* (Mole)	299
22	XP_037248229.1	*Falco rusticolus* (Falcon)	295
23	XP_037015462.1	*Artibeus jamaicensis* (Fruit bat)	299
24	XP_017512380.2	*Manis javanica* (Pangolin)	299
25	XP_036772263.1	*Manis pentadactyla* (Chinese pangolin)	299
26	KAF7246807.1	*Varanus komodoensis* (Komodo dragon)	295
27	XP_027457290.1	*Zalophus californianus* (Sea lion)	298
28	XP_035256991.1	*Anguilla anguilla* (Eel)	296
29	XP_033874880.1	*Acipenser ruthenus* (Sturgeon)	294
30	XP_019805339.1	*Tursiops truncatus* (dolphin)	295

Spinocerebellar ataxia 38: structure-function analysis shows ELOVL5 G230V is proteotoxic, conformationally altered and a mutational hotspot

Ferrero et al.

Supplementary Table 5 CLUSTAL format alignment by MAFFT (v7.490)of **ELOVL5 proteins**

```
HumanElovl5     --MEHFDASLSTYFKALLGPRDTRVKGWFLLDNYIPTFICSVIYLLIVWLGPKYMRNKQP
Macaca.NP_00130 --MEHFDASLSTYFKALLGPRDTRVKGWFLLDNYIPTFICSVIYLLIVWLGPKYMRNKQP
SeaLion.XP_0274 --MENFDASLSTYFKALLGPRDTRVKGWFLLDNYIPTFICSVIYLLIVWLGPKYMKNRQP
Bat.XP_03626954 --MEHFDASLSTYFKALLGPRDTRVKGWFLLDNYIPTFICSTIYLLIVWLGPKYMKNRQP
FruitBat.XP_037 --MENFDASLSTYFKAWLGPRDTRVKGWFLLDNYIPTFICSVIYLLIVWLGPKYMKNRQP
Mouse.NP_599016 --MEHFDASLSTYFKAFLGPRDTRVKGWFLLDNYIPTFVCSVIYLLIVWLGPKYMKNRQP
Rat.NP_599209.1 --MEHFDASLSTYFRALLGPRDTRVKGWFLLDNYIPTFVCSAIYLLIVWLGPKYMKNRQP
Beaver.JAV42152 --MENFDASLSTYFRAWLGPRDTRVKGWFLLDNYIPTFVCSVIYLLIVWLGPKYMKNRQP
Pangolin.XP_017 --MENFDASLSTYFKALLGPRDTRVKGWFLLDNYIPTFICSIIYLLIVWLGPKYMKNRQP
ChPangolin.XP_0 --MENFDASLSTYFKALLGPRDTRVKGWFLMDNYIPTFICSIIYLLIVWLGPKYMKNRQP
Mole.XP_0373757 --MENFDASLSTYFKALLGPRDTRVKGWFLLDNYIPTFICSVIYLLIVWLGPKYMKNRQA
Bos.NP_00104006 --MEHFDASLSTYFRAWLGPRDTRVEGWFLLDNYVPTLVCSILYLLIVWLGPKYMKTRQP
BlueWhale.XP_03 --MEHFDASLSTYFQALLGPRDLRVKGWFLLDNYIPTLLCSILYLLIVWLGPKYMKNRPP
Dolphin.XP_0198 --MEHFDASLSTYFQALLGPRDPRVKGWFLLDSYIPTLLCSILYLLIVWLGPKYMKNRPP
Gallus.NP_00118 --MERLDKTINSYLDVWLGPRDPRVKGWLLLENYTPTFIFSVLYLLIVWLGPKYMRNKQP
Duck.NP_0012973 --MERLDKTINSYLDVWLGPRDPRVKGWLLLENYTPTFIFSVLYLLIVWLGPKYMRNKQP
Falcon.XP_03724 --MEFLDKTINSYLDVWLGPRDPRVKGWLLLENYTPTFIFSVLYLLIVWLGPKYMRNKQP
Turtle.XP_03775 --MESLDQTINSYLDVWFGPRDPRVKGWLLLDNYIPTFIFSVLYLLIVWLGPKYMQNKQP
Komodo.KAF72468 --MESLDKTINSYLDVWLGPRDPRVKGWLLLTNYTPTFIFSVLYLLIVWLGPKYMQKRPP
Xenopus.NP_0010 --MEVLDKAVNGYIDHLLGPKDPRVRGWLLLDNYVPTILFTALYLFIVWRGPKYMQNRPP
Salmo.NP_001130 --MEAFNHKLNTYIDSWMGPRDERVQGWLLLDNYPPTFALTLMYLLIVWLGPKYMRHRQP
Pike.AFR36912.1 --METFNQRLNTFIDSWMGPRDERVRGWLLLDNYPPTFALTVIYLLIVWAGPKFMRHRQP
Croaker.NP_0012 --METFNHKLNTYLESWMGPRDQRVRGWLLLDNYPPTFALTVMYLVIVWMGPKYMKHRQP
Rockfish.XP_037 --METFNHRLNTYLESWMGPRDQRVRGWLLLDNYPPTFALTIMYLLIVWMGPKYMKHRQP
AmSole.BBL33563 --METFNHKLNAYIDSWLGPRDQRVRGWLLLDNYPPTFALTVMYLLIVWLGPKYMKHRQP
Eel.XP_03525699 MDMEMFNHRLNTYIDSWMGPRDQRVRGWLLLDDYPPTFALTVAYLLIVWMGPKYMKNRQP
Sturgeon.XP_033 --MEALNQATNAYIDSWMGPRDTRVRGWFLLDNFSPTVVLTLLYLFVVWIGPKYMKNKEP
Zebrafish.NP_95 --METFSHRVNSYIDSWMGPRDLRVTGWFLLDDYIPTFIFTVMYLLIVWMGPKYMKNRQA
Eshark.XP_00789 --MGFADQTLNSYFDSWFGPRDPRVEGFLLLDNYPPTFLLTLIYLFVVWAGPKYMKNKAP
WhaleShark.XP_0 --MEAVDRTLNYYFVSWFGPRDSRVEGFFLLDKYPPTFILSLLYLFVVWAGPKYMQNKAP
                  *   .   . ::   :**:* ** *::*: .: **.  :  **.:** ***:*: : .

HumanElovl5     FSCRGILVVYNLGLTLLSLYMFCELVTGVWEGKYNFFCQGTRTAGESDMKIIRVLWWYYF
Macaca.NP_00130 FSCRGILVVYNLGLTLLSLYMFCELVTGVWEGKYNFFCQGTRTAGESDMKIIRVLRWYYF
SeaLion.XP_0274 FSCRGILVLYNLGLTLLSLYMFCELVKGVWEGRYNFFCQGTRSAGEADMKIIRVLWWYYF
Bat.XP_03626954 LACRKILVVYNLGLTLLSLYMFCELVTGVWEGQYNFFCQGTRSAGEADMKIIRVLWWYYF
FruitBat.XP_037 FSCRGILVVYNLGLTLLSLYMFCELVTGVWEGKYNFFCQGTRSAGEADMKIIRVLWWYYF
Mouse.NP_599016 FSCRGILQLYNLGLTLLSLYMFYELVTGVWEGKYNFFCQGTRSAGESDMKIIRVLWWYYF
Rat.NP_599209.1 FSCRGILVVYNLGLTLLSLYMFYELVTGVWEGKYNFFCQGTRSAGESDMKVIRVLWWYYF
Beaver.JAV42152 FSCRGILVVYNLGLTLLSLYMFYELVTGVWEGKYNFFCQGTRSAGESDMKIIQVLWWYYF
Pangolin.XP_017 FSCRGILVVYNLGLTLLSLYMFYELVTGVWEGRYNFFCQGTRSSGEADVKVIRVLWWYYF
ChPangolin.XP_0 FSCRGILVVYNLGLTLLSLYMFYELVTGVWEGRYNFFCQGTRSSGEADVKVIRVLWWYYF
Mole.XP_0373757 FSCRGILVVYNLGLTLLSLYMFCELVTGVWEGKYNFFCQDTRSAGEADMKIIHVLWWYYF
Bos.NP_00104006 FSCRGILVVYNLGLTLLSLYMFCELVTGVWEGQYNFFCQGTRSGGEADMKIIRVLWWYYF
BlueWhale.XP_03 FSCRGILVVYNLGLTLLSLYMFCELVTGVWEGQYNFFCQGTRSRGEADMKIVRVLWWYYF
Dolphin.XP_0198 FSCRGILVLYNLGLTLLSLYMFCELVTGVWEGQYNFFCQGTRSGGEADMKIIRVLWWYYF
Gallus.NP_00118 FSCRGILVVYNLGLTLLSLYMFYELVTGVWEGGYNFYCQDTHSGGEADMKIIRVLWWYYF
Duck.NP_0012973 FSCRGILVVYNLGLTLLSLYMFYELVTGVWEGGYNFFCQDTHSGGEADMKIIRVLWWYYF
Falcon.XP_03724 FSCRGILVVYNLGLTLLSLYMFYELVTGVWEGGYNFFCQDTHSGGEADMKIIRVLWWYYF
Turtle.XP_03775 FSCRGILVVYNVGLTLLSLYMFYELVTGVWEGGYNFFCQDTHSGGEADMKIVRVLWWYYF
Komodo.KAF72468 FSCRGILVVYNLGLTLLSLYMFYELVTGVWEGGYNFFCQDTHSGGEADMKIIRVLWWYYF
Xenopus.NP_0010 VSCRGILVVYNLGLTLLSLYMFYELVTGVWEGGYNFFCQDTNSGGDADTKIVRVLWWYYF
Salmo.NP_001130 VSCQGLLVLYNLALTLLSFYMFYEMVSAVWQGGYNFYCQDTHSAGETDTKIINVLWWYYF
Pike.AFR36912.1 LSCRGFLVVYNLGLALLSLYMFSEMVSAVWVGGYHFYCQDTRSAGEADTRIIHVLWWYYF
Croaker.NP_0012 YSCRGLLVLYNLGLTLLSFYMFYELVTAVWHGGYNFYCQDIHSAQEVDNKIINVLWWYYF
Rockfish.XP_037 YSCRGLLVLYNLGLTLLSFYMFYELVSAVRHGGYNFYCQDTHSAQEVDNKIMNVLWWYYF
AmSole.BBL33563 YSCRGLLVVYNLGLTLLSFYMFYELVGAVWHGDYNFYCQNTHSAPEVDKKVINVLWWYYF
Eel.XP_03525699 FSCRGLLVVYNLGLTLLSLYMFCELVNGMWQGNYNFFCQDTHSAGEADTKIINVLWWYYF
Sturgeon.XP_033 FSCRGVLVVYNIGLTLLSFYMFYELVTGAWQGGYNFFCQDTHSGGEADMKIVRVLWWYYF
Zebrafish.NP_95 YSCRALLVPYNLCLTLLSLYMFYELVMSVYQGGYNFFCQNTHSGGDADNRMMNVLWWYYF
Eshark.XP_00789 VSCRGTLVVYNIGLTLLSLYMFYELLTGVWEGGYSIFCQDTHSAGKADLKIVRVLWWYYF
WhaleShark.XP_0 LSSRGILVVYNIGLTLLSLYMFCELVRGVWDGGYSIFCQNTHNAGEADLKIIRVLWWYYF
```

```
                  :.:  *  **: *:***:*** *:: .    * * ::**. ..   . * :::.** ****


HumanElovl5     SKLIEFMDTFFFILRKNNHQITVLHVYHHASMLNIWWFVMNWVPCGHSYFGATLNSFIHV
Macaca.NP_00130 SKLIEFMDTFFFILRKNNHQITVLHVYHHASMLNIWWFVMNWVPCGHSYFGATLNSFIHV
SeaLion.XP_0274 SKLIEFMDTFFFILRKNNHQITVLHVYHHASMLNIWWFVMNWVPCGHSYFGATLNSFIHV
Bat.XP_03626954 SKLIEFMDTFFFILRKNNHQITVLHVYHHASMLNIWWFVMNWVPCGHSYFGATLNSFIHV
FruitBat.XP_037 SKLIEFMDTFFFILRKNNHQVTVLHVYHHASMLNIWWFVMNWVPCGHSYFGATLNSFIHV
Mouse.NP_599016 SKLIEFMDTFFFILRKNNHQITVLHVYHHATMLNIWWFVMNWVPCGHSYFGATLNSFIHV
Rat.NP_599209.1 SKLIEFMDTFFFILRKNNHQITVLHVYHHATMLNIWWFVMNWVPCGHSYFGATLNSFIHV
Beaver.JAV42152 SKLIEFMDTFFFILRKNNHQITVLHVYHHASMLNIWWFVMNWVPCGHSYFGATLNSFIHV
Pangolin.XP_017 SKLIEFMDTFFFILRKNNHQITVLHVYHHVSMLNIWWFVMNWVPCGHSYFGATLNSFIHV
ChPangolin.XP_0 SKLIEFMDTFFFILRKNNHQITVLHVYHHVSMLNIWWFVMNWVPCGHSYFGATLNSFIHV
Mole.XP_0373757 SKLIEFMDTFFFILRKNNHQITVLHVYHHASMLNIWWFVMNWVPCGHSYFGATLNSFIHV
Bos.NP_00104006 SKLIEFMDTFFFILRKNNHQITVLHVYHHASMLNIWWFVMNWVPCGHSYFGATLNSFIHV
BlueWhale.XP_03 SKLIEFMDTFFFILRKNNHQVTVLHVYHHASMLNIWWFVMNWVPCGHSYFGATLNSFIHV
Dolphin.XP_0198 SKLIEFMDTFFFILRKNYHQVTVLHVYHHASMLNIWWFVMNWVPCGHSYFGATLNSFIHV
Gallus.NP_00118 SKLIEFMDTFFFILRKNNHQITVLHVYHHATMLNIWWFVMNWVPCGHSYFGATLNSFIHV
Duck.NP_0012973 SKLIEFMDTFFFILRKNNHQITVLHVYHHATMLNIWWFVMNWVPCGHSYFGATLNSFIHV
Falcon.XP_03724 SKLIEFMDTFFFILRKNNHQITVLHVYHHATMLNIWWFVMNWVPCGHSYFGATLNSFIHV
Turtle.XP_03775 SKLIEFMDTFFFILRKNHYQITVLHVYHHASMLNIWWFVMNWVPCGHSYFGATLNSFIHV
Komodo.KAF72468 SKLIEFMDTFFFILRKNNHQITVLHVYHHATMLNIWWFVMNWVPCGHSYFGATLNSFIHV
Xenopus.NP_0010 SKLIEFMDTFFFILRKNNHQITVLHVYHHASMLNIWWFVMNWVPCGHSYFGATLNSFIHV
Salmo.NP_001130 SKVIEFMDTFFFILRKNNHQITFLHIYHHASMLNIWWFVMNWVPCGHSYFGASLNSFVHV
Pike.AFR36912.1 SKLIEFMDTFFFILRKNNHQITFLHIYHHASMLNIWWFVMNWVPCGHCYFGACLNSFIHV
Croaker.NP_0012 SKLIEFMDTFFFILRKNNHQITFLHIYHHASMLNIWWFVMNWVPCGHSYFGASLNSFVHV
Rockfish.XP_037 SKLIEFMDTFFFIVRKNNHQITFLHIYHHASMLNIWWFVMNWVPCGHSYFGASLNSFVHV
AmSole.BBL33563 SKLIEFMDTFFFILRKNNHQITFLHIYHHASMLNIWWFVMNWIPCGHSFFGASLNSFVHV
Eel.XP_03525699 SKLIEFMDTFFFILRKNNHQITFLHVYHHASMPSIWWFVMNWVPCGHSYFGASLNSFIHV
Sturgeon.XP_033 SKLIEFMDTFFFILRKNNHQITFLHVYHHATMPNIWWFVMNWVPCGHSYFGASFNSFIHV
Zebrafish.NP_95 SKLIEFMDTFFFILRKNNHQITFLHVYHHATMLNIWWFVMNWVPCGHSYFGATFNSFIHV
Eshark.XP_00789 SKVIEFMDTFFFILRKNYHQITILHVYHHATMFNIWWFVMNWVPCGHSFFGATLNSFIHV
WhaleShark.XP_0 SKVIEFMDTFFFILRKNYHQITVLHVYHHATMFNIWWFVMNWVPCGHSFFGATLNSFIHV
                **:**********:*** :*:*.**:***.:* .********:****.:*** :***:**

HumanElovl5     LMYSYYGLSSVPSMRPYLWWKKYITQGQLLQFVLTIIQTSCGVIWPCTFPLGWLYFQIGY
Macaca.NP_00130 LMYSYYGLSSVPSMRPYLWWKKYITQGQLLQFVLTIIQTSCGVIWPCTFPLGWLYFQIGY
SeaLion.XP_0274 LMYSYYGLSSVPSVRPYLWWKKYITQGQLLQFVLTIIQTSCGVLWPCTFPLGWLYFQIGY
Bat.XP_03626954 LMYSYYGLSSVPSMRPYLWWKKYITQGQLIQFVLTIIQTTCGVIWPCSFPLGWLYFQIGY
FruitBat.XP_037 LMYSYYGLSSVPSMRPYLWWKKYITQGQLLQFVLTIVQTSCGVVWPCSFPLGWLYFQIGY
Mouse.NP_599016 LMYSYYGLSSIPSMRPYLWWKKYITQGQLVQFVLTIIQTTCGVFWPCSFPLGWLFFQIGY
Rat.NP_599209.1 LMYSYYGLSSVPSMRPYLWWKKYITQGQLVQFVLTIIQTSCGVIWPCSFPLGWLYFQIGY
Beaver.JAV42152 LMYSYYGLSSVPSMRPYLWWKKYITQGQLIQFVLTIIQTSCGVLWPCSFPLGWLYFQIGY
Pangolin.XP_017 LMYSYYGLSSVPSMRPYLWWKKYITQGQLLQFVLTIIQTSCGVVWPCTFPLGWLYFQIGY
ChPangolin.XP_0 LMYSYYGLSSVPSMRPYLWWKKYITQGQLLQFVLTIIQTSCGVVWPCAFPLGWLYFQIGY
Mole.XP_0373757 LMYSYYALSSVPSMRPYLWWKKYITQGQLLQFVLTIIQTTCGVIWRCPFPLGWLYFQIGY
Bos.NP_00104006 LMYSYYGLSSIPSMRPYLWWKKYITQGQLLQFVLTIIQTSCGVIWPCTFPLGWLYFQIGY
BlueWhale.XP_03 LMYSYYGLSSVPSMRPYLWWKKYITQGQLLQFVLTIIQTSCGVIWPCAFPLGWLYFQIGY
Dolphin.XP_0198 LMYSYYGLSSVPSMRPYLWWKKYITQGQLVQFVLTILQTSCGVIWPCAFPLGWLYFQIGY
Gallus.NP_00118 LMYSYYGLSAVPAMRPYLWWKKYITQGQLIQFVLTIFQTSCGVVWPCAFPQGWLYFQIFY
Duck.NP_0012973 LMYSYYGLSAVPAMRPYLWWKKYITQGQLTQFVLTIFQTSCGVVWPCAFPQGWLYFQISY
Falcon.XP_03724 LMYSYYGLSAVPAMRPYLWWKKYITQGQLIQFVLTIFQTSCGVVWPCAFPQGWLYFQISY
Turtle.XP_03775 LMYSYYGLSAIPAMRPYLWWKKYITQGQLIQFVLTIFQTSCGVVWPCAFPMGWLYFQIFY
Komodo.KAF72468 LMYSYYGLSAIPSMRPYLWWKKYITQGQLIQFVLTIFQTSCGVVWPCPFPLGWLYFQIGY
Xenopus.NP_0010 LMYSYYGLSAIPAMRPYLWWKKYITQCQLTQFVLTMTQTTCAMIWPCKFPMGWLYFQNCY
Salmo.NP_001130 LMYSYYGLSAVPAIRPYLWWKKYITQGQLIQFFLTMSQTICAVIWPCGFPRGWLFFQIFY
Pike.AFR36912.1 LMYSYYGLSAIPTMRPYLWWKKYITQGQLIQFFLTMTQTICAVIWPCGFPKGWLYFQISY
Croaker.NP_0012 VMYSYYGLSAIPAMRPYLWWKKYITQLQLTQFFLTMSQTLCAVVWPCGFPMGWLYFQISY
Rockfish.XP_037 VMYSYYGLSAIPAIRPYLWWKKYITQFQLIQFFLTMFQTMCAVVWPCGFPKGWLSFQISY
AmSole.BBL33563 VMYSYYGLSAIPAMRPYLWWKRYITQLQLIQFFLTVSHTMCAVIWPCGFPMGWLFFQISY
Eel.XP_03525699 LMYSYYGLSAVPALRPYLWWKKYITQGQLIQFVMTMIQTSCAVVWPCGFPRGWLYFQISY
Sturgeon.XP_033 LMYSYYGLSAIPAVRPYLWWKKYITQGQLIQFVLTMFQTTCAVFRPCGFPMGWLYFQICY
Zebrafish.NP_95 LMYSYYGLSAVPALRPYLWWKKYITQGQLVQFVLTMFQTSCAVVWPCGFPMGWLYFQISY
Eshark.XP_00789 LMYSYYGLSAIPAMRPYLWWKKYITQGQLLQFVMTIIQTSFGVVWPCGFPSGWLYFQIGY
```

Spinocerebellar ataxia 38: structure-function analysis shows ELOVL5 G230V is proteotoxic, conformationally altered and a mutational hotspot

Ferrero et al.

```
WhaleShark.XP_0 LMYSYYGLSAIPAMRPYLWWKKYITQGQLLQFVLTIIQTSFGVIWPCGFPSGWLYFQIGY
                :*****.**::*::*******:**** ** **.:*: :*  .:.  * ** *** **  *

HumanElovl5     MISLIALFTNFYIQTYNKKGASRRKDHLKDHQNGSMAAVNGHTNSFSPLENNVKPRKLRK
Macaca.NP_00130 MISLIALFTNFYIQTYNKKGASRRKDHLKDHQNGSVAAVNGHTNSFSPLENNVKPRKLRK
SeaLion.XP_0274 MISLITLFTNFYVQTYNKKGASRRKEHLKDHQNGSVAAVNGHTNSF-SLENNVKPRKQRK
Bat.XP_03626954 MISLITLFTNFYIQTYNKQGASRRKEHLKDHQNGSLTAANGHTNSFSSLENNVKLRKQRK
FruitBat.XP_037 MISLIILFTNFYIQTYNKKGASRRKERLRDHQNGSVTAANGHTNSFSSLGNSTKPRKQRK
Mouse.NP_599016 MISLIALFTNFYIQTYNKKGASRRKDHLKGHQNGSVAAVNGHTNSFPSLENSVKPRKQRK
Rat.NP_599209.1 MISLIALFTNFYIQTYNKKGASRRKEHLKGHQNGSMTAVNGHTNNFASLENSVTSRKQRK
Beaver.JAV42152 MISLIALFTNFYIQTYNKKGASRRRDHLKDHLNGSATVVNGHTNTFSSLENNVKPRKQRK
Pangolin.XP_017 MISLIVLFTNFYVQTYNKKGASRRREYLKDHQNGSMAAMNGHAGSFSSLGSSAKPRKLRK
ChPangolin.XP_0 MISLIVLFTNFYVQTYNKKGASRRREYLKDHQNGSMAAVNGHAGSFSSLGNSVKPRKLRK
Mole.XP_0373757 MISLITLFTNFYIQTYNKKGASRRKEHLKEHQNGSVAAVNGHTNSFSTLENNVKLRKQRK
Bos.NP_00104006 MISLITLFTNFYIQTYNKKGVSRRREHQKDHQNGSVAAVNGHISSFSSLENNVKPRKQRK
BlueWhale.XP_03 MISLIALFTNFYIQTYNKKGASRRREHQKDHQNGSVTAVNGHTSSFSSLENNVKPRKQRK
Dolphin.XP_0198 MISLIALFTNFYIQTYNKKGASRRRE----HQNGSVTAVNGHTSSFSSLEHSVKPRKQRK
Gallus.NP_00118 MISLIILFTNFYIQTYNKKASSRRKE----YQNGSTATVNGYTNSFSSLENNVKQRKQRK
Duck.NP_0012973 MISLIILFTNFYIQTYNKKASSRRKE----YQNGSAATVNGYTNSFSSLENNVKQRKQRK
Falcon.XP_03724 MISLIILFTNFYIQTYNKKASSRRKE----YQNGSTATVNGYTNSSSSLENNVKQRKQRK
Turtle.XP_03775 MISLIILFTNFYIQTYKKKASSRRKE----YQNGSTAAVNGYTNSFSSLENNVKQRKQRK
Komodo.KAF72468 MITLIILFTNFYIQTYNKKAACRRKE----YQNGSTAAVNGYTNSFSSLENNVKQRKQRK
Xenopus.NP_0010 MISLIILFGNFYIKTYNKKTSSRRKE----YQNGSASAVNGHTNSFSSLEDNVKQRKQRQ
Salmo.NP_001130 MASLIAFFSNFYIQTYKKHRVSQKEY----HQNGSVDSLNGHANGVTPTE-TITHRKVRV
Pike.AFR36912.1 MVTLIALFSNFYIQTYKKRSVSQKKEC---HRNGSASPLNGHVSGVTPIE-TITHRKVRA
Croaker.NP_0012 MVTLIFLFSNFYVQTYKKHSVSLKKE----HQNGSPVSTNGHANGTPSLE-HAAHKKLRV
Rockfish.XP_037 MVTLIFLFSNFYIQAYKKHSGSLKKE----HQNGSPISTNGHANGTPSME-RTAYQKLRM
AmSole.BBL33563 MVTLIIILFSNFYIQTYQKHSRSLQKD----HRNGSPASRNGHANGKPSAE-QPAHKKLRV
Eel.XP_03525699 MVTLIALFSNFYIQTYQKQGAFRRKE----HQNGSAAAMNGHSNGVSPAE-DLTRRKLRV
Sturgeon.XP_033 LIVLIVLFSNFYIQTYKKRGVSRRKE----CQNGSAASLNGHANGVSSVD-NVTQRKLRK
Zebrafish.NP_95 MVTLILLFSNFYIQTYKKRSGSRKSD----YPNGS---VNGHTNGVMSSE-KIKHRKARA
Eshark.XP_00789 MISLIVLFTNFYIQTYQKKSASRTKD----CLNGS---ANGHTNGFSAQTDHYSAKKTH-
WhaleShark.XP_0 MISLIILFTNFYIQTYKKKSAFKSKD----CLNGSTIAANGHTNGLSTQMDHYSEKKTH-
                :  ** :* ***:::*:*:             ***    **: .   .       :* :

HumanElovl5     D
Macaca.NP_00130 D
SeaLion.XP_0274 D
Bat.XP_03626954 D
FruitBat.XP_037 D
Mouse.NP_599016 D
Rat.NP_599209.1 D
Beaver.JAV42152 D
Pangolin.XP_017 D
ChPangolin.XP_0 D
Mole.XP_0373757 D
Bos.NP_00104006 D
BlueWhale.XP_03 D
Dolphin.XP_0198 D
Gallus.NP_00118 D
Duck.NP_0012973 D
Falcon.XP_03724 D
Turtle.XP_03775 D
Komodo.KAF72468 D
Xenopus.NP_0010 D
Salmo.NP_001130 D
Pike.AFR36912.1 D
Croaker.NP_0012 D
Rockfish.XP_037 D
AmSole.BBL33563 D
Eel.XP_03525699 D
Sturgeon.XP_033 D
Zebrafish.NP_95 D
Eshark.XP_00789 -
WhaleShark.XP_0 -
```

Spinocerebellar ataxia 38: structure-function analysis shows ELOVL5 G230V is proteotoxic, conformationally altered and a mutational hotspot

Ferrero et al.

Supplementary Table 6 ELOVL2 Protein sequences for Multiple Sequence Alignment

	Accession no.	Species	Length (AA)
1	Q9NXB9.2	*Homo sapiens* (Human)	296
2	XP_028703673.1	*Macaca mulatta* (Rhesus macaque)	296
3	NP_062296.1	*Mus musculus* (Mouse)	292
4	XP_032740498.1	*Rattus norvegicus* (Rat)	296
5	XP_027380089.1	*Bos indicus* x *Bos taurus* (Bovine)	294
6	NP_001184237.1	*Gallus gallus* (Chicken)	297
7	NP_001016159.1	*Xenopus tropicalis* (Frog)	296
8	NP_001130025.1	*Salmo salar* (Salmon)	287
9	XP_027307376.1	*Anas platyrhynchos* (Duck)	296
10	XP_007900820.1	*Callorhinchus milii* (Elephant shark)	300
11	XP_036725964.1	*Balaenoptera musculus* (Blue whale)	295
12	AGR34076.1	*Oncorhynchus masou* (Cherry salmon)	287
13	XP_005162628.1	*Danio rerio* (Zebrafish)	295
14	ETE65396.1	*Ophiophagus hannah* (King cobra)	301
15	XP_036301996.1	*Pipistrellus kuhlii* (Bat)	296

Spinocerebellar ataxia 38: structure-function analysis shows ELOVL5 G230V is proteotoxic, conformationally altered and a mutational hotspot

Ferrero et al.

Supplementary Table 7 MSA of ELOVL5 (30 sequences) and ELOVL2 (15 Sequences)

```
CLUSTAL format alignment by MAFFT (v7.505) Blosum 62/L-INS-i/Gap open penalty 1.53
HumanElovl5     --------MEHFDASLSTYFKALLGPRDTRVKGWFLLDNYIPTFICSVIYLLIVWLGPKY
Macaca.NP_00130 --------MEHFDASLSTYFKALLGPRDTRVKGWFLLDNYIPTFICSVIYLLIVWLGPKY
SeaLion.XP_0274 --------MENFDASLSTYFKALLGPRDTRVKGWFLLDNYIPTFICSVIYLLIVWLGPKY
Bat.XP_03626954 --------MEHFDASLSTYFKALLGPRDTRVKGWFLLDNYIPTFICSTIYLLIVWLGPKY
FruitBat.XP_037 --------MENFDASLSTYFKAWLGPRDTRVKGWFLLDNYIPTFICSVIYLLIVWLGPKY
Mouse.NP_599016 --------MEHFDASLSTYFKAFLGPRDTRVKGWFLLDNYIPTFVCSVIYLLIVWLGPKY
Rat.NP_599209.1 --------MEHFDASLSTYFRALLGPRDTRVKGWFLLDNYIPTFVCSAIYLLIVWLGPKY
Beaver.JAV42152 --------MENFDASLSTYFRAWLGPRDTRVKGWFLLDNYIPTFVCSVIYLLIVWLGPKY
Pangolin.XP_017 --------MENFDASLSTYFKALLGPRDTRVKGWFLLDNYIPTFICSIIYLLIVWLGPKY
ChPangolin.XP_0 --------MENFDASLSTYFKALLGPRDTRVKGWFLMDNYIPTFICSIIYLLIVWLGPKY
Mole.XP_0373757 --------MENFDASLSTYFKALLGPRDTRVKGWFLLDNYIPTFICSVIYLLIVWLGPKY
Bos.NP_00104006 --------MEHFDASLSTYFRAWLGPRDTRVEGWFLLDNYVPTLVCSILYLLIVWLGPKY
BlueWhale.XP_03 --------MEHFDASLSTYFQALLGPRDLRVKGWFLLDNYIPTLLCSILYLLIVWLGPKY
Dolphin.XP_0198 --------MEHFDASLSTYFQALLGPRDPRVKGWFLLDSYIPTLLCSILYLLIVWLGPKY
Gallus.NP_00118 --------MERLDKTINSYLDVWLGPRDPRVKGWLLLENYTPTFIFSVLYLLIVWLGPKY
Duck.NP_0012973 --------MERLDKTINSYLDVWLGPRDPRVKGWLLLENYTPTFIFSVLYLLIVWLGPKY
Falcon.XP_03724 --------MEFLDKTINSYLDVWLGPRDPRVKGWLLLENYTPTFIFSVLYLLIVWLGPKY
Turtle.XP_03775 --------MESLDQTINSYLDVWFGPRDPRVKGWLLLDNYIPTFIFSVLYLLIVWLGPKY
Komodo.KAF72468 --------MESLDKTINSYLDVWLGPRDPRVKGWLLLTNYTPTFIFSVLYLLIVWLGPKY
Xenopus.NP_0010 --------MEVLDKAVNGYIDHLLGPKDPRVRGWLLLDNYVPTILFTALYLFIVWRGPKY
Salmo.NP_001130 --------MEAFNHKLNTYIDSWMGPRDERVQGWLLLDNYPPTFALTLMYLLIVWLGPKY
Pike.AFR36912.1 --------METFNQRLNTFIDSWMGPRDERVRGWLLLDNYPPTFALTVIYLLIVWAGPKF
Croaker.NP_0012 --------METFNHKLNTYLESWMGPRDQRVRGWLLLDNYPPTFALTVMYLVIVWMGPKY
Rockfish.XP_037 --------METFNHRLNTYLESWMGPRDQRVRGWLLLDNYPPTFALTIMYLLIVWMGPKY
AmSole.BBL33563 --------METFNHKLNAYIDSWLGPRDQRVRGWLLLDNYPPTFALTVMYLLIVWLGPKY
Eel.XP_03525699 -----MD-MEMFNHRLNTYIDSWMGPRDQRVRGWLLLDDYPPTFALTVAYLLIVWMGPKY
Sturgeon.XP_033 --------MEALNQATNAYIDSWMGPRDTRVRGWFLLDNFSPTVVLTLLYLFVVWIGPKY
Zebrafish.NP_95 --------METFSHRVNSYIDSWMGPRDLRVTGWFLLDDYIPTFIFTVMYLLIVWMGPKY
Eshark.XP_00789 --------MGFADQTLNSYFDSWFGPRDPRVEGFLLLDNYPPTFLLTLIYLFVVWAGPKY
WhaleShark.XP_0 --------MEAVDRTLNYYFVSWFGPRDSRVEGFFLLDKYPPTFILSLLYLFVVWAGPKY
Macaca.XP_02870 -----MEHLKAFDDEINAFLDNMFGPRDSRVRGWFMLDSYLPTFFLTVIYLLSIWLGNKY
Human.sp|Q9NXB9 -----MEHLKAFDDEINAFLDNMFGPRDSRVRGWFMLDSYLPTFFLTVMYLLSIWLGNKY
XP_036301996.1  -----MELLKAFDDKINAFLDNMFGPRDTRVRGWFMLDSYLPTFSLTVMYLLSIWLGNKY
Bos.XP_02738008 -----MEHLKAFDDEVNAFLDNMFGPRDSRVRGWFMLDSYLPTFSLTVLYLLSIWLGNRC
Bwhale.XP_03672 -----MEHLKAFDDEINAFLDNMFGPRDSRVRGWFMLDSYLPTFSLTVLYLLCIWLGNKY
Rat.XP_03274049 -----MEQLKAFDNEVNAFLDNMFGPRDSRVRGWFLLDSYLPTFTLTIMYLLSIWLGNKY
Mouse.NP_062296 -----MEQLKAFDNEVNAFLDNMFGPRDSRVRGWFLLDSYLPTFILTITYLLSIWLGNKY
Gallus.NP_00118 -----MEHLKAFDQEVNAFVDYMFGPRDARVRGWLLLDSYLPTFFLTVAYLLCIWLGNKF
Duck.XP_0273073 -----MEHLKAFDQEINEFVDYMFGPRDSRVRGWLLLDSYLPTFFLTCAYLLCIWLGNKF
Frog.NP_0010161 -----MEHIEKIDQEVNAFVDYLFGPRDTRTRGWLMLDSYLPTLFLTLLYFLSIWLGTKY
Shark.XP_007900 MMPTVANQIETLDKRVNSFVDYMFGDRDPRVRGWLMLDSYLPTFFLTLLYLLAIWLGTKF
ETE65396.1      ----------------------MFGERDPRVRGWLMLDSYLPTFALTMLYLLFIWLGTMF
Salmo.NP_001130 -----MNHLQSLDERLNKLFYFLFEDRDSRVRGWLLLDSYLPTLSLTILYLLSVYLGSKY
Cherry          -----MNHLQSLDERLNALFYFLFEDRDSRVRGWLLLDSYLPTLSLTILYLLTVYLGSKY
Zebrafish.XP_00 -----MESYEKIDKLLNSVVDSLFGERDTRVRGWLLLDSYTPTFLLTITYLLTIYLGTKY
                                       :  :* *. *:::: .: **.  :  *:. :: *

HumanElovl5     MRNKQPFSCRGILVVYNLGLTLLSLYMFCE----------------------LVTGVWEG
Macaca.NP_00130 MRNKQPFSCRGILVVYNLGLTLLSLYMFCE----------------------LVTGVWEG
SeaLion.XP_0274 MKNRQPFSCRGILVLYNLGLTLLSLYMFCE----------------------LVKGVWEG
Bat.XP_03626954 MKNRQPLACRKILVVYNLGLTLLSLYMFCE----------------------LVTGVWEG
FruitBat.XP_037 MKNRQPFSCRGILVVYNLGLTLLSLYMFCE----------------------LVTGVWEG
Mouse.NP_599016 MKNRQPFSCRGILQLYNLGLTLLSLYMFYE----------------------LVTGVWEG
Rat.NP_599209.1 MKNRQPFSCRGILVVYNLGLTLLSLYMFYE----------------------LVTGVWEG
Beaver.JAV42152 MKNRQPFSCRGILVVYNLGLTLLSLYMFYE----------------------LVTGVWEG
Pangolin.XP_017 MKNRQPFSCRGILVVYNLGLTLLSLYMFYE----------------------LVTGVWEG
ChPangolin.XP_0 MKNRQPFSCRGILVVYNLGLTLLSLYMFYE----------------------LVTGVWEG
Mole.XP_0373757 MKNRQAFSCRGILVVYNLGLTLLSLYMFCE----------------------LVTGVWEG
Bos.NP_00104006 MKTRQPFSCRGILVVYNLGLTLLSLYMFCE----------------------LVTGVWEG
BlueWhale.XP_03 MKNRPPFSCRGILVVYNLGLTLLSLYMFCE----------------------LVTGVWEG
Dolphin.XP_0198 MKNRPPFSCRGILVLYNLGLTLLSLYMFCE----------------------LVTGVWEG
Gallus.NP_00118 MRNKQPFSCRGILVVYNLGLTLLSLYMFYE----------------------LVTGVWEG
```

Spinocerebellar ataxia 38: structure-function analysis shows ELOVL5 G230V is proteotoxic, conformationally altered and a mutational hotspot

Ferrero et al.

```
Duck.NP_0012973 MRNKQPFSCRGILVVYNLGLTLLSLYMFYE----------------------LVTGVWEG
Falcon.XP_03724 MRNKQPFSCRGILVVYNLGLTLLSLYMFYE----------------------LVTGVWEG
Turtle.XP_03775 MQNKQPFSCRGILVVYNVGLTLLSLYMFYE----------------------LVTGVWEG
Komodo.KAF72468 MQKRPPFSCRGILVVYNLGLTLLSLYMFYE----------------------LVTGVWEG
Xenopus.NP_0010 MQNRPPVSCRGILVVYNLGLTLLSLYMFYE----------------------LVTGVWEG
Salmo.NP_001130 MRHRQPVSCQGLLVLYNLALTLLSFYMFYE----------------------MVSAVWQG
Pike.AFR36912.1 MRHRQPLSCRGFLVVYNLGLALLSLYMFSE----------------------MVSAVWVG
Croaker.NP_0012 MKHRQPYSCRGLLVLYNLGLTLLSFYMFYE----------------------LVTAVWHG
Rockfish.XP_037 MKHRQPYSCRGLLVLYNLGLTLLSFYMFYE----------------------LVSAVRHG
AmSole.BBL33563 MKHRQPYSCRGLLVVYNLGLTLLSFYMFYE----------------------LVGAVWHG
Eel.XP_03525699 MKNRQPFSCRGLLVVYNLGLTLLSLYMFCE----------------------LVNGMWQG
Sturgeon.XP_033 MKNKEPFSCRGVLVVYNIGLTLLSFYMFYE----------------------LVTGAWQG
Zebrafish.NP_95 MKNRQAYSCRALLVPYNLCLTLLSLYMFYE----------------------LVMSVYQG
Eshark.XP_00789 MKNKAPVSCRGTLVVYNIGLTLLSLYMFYE----------------------LLTGVWEG
WhaleShark.XP_0 MQNKAPLSSRGILVVYNIGLTLLSLYMFCE----------------------LVRGVWDG
Macaca.XP_02870 MKNRPALSLRGILTLYNLGITLLSAYMLAE----------------------LILSTWEG
Human.sp|Q9NXB9 MKNRPALSLRGILTLYNLGITLLSAYMLAE----------------------LILSTWEG
XP_036301996.1  MKNRPALSLRSILTLYNLGITLLSMYMLAE----------------------LILSTWEG
Bos.XP_02738008 MRNRPALSLRGILTLYNLGITLLSAYMLAE----------------------LILSSWEG
Bwhale.XP_03672 MRSRAALSLRGILTLYNLGITLLSAYMLAE----------------------LILSSWEG
Rat.XP_03274049 MKNRPALSLRGILTLYNLGITLLSAYMLVE----------------------LVLSSWEG
Mouse.NP_062296 MKNRPALSLRGILTLYNLAITLLSAYMLVE----------------------LILSSWEG
Gallus.NP_00118 MKNRQPFSLKAHLIVYNLGITLLSLYMLIE----------------------LILATWEG
Duck.XP_0273073 MKNRPPFSLRAHLIVYNLGITLLSMYMLIE----------------------LILATWEG
Frog.NP_0010161 MQNRPAFSLRGHLIVYNLVVTLLSLYMLIE----------------------LILSTWEG
Shark.XP_007900 MKDRPAFFLRTHLIIYNLGVMLLSLYMFIE----------------------LILSSWEG
ETE65396.1      MKNRPALSLRGLLIVYNFGVTVLSFYMLIEVRLLNRRILNFLFLAFYSADWDLILATWEG
Salmo.NP_001130 MRNRPAYSLKGVLQVYNFSVTMLSLYMLVE----------------------LVSATLSA
Cherry          MRNRPAYSLKGVLQVYNFSVTMLSLYMLVE----------------------LVLATLSA
Zebrafish.XP_00 MRNRPAYSLKNVLLLYNFSVTVLSFYMLVE----------------------LISAVWSA
                *: : .   :  *  **. : :** **: *                      :: .   .

HumanElovl5     KYNFFCQGTRTAGESDMKIIRVLWWYYFSKLIEFMDTFFFILRKNNHQITVLHVYHHASM
Macaca.NP_00130 KYNFFCQGTRTAGESDMKIIRVLRWYYFSKLIEFMDTFFFILRKNNHQITVLHVYHHASM
SeaLion.XP_0274 RYNFFCQGTRSAGEADMKIIRVLWWYYFSKLIEFMDTFFFILRKNNHQITVLHVYHHASM
Bat.XP_03626954 QYNFFCQGTRSAGEADMKIIRVLWWYYFSKLIEFMDTFFFILRKNNHQITVLHVYHHASM
FruitBat.XP_037 KYNFFCQGTRSAGEADMKIIRVLWWYYFSKLIEFMDTFFFILRKNNHQVTVLHVYHHASM
Mouse.NP_599016 KYNFFCQGTRSAGESDMKIIRVLWWYYFSKLIEFMDTFFFILRKNNHQITVLHVYHHATM
Rat.NP_599209.1 KYNFFCQGTRSAGESDMKVIRVLWWYYFSKLIEFMDTFFFILRKNNHQITVLHVYHHATM
Beaver.JAV42152 KYNFFCQGTRSAGESDMKIIQVLWWYYFSKLIEFMDTFFFILRKNNHQITVLHVYHHASM
Pangolin.XP_017 RYNFFCQGTRSSGEADVKVIRVLWWYYFSKLIEFMDTFFFILRKNNHQITVLHVYHHVSM
ChPangolin.XP_0 RYNFFCQGTRSSGEADVKVIRVLWWYYFSKLIEFMDTFFFILRKNNHQITVLHVYHHVSM
Mole.XP_0373757 KYNFFCQDTRSAGEADMKIIHVLWWYYFSKLIEFMDTFFFILRKNNHQITVLHVYHHASM
Bos.NP_00104006 QYNFFCQGTRSGGEADMKIIRVLWWYYFSKLIEFMDTFFFILRKNNHQITVLHVYHHASM
BlueWhale.XP_03 QYNFFCQGTRSRGEADMKIVRVLWWYYFSKLIEFMDTFFFILRKNNHQVTVLHVYHHASM
Dolphin.XP_0198 QYNFFCQGTRSGGEADMKIIRVLWWYYFSKLIEFMDTFFFILRKNYHQVTVLHVYHHASM
Gallus.NP_00118 GYNFYCQDTHSGGEADMKIIRVLWWYYFSKLIEFMDTFFFILRKNNHQITVLHVYHHATM
Duck.NP_0012973 GYNFFCQDTHSGGEADMKIIRVLWWYYFSKLIEFMDTFFFILRKNNHQITVLHVYHHATM
Falcon.XP_03724 GYNFFCQDTHSGGEADMKIIRVLWWYYFSKLIEFMDTFFFILRKNNHQITVLHVYHHATM
Turtle.XP_03775 GYNFFCQDTHSGGEADMKIVRVLWWYYFSKLIEFMDTFFFILRKNHYQITVLHVYHHASM
Komodo.KAF72468 GYNFFCQDTHSGGEADMKIIRVLWWYYFSKLIEFMDTFFFILRKNNHQITVLHVYHHATM
Xenopus.NP_0010 GYNFFCQDTNSGGDADTKIVRVLWWYYFSKLIEFMDTFFFILRKNNHQITVLHVYHHASM
Salmo.NP_001130 GYNFYCQDTHSAGETDTKIINVLWWYYFSKVIEFMDTFFFILRKNNHQITFLHIYHHASM
Pike.AFR36912.1 GYHFYCQDTRSAGEADTRIIHVLWWYYFSKLIEFMDTFFFILRKNNHQITFLHIYHHASM
Croaker.NP_0012 GYNFYCQDIHSAQEVDNKIINVLWWYYFSKLIEFMDTFFFILRKNNHQITFLHIYHHASM
Rockfish.XP_037 GYNFYCQDTHSAQEVDNKIMNVLWWYYFSKLIEFMDTFFFIVRKNNHQITFLHIYHHASM
AmSole.BBL33563 DYNFYCQNTHSAPEVDKKVINVLWWYYFSKLIEFMDTFFFILRKNNHQITFLHIYHHASM
Eel.XP_03525699 NYNFFCQDTHSAGEADTKIINVLWWYYFSKLIEFMDTFFFILRKNNHQITFLHVYHHASM
Sturgeon.XP_033 GYNFFCQDTHSGGEADMKIVRVLWWYYFSKLIEFMDTFFFILRKNNHQITFLHVYHHATM
Zebrafish.NP_95 GYNFFCQNTHSGGDADNRMMNVLWWYYFSKLIEFMDTFFFILRKNNHQITFLHVYHHATM
Eshark.XP_00789 GYSIFCQDTHSAGKADLKIVRVLWWYYFSKVIEFMDTFFFILRKNYHQITILHVYHHATM
WhaleShark.XP_0 GYSIFCQNTHNAGEADLKIIRVLWWYYFSKVIEFMDTFFFILRKNYHQITVLHVYHHATM
Macaca.XP_02870 GYNLQCQDLTSAGEADIRVAKVLWWYYFSKSVEFLDTIFFVLRKKTSQITFLHVYHHASM
Human.sp|Q9NXB9 GYNLQCQDLTSAGEADIRVAKVLWWYYFSKSVEFLDTIFFVLRKKTSQITFLHVYHHASM
XP_036301996.1  GYNLQCQDLASAGEADVRVAKVLWWYYFSKLIEFLDTIFFVLRKKTSQITFLHVYHHASM
```

Spinocerebellar ataxia 38: structure-function analysis shows ELOVL5 G230V is proteotoxic, conformationally altered and a mutational hotspot

Ferrero et al.

```
Bos.XP_02738008 GYNLQCQDLTSAGEADIRVARVLWWYYFSKLIEFLDTIFFVLRKKTSQVTFLHVYHHASM
Bwhale.XP_03672 GYNLQCQDLTSAGEADIRVAGVLWWYYFSKLIEFLDTIFFVLRKKTSQITFLHVYHHASM
Rat.XP_03274049 GYNLQCQNLDSAGEGDIRVAKVLWWYYFSKLVEFLDTIFFVLRKKTSQITFLHVYHHASM
Mouse.NP_062296 GYNLQCQNLDSAGEGDVRVAKVLWWYYFSKLVEFLDTIFFVLRKKTNQITFLHVYHHASM
Gallus.NP_00118 GYNLQCQNLHSAGEADIRVAKVLWWYYFSKVIEFADTIFFVLRKKSSQITFLHVYHHATM
Duck.XP_0273073 GYNLQCQNLHSAGAADIRVAKVLWWYYFSKVIEFMDTIFFVLRKKSSQITFLHVYHHATM
Frog.NP_0010161 GYNLQCQNLDSAGKADVRVAKVLWWYYFSKAIEFMDTIFFVLRKKNSQITFLHVYHHASM
Shark.XP_007900 AYYLQCQNLHSAGEVDIRVAKVLWWYYFSKAVEFMDTIFFVLRKKNSQITFLHVYHHATM
ETE65396.1      NYNLQCQNLISAGETDIRVARVLWWYYFSKVIEFADTVFFVLRKKNSQITFLHVYHHATM
Salmo.NP_001130 GYRLQCQGLHEAGEADLRVAKVLWWYYFSKVIEFLDTIFFVLRKKNSQITFLHVYHHASM
Cherry          GYRLQCQGLHEAGEADLRVAKVLWWYYFSKVIEFLDTIFFVLRKKNSQITFLHVYHHASM
Zebrafish.XP_00 GYRLQCQALDEVGEADIRVAKVLWWYYFSKLIEFLDTIFIVLRKKNSQISFLHVYHHASM
                 * : **        * ::  ** ****** :** **.*:::**:  *::.**:***.:*

HumanElovl5     LNIWWFVMNWVPCGHSYFGATLNSFIHVLMYSYYGLSSVPSMRPYLWWKKYITQGQLLQF
Macaca.NP_00130 LNIWWFVMNWVPCGHSYFGATLNSFIHVLMYSYYGLSSVPSMRPYLWWKKYITQGQLLQF
SeaLion.XP_0274 LNIWWFVMNWVPCGHSYFGATLNSFIHVLMYSYYGLSSVPSVRPYLWWKKYITQGQLLQF
Bat.XP_03626954 LNIWWFVMNWVPCGHSYFGATLNSFIHVLMYSYYGLSSVPSMRPYLWWKKYITQGQLIQF
FruitBat.XP_037 LNIWWFVMNWVPCGHSYFGATLNSFIHVLMYSYYGLSSVPSMRPYLWWKKYITQGQLLQF
Mouse.NP_599016 LNIWWFVMNWVPCGHSYFGATLNSFIHVLMYSYYGLSSIPSMRPYLWWKKYITQGQLVQF
Rat.NP_599209.1 LNIWWFVMNWVPCGHSYFGATLNSFIHVLMYSYYGLSSVPSMRPYLWWKKYITQGQLVQF
Beaver.JAV42152 LNIWWFVMNWVPCGHSYFGATLNSFIHVLMYSYYGLSSVPSMRPYLWWKKYITQGQLIQF
Pangolin.XP_017 LNIWWFVMNWVPCGHSYFGATLNSFIHVLMYSYYGLSSVPSMRPYLWWKKYITQGQLLQF
ChPangolin.XP_0 LNIWWFVMNWVPCGHSYFGATLNSFIHVLMYSYYGLSSVPSMRPYLWWKKYITQGQLLQF
Mole.XP_0373757 LNIWWFVMNWVPCGHSYFGATLNSFIHVLMYSYYALSSVPSMRPYLWWKKYITQGQLLQF
Bos.NP_00104006 LNIWWFVMNWVPCGHSYFGATLNSFIHVLMYSYYGLSSIPSMRPYLWWKKYITQGQLLQF
BlueWhale.XP_03 LNIWWFVMNWVPCGHSYFGATLNSFIHVLMYSYYGLSSVPSMRPYLWWKKYITQGQLLQF
Dolphin.XP_0198 LNIWWFVMNWVPCGHSYFGATLNSFIHVLMYSYYGLSSVPSMRPYLWWKKYITQGQLVQF
Gallus.NP_00118 LNIWWFVMNWVPCGHSYFGATLNSFIHVLMYSYYGLSAVPAMRPYLWWKKYITQGQLIQF
Duck.NP_0012973 LNIWWFVMNWVPCGHSYFGATLNSFIHVLMYSYYGLSAVPAMRPYLWWKKYITQGQLTQF
Falcon.XP_03724 LNIWWFVMNWVPCGHSYFGATLNSFIHVLMYSYYGLSAVPAMRPYLWWKKYITQGQLIQF
Turtle.XP_03775 LNIWWFVMNWVPCGHSYFGATLNSFIHVLMYSYYGLSAIPAMRPYLWWKKYITQGQLIQF
Komodo.KAF72468 LNIWWFVMNWVPCGHSYFGATLNSFIHVLMYSYYGLSAIPSMRPYLWWKKYITQGQLIQF
Xenopus.NP_0010 LNIWWFVMNWVPCGHSYFGATLNSFIHVLMYSYYGLSAIPAMRPYLWWKKYITQCQLTQF
Salmo.NP_001130 LNIWWFVMNWVPCGHSYFGASLNSFVHVLMYSYYGLSAVPAIRPYLWWKKYITQGQLIQF
Pike.AFR36912.1 LNIWWFVMNWVPCGHCYFGACLNSFIHVLMYSYYGLSAIPTMRPYLWWKKYITQGQLIQF
Croaker.NP_0012 LNIWWFVMNWVPCGHSYFGASLNSFVHVVMYSYYGLSAIPAMRPYLWWKKYITQLQLTQF
Rockfish.XP_037 LNIWWFVMNWVPCGHSYFGASLNSFVHVVMYSYYGLSAIPAIRPYLWWKKYITQFQLIQF
AmSole.BBL33563 LNIWWFVMNWIPCGHSFFGASLNSFVHVVMYSYYGLSAIPAMRPYLWWKRYITQLQLIQF
Eel.XP_03525699 PSIWWFVMNWVPCGHSYFGASLNSFIHVLMYSYYGLSAVPALRPYLWWKKYITQGQLIQF
Sturgeon.XP_033 PNIWWFVMNWVPCGHSYFGASFNSFIHVLMYSYYGLSAIPAVRPYLWWKKYITQGQLIQF
Zebrafish.NP_95 LNIWWFVMNWVPCGHSYFGATFNSFIHVLMYSYYGLSAVPALRPYLWWKKYITQGQLVQF
Eshark.XP_00789 FNIWWFVMNWVPCGHSFFGATLNSFIHVLMYSYYGLSAIPAMRPYLWWKKYITQGQLLQF
WhaleShark.XP_0 FNIWWFVMNWVPCGHSFFGATLNSFIHVLMYSYYGLSAIPAMRPYLWWKKYITQGQLLQF
Macaca.XP_02870 FNIWWCVLNWIPCGQSFFGPTLNSFIHILMYSYYGLSVFPSMHKYLWWKKYLTQAQLVQF
Human.sp|Q9NXB9 FNIWWCVLNWIPCGQSFFGPTLNSFIHILMYSYYGLSVFPSMHKYLWWKKYLTQAQLVQF
XP_036301996.1  FNIWWCVLNWIPCGQSFFGPTLNSFIHILMYSYYGLSVFPSMHKYLWWKKYLTQAQLVQF
Bos.XP_02738008 FNIWWCVLNWIPCGQSFFGPTLNSFIHILMYSYYGLSVFPSMHKYLWWKKYLTQAQLVQF
Bwhale.XP_03672 FNIWWCVLNWIPCGQSFFGPTLNSFIHILMYTYYGLSVFPSMHKYLWWKKYLTQAQLVQF
Rat.XP_03274049 FNIWWCVLNWIPCGQSFFGPTLNSFIHILMYSYYGLSVFPSMHRYLWWKKYLTQAQLVQF
Mouse.NP_062296 FNIWWCVLNWIPCGQSFFGPTLNSFIHILMYSYYGLSVFPSMHKYLWWKKYLTQAQLVQF
Gallus.NP_00118 FNIWWCVLNWIPCGQSFFGPTLNSFIHVLMYSYYGLSVIPSMRKYLWWKKYLTQAQLIQF
Duck.XP_0273073 FNIWWCVLNWIPCGQSFFGPTLNSFIHVLMYSYYGLSVIPSMRKYLWWKKYLTQAQLIQF
Frog.NP_0010161 FNIWWCVLNWIPCGQSFFGPTLNSFIHVLMYSYYGLSVIPSMHKYLWWKRYLTQAQLVQF
Shark.XP_007900 FNIWWCVLNWIPCGQSFFGPTLNSFIHVLMYSYYGLSTIPSMQPYLWWKKYLTQAQLVQF
ETE65396.1      FNIWWCVMNWIPCGQSFFGPTLNSFIHILMYSYYGLSVIPSMRKYLWWKKYLTQAQLIQF
Salmo.NP_001130 FNIWWCVLNWIPCGQSFFGPTLNSFIHVCMYSYYGLSTIPSMQKYLWWKRYLTQAQLIQF
Cherry          FNIWWCVLNWIPCGQSFFGPTLNSFIHVCMYSYYGLSTIPSMQKYLWWKRYLTQAQLIQF
Zebrafish.XP_00 FNIWWCVLNWIPCGQSFFGPTLNSFIHVLMYSYYGLATIPSMHKYLWWKRYLTQAQLVQF
                 .*** *:**:***:.:**. :***:*: **:**.*: .*::: *****:*:** ** **

HumanElovl5     VLTIIQTSCGVIWPCTFPLGWLYFQIGYMISLIALFTNFYIQTYNKKGASRRKDHLKDHQ
Macaca.NP_00130 VLTIIQTSCGVIWPCTFPLGWLYFQIGYMISLIALFTNFYIQTYNKKGASRRKDHLKDHQ
SeaLion.XP_0274 VLTIIQTSCGVLWPCTFPLGWLYFQIGYMISLITLFTNFYVQTYNKKGASRRKEHLKDHQ
Bat.XP_03626954 VLTIIQTTCGVIWPCSFPLGWLYFQIGYMISLITLFTNFYIQTYNKQGASRRKEHLKDHQ
```

Spinocerebellar ataxia 38: structure-function analysis shows ELOVL5 G230V is proteotoxic, conformationally altered and a mutational hotspot

Ferrero et al.

```
FruitBat.XP_037 VLTIVQTSCGVVWPCSFPLGWLYFQIGYMISLIILFTNFYIQTYNKKGASRRKERLRDHQ
Mouse.NP_599016 VLTIIQTTCGVFWPCSFPLGWLFFQIGYMISLIALFTNFYIQTYNKKGASRRKDHLKGHQ
Rat.NP_599209.1 VLTIIQTSCGVIWPCSFPLGWLYFQIGYMISLIALFTNFYIQTYNKKGASRRKEHLKGHQ
Beaver.JAV42152 VLTIIQTSCGVLWPCSFPLGWLYFQIGYMISLIALFTNFYIQTYNKKGASRRRDHLKDHL
Pangolin.XP_017 VLTIIQTSCGVVWPCTFPLGWLYFQIGYMISLIVLFTNFYVQTYNKKGASRRREYLKDHQ
ChPangolin.XP_0 VLTIIQTSCGVVWPCAFPLGWLYFQIGYMISLIVLFTNFYVQTYNKKGASRRREYLKDHQ
Mole.XP_0373757 VLTIIQTTCGVIWRCPFPLGWLYFQIGYMISLITLFTNFYIQTYNKKGASRRKEHLKEHQ
Bos.NP_00104006 VLTIIQTSCGVIWPCTFPLGWLYFQIGYMISLITLFTNFYIQTYNKKGVSRRREHQKDHQ
BlueWhale.XP_03 VLTIIQTSCGVIWPCAFPLGWLYFQIGYMISLIALFTNFYIQTYNKKGASRRREHQKDHQ
Dolphin.XP_0198 VLTILQTSCGVIWPCAFPLGWLYFQIGYMISLIALFTNFYIQTYNKKGASRRRE----HQ
Gallus.NP_00118 VLTIFQTSCGVVWPCAFPQGWLYFQIFYMISLIILFTNFYIQTYNKKASSRRKE----YQ
Duck.NP_0012973 VLTIFQTSCGVVWPCAFPQGWLYFQISYMISLIILFTNFYIQTYNKKASSRRKE----YQ
Falcon.XP_03724 VLTIFQTSCGVVWPCAFPQGWLYFQISYMISLIILFTNFYIQTYNKKASSRRKE----YQ
Turtle.XP_03775 VLTIFQTSCGVVWPCAFPMGWLYFQIFYMISLIILFTNFYIQTYKKKASSRRKE----YQ
Komodo.KAF72468 VLTIFQTSCGVVWPCPFPLGWLYFQIGYMITLIILFTNFYIQTYNKKAACRRKE----YQ
Xenopus.NP_0010 VLTMTQTTCAMIWPCKFPMGWLYFQNCYMISLIILFGNFYIKTYNKKTSSRRKE----YQ
Salmo.NP_001130 FLTMSQTICAVIWPCGFPRGWLFFQIFYMASLIAFFSNFYIQTYKKHRVSQKEY----HQ
Pike.AFR36912.1 FLTMTQTICAVIWPCGFPKGWLYFQISYMVTLIALFSNFYIQTYKKRSVSQKKEC---HR
Croaker.NP_0012 FLTMSQTLCAVVWPCGFPMGWLYFQISYMVTLIFLFSNFYVQTYKKHSVSLKKE----HQ
Rockfish.XP_037 FLTMFQTMCAVVWPCGFPKGWLSFQISYMVTLIFLFSNFYIQAYKKHSGSLKKE----HQ
AmSole.BBL33563 FLTVSHTMCAVIWPCGFPMGWLFFQISYMVTLIIILFSNFYIQTYQKHSRSLQKD----HR
Eel.XP_03525699 VMTMIQTSCAVVWPCGFPRGWLYFQISYMVTLIALFSNFYIQTYQKQGAFRRKE----HQ
Sturgeon.XP_033 VLTMFQTTCAVFRPCGFPMGWLYFQICYLIVLIVLFSNFYIQTYKKRGVSRRKE----CQ
Zebrafish.NP_95 VLTMFQTSCAVVWPCGFPMGWLYFQISYMVTLILLFSNFYIQTYKKRSGSRKSD----YP
Eshark.XP_00789 VMTIIQTSFGVVWPCGFPSGWLYFQIGYMISLIVLFTNFYIQTYQKKSASRTKD----CL
WhaleShark.XP_0 VLTIIQTSFGVIWPCGFPSGWLYFQIGYMISLIILFTNFYIQTYKKKSAFKSKD----CL
Macaca.XP_02870 VLTITHTMSAVVKPCGFPFGCLIFQSSYMLTLVILFLNFYVQTYRKKPMKKDMQ----EP
Human.sp|Q9NXB9 VLTITHTMSAVVKPCGFPFGCLIFQSSYMLTLVILFLNFYVQTYRKKPMKKDMQ----EP
XP_036301996.1  VLTITHTLSAVVKPCGFPFGCLIFQSSYMLTLVILFLNFYFQTYRKKPMKKETE----EL
Bos.XP_02738008 LLTITHTMSAVVRPCGFPLGCLIFQSSYMMTLVILFLNFYIQTYRKKPMKKAME----E-
Bwhale.XP_03672 VLTVTHTMSAVVRPCGFPFGCLIFQSSYMLTLVILFLNFYVQTYRKKPMKKDGE----EP
Rat.XP_03274049 VLTITHTLSAVVKPCGFPFGCLIFQSSYMMTLVILFLNFYIQTYRKKPGKKEMQ----EG
Mouse.NP_062296 VLTITHTLSAVVKPCGFPFGCLIFQSSYMMTLVILFLNFYIQTYRKKPVKKELQ----E-
Gallus.NP_00118 LLTIVHTLSAAVKPCGFPFGCLMFQSSYMATLVILFVNFYIKTYQKAPSRTAAK----ET
Duck.XP_0273073 LLTIVHTLSAAVKPCGFPFGCLMFQSSYMATLVILFVNFYIKTYRKAPSRTAVK----ET
Frog.NP_0010161 LLTITHTLSAAVKPCGFPFGCLMFQASYMATLVILFVNFYLKTYKKRPSKSDPN----GI
Shark.XP_007900 VLTITHTVSAVVKPCGFPIGCLMFQSSYMATLVILFVNFYVKTYRKTVVQDNKT----N-
ETE65396.1      LLTITHTLSAAVKPCGFPFGCLIFQVSYMATLVVLFINFYVKTYQKASSKANAK----EI
Salmo.NP_001130 ILTITHTLSAIVVPCGFPVGCLLFQFSYMATLVILFVNFYVQTYRKRRPEES--------
Cherry          ILTITHTLSAIVVPCGFPVGCLLFQFSYMATLVILFVNFYVQTYRKRRPEES--------
Zebrafish.XP_00 VLTITHTVSAWVVPCGFPLGCLKFQTFYMCTLVVLFVNFYIQTYKKRKTEGGRM------
                .:*: :*  . .  * ** * * **  *:  *: :* ***.::*.*

HumanElovl5     NGSMAAVNGHTNSFSPLENNVK-PRKLRKD
Macaca.NP_00130 NGSVAAVNGHTNSFSPLENNVK-PRKLRKD
SeaLion.XP_0274 NGSVAAVNGHTNSF-SLENNVK-PRKQRKD
Bat.XP_03626954 NGSLTAANGHTNSFSSLENNVK-LRKQRKD
FruitBat.XP_037 NGSVTAANGHTNSFSSLGNSTK-PRKQRKD
Mouse.NP_599016 NGSVAAVNGHTNSFPSLENSVK-PRKQRKD
Rat.NP_599209.1 NGSMTAVNGHTNNFASLENSVT-SRKQRKD
Beaver.JAV42152 NGSATVVNGHTNTFSSLENNVK-PRKQRKD
Pangolin.XP_017 NGSMAAMNGHAGSFSSLGSSAK-PRKLRKD
ChPangolin.XP_0 NGSMAAVNGHAGSFSSLGNSVK-PRKLRKD
Mole.XP_0373757 NGSVAAVNGHTNSFSTLENNVK-LRKQRKD
Bos.NP_00104006 NGSVAAVNGHISSFSSLENNVK-PRKQRKD
BlueWhale.XP_03 NGSVTAVNGHTSSFSSLENNVK-PRKQRKD
Dolphin.XP_0198 NGSVTAVNGHTSSFSSLEHSVK-PRKQRKD
Gallus.NP_00118 NGSTATVNGYTNSFSSLENNVK-QRKQRKD
Duck.NP_0012973 NGSAATVNGYTNSFSSLENNVK-QRKQRKD
Falcon.XP_03724 NGSTATVNGYTNSSSSLENNVK-QRKQRKD
Turtle.XP_03775 NGSTAAVNGYTNSFSSLENNVK-QRKQRKD
Komodo.KAF72468 NGSTAAVNGYTNSFSSLENNVK-QRKQRKD
Xenopus.NP_0010 NGSASAVNGHTNSFSSLEDNVK-QRKQRQD
Salmo.NP_001130 NGSVDSLNGHANGVTPTE-TIT-HRKVRVD
Pike.AFR36912.1 NGSASPLNGHVSGVTPIE-TIT-HRKVRAD
```

Spinocerebellar ataxia 38: structure-function analysis shows ELOVL5 G230V is proteotoxic, conformationally altered and a mutational hotspot

Ferrero et al.

```
Croaker.NP_0012 NGSPVSTNGHANGTPSLE-HAA-HKKLRVD
Rockfish.XP_037 NGSPISTNGHANGTPSME-RTA-YQKLRMD
AmSole.BBL33563 NGSPASRNGHANGKPSAE-QPA-HKKLRVD
Eel.XP_03525699 NGSAAAMNGHSNGVSPAE-DLT-RRKLRVD
Sturgeon.XP_033 NGSAASLNGHANGVSSVD-NVT-QRKLRKD
Zebrafish.NP_95 NGS---VNGHTNGVMSSE-KIK-HRKARAD
Eshark.XP_00789 NGS---ANGHTNGFSAQTDHYS-AKKTH--
WhaleShark.XP_0 NGSTIAANGHTNGLSTQMDHYS-EKKTH--
Macaca.XP_02870 PAGKEVKNGFSKAYFSAANGVM-NKKAQ--
Human.sp|Q9NXB9 PAGKEVKNGFSKAYFTAANGVM-NKKAQ--
XP_036301996.1  PAGKEVKNGFSKAYFTAANGVL-NKKAQ--
Bos.XP_02738008 -AGKEVKNGFSKAYFSTANGVI-SKKAQ--
Bwhale.XP_03672 HAGKEV-NGFSKAYFTATNGIT-NKKAQ--
Rat.XP_03274049 AAGKEVKNGFPKAHSIAANGVM-DKKVQ--
Mouse.NP_062296 ---KEVKNGFPKAHLIVANGMT-DKKAQ--
Gallus.NP_00118 PVTTEVKNGYHKNYFAASNGFLSNKKAQ--
Duck.XP_0273073 PVTTEVKNGFHKDYFTAANGFL-NKKAQ--
Frog.NP_0010161 PALTEMRNGYHKDLINASNGIQ-HKKEK--
Shark.XP_007900 ADVKEIKNGYYNGYITASNGNT-SKKKD--
ETE65396.1      PLVTEIKNGFHKDYLKEINGAL-KKKAQ--
Salmo.NP_001130 -----IKSSRPNGHSVSTNGTS-FKKRK--
Cherry          -----IKSSRPNGHSVSTNGTS-FKKRK--
Zebrafish.XP_00 AKVGYPKHSHSNGVSSSLNGAN-SKQKL-Q
                        .               ::
```

Spinocerebellar ataxia 38: structure-function analysis shows ELOVL5 G230V is proteotoxic, conformationally altered and a mutational hotspot

Ferrero et al.

Supplementary Table 8 ELOVL4 protein sequences for Multiple Sequence Alignment

	**Accession no.**	**Species**	**Length (AA)**
1	Q9GZR5.1	*Homo sapiens* (Human)	314
2	Q9EQC4.2	*Mus musculus* (Mouse)	312
3	XP_032765767.1	*Rattus norvegicus* (Rat)	314
4	AAI42460.1	*Bos taurus* (Bovine)	314
5	XP_046770079.1	*Gallus gallus* (Chicken)	314
6	XP_002936253.1	*Xenopus tropicalis* (Frog)	328
7	ADJ95235.1	*Salmo salar* (Salmon)	306
8	XP_001366145.1	*Monodelphis domestica* (Opossum)	314
9	XP_028902935.1	*Ornithorhynchus anatinus* (Platypus)	321
10	XP_007104948.1	*Physeter catodon* (Sperm whale)	314
11	XP_002714591.1	*Oryctolagus cuniculus* (Rabbit)	314
12	XP_008683076.1	*Ursus maritimus* (Polar bear)	314
13	XP_005299664.1	*Chrysemys picta bellii* (Painted turtle)	314
14	XP_011363206.1	*Pteropus vampyrus* (Large flying fox)	313
15	XP_026520959.1	*Notechis scutatus* (Tiger snake)	313

Spinocerebellar ataxia 38: structure-function analysis shows ELOVL5 G230V is proteotoxic, conformationally altered and a mutational hotspot

Ferrero et al.

Supplementary Table 9 MSA ELOVL5 (30 sequences) and ELOVL4 (15 Sequences)

```
CLUSTAL format alignment by MAFFT (v7.505) Method: L-INS-I, Blosum 62 matrix
HumanElovl5      ---------------------MEHFDASLSTYFKALLGPRDTRVKGWFLLDNYIPTFICS
Macaca.NP_00130 ---------------------MEHFDASLSTYFKALLGPRDTRVKGWFLLDNYIPTFICS
SeaLion.XP_0274 ---------------------MENFDASLSTYFKALLGPRDTRVKGWFLLDNYIPTFICS
Bat.XP_03626954 ---------------------MEHFDASLSTYFKALLGPRDTRVKGWFLLDNYIPTFICS
FruitBat.XP_037 ---------------------MENFDASLSTYFKAWLGPRDTRVKGWFLLDNYIPTFICS
Mouse.NP_599016 ---------------------MEHFDASLSTYFKAFLGPRDTRVKGWFLLDNYIPTFVCS
Rat.NP_599209.1 ---------------------MEHFDASLSTYFRALLGPRDTRVKGWFLLDNYIPTFVCS
Beaver.JAV42152 ---------------------MENFDASLSTYFRAWLGPRDTRVKGWFLLDNYIPTFVCS
Pangolin.XP_017 ---------------------MENFDASLSTYFKALLGPRDTRVKGWFLLDNYIPTFICS
ChPangolin.XP_0 ---------------------MENFDASLSTYFKALLGPRDTRVKGWFLMDNYIPTFICS
Mole.XP_0373757 ---------------------MENFDASLSTYFKALLGPRDTRVKGWFLLDNYIPTFICS
Bos.NP_00104006 ---------------------MEHFDASLSTYFRAWLGPRDTRVEGWFLLDNYVPTLVCS
BlueWhale.XP_03 ---------------------MEHFDASLSTYFQALLGPRDLRVKGWFLLDNYIPTLLCS
Dolphin.XP_0198 ---------------------MEHFDASLSTYFQALLGPRDPRVKGWFLLDSYIPTLLCS
Gallus.NP_00118 ---------------------MERLDKTINSYLDVWLGPRDPRVKGWLLLENYTPTFIFS
Duck.NP_0012973 ---------------------MERLDKTINSYLDVWLGPRDPRVKGWLLLENYTPTFIFS
Falcon.XP_03724 ---------------------MEFLDKTINSYLDVWLGPRDPRVKGWLLLENYTPTFIFS
Turtle.XP_03775 ---------------------MESLDQTINSYLDVWFGPRDPRVKGWLLLDNYIPTFIFS
Komodo.KAF72468 ---------------------MESLDKTINSYLDVWLGPRDPRVKGWLLLTNYTPTFIFS
Xenopus.NP_0010 ---------------------MEVLDKAVNGYIDHLLGPKDPRVRGWLLLDNYVPTILFT
Salmo.NP_001130 ---------------------MEAFNHKLNTYIDSWMGPRDERVQGWLLLDNYPPTFALT
Pike.AFR36912.1 ---------------------METFNQRLNTFIDSWMGPRDERVRGWLLLDNYPPTFALT
Croaker.NP_0012 ---------------------METFNHKLNTYLESWMGPRDQRVRGWLLLDNYPPTFALT
Rockfish.XP_037 ---------------------METFNHRLNTYLESWMGPRDQRVRGWLLLDNYPPTFALT
AmSole.BBL33563 ---------------------METFNHKLNAYIDSWLGPRDQRVRGWLLLDNYPPTFALT
Eel.XP_03525699 MD-------------------MEMFNHRLNTYIDSWMGPRDQRVRGWLLLDDYPPTFALT
Sturgeon.XP_033 ---------------------MEALNQATNAYIDSWMGPRDTRVRGWFLLDNFSPTVVLT
Zebrafish.NP_95 ---------------------METFSHRVNSYIDSWMGPRDLRVTGWFLLDDYIPTFIFT
Eshark.XP_00789 ---------------------MGFADQTLNSYFDSWFGPRDPRVEGFLLLDNYPPTFLLT
WhaleShark.XP_0 ---------------------MEAVDRTLNYYFVSWFGPRDSRVEGFFLLDKYPPTFILS
sp|Q9GZR5.1|ELO ------------------------------------------------------------
XP_008683076.1  MGL------LDSEPGSVLNVVSTALNDTVEFYRWTW-SITDKRVENWPLMQSPWPTLCIS
XP_002714591.1  MGL------LDSEPGSVLNVVSTALNDTVEFYRWTW-SIADKRVEDWPLMRSPWPTLSIS
sp|Q9EQC4.2|ELO ------------------------------------------------------------
XP_032765767.1  MGL------LDSEPGSVLNAVSTAFNDTVEFYRWTW-SIADKRVEDWPLMQSPWPTLSIS
XP_011363206.1  MGL------LDSEPGSVLNVVSTALNDTVEFYRWTW-SITDKRVENWPLMQSPLPTLCIS
AAI42460.1      MGL------LDSEPGSVLNAVSTALNDTVEFYRWTL-SITDKRVENWPLMQSPLPTLCIS
XP_007104948.1  MGL------LDSEPGSVLNVVSTALNDTVEFYRWTL-SITDKRVENWPMMQSPLPTLCIS
XP_001366145.1  MGL------LDAEPARMLDAVSTAVNDTIEFYRWTW-TIVDKRVENWPLMQSPLPTLCIS
XP_028902935.1  MEAPGAARPAALDALGALDAVSSALNGSLRFYRWTW-TIADKRVENWPLMQSPLPTLTIS
XP_046770079.1  MGA------AAAEPPRAAGLVSSVLNDTLEFYRWTW-SIRDKRVDDWPLMQSPFPTLTIS
XP_005299664.1  MGS------AAEQPGRATGLVSSLLNDTLEFYRWSW-SIRDKRVDDWFLMQSPFPTLTIS
XP_026520959.1  MGH-------SGEP----GLIANVVNDTLEFYRWSW-TIRDKRVDNWPMMQSPFPTLIIS
XP_002936253.1  ----------------MLSPVSDVIQRTLQLYDWCF-SIADKRVEKWPLMQSPLPTLAIS
ADJ95235.1      -----------------MEAVTHFMNDTVEFYRWSL-TIADKRVEKWPMMSSPAPTLAIS


HumanElovl5      VIYLLIVWLGPKYMRNKQPFSCRGILVVYNLGLTLLSLYMFCELVTGVWEGKYNFFCQGT
Macaca.NP_00130 VIYLLIVWLGPKYMRNKQPFSCRGILVVYNLGLTLLSLYMFCELVTGVWEGKYNFFCQGT
SeaLion.XP_0274 VIYLLIVWLGPKYMKNRQPFSCRGILVLYNLGLTLLSLYMFCELVKGVWEGRYNFFCQGT
Bat.XP_03626954 TIYLLIVWLGPKYMKNRQPLACRKILVVYNLGLTLLSLYMFCELVTGVWEGQYNFFCQGT
FruitBat.XP_037 VIYLLIVWLGPKYMKNRQPFSCRGILVVYNLGLTLLSLYMFCELVTGVWEGKYNFFCQGT
Mouse.NP_599016 VIYLLIVWLGPKYMKNRQPFSCRGILQLYNLGLTLLSLYMFYELVTGVWEGKYNFFCQGT
Rat.NP_599209.1 AIYLLIVWLGPKYMKNRQPFSCRGILVVYNLGLTLLSLYMFYELVTGVWEGKYNFFCQGT
Beaver.JAV42152 VIYLLIVWLGPKYMKNRQPFSCRGILVVYNLGLTLLSLYMFYELVTGVWEGKYNFFCQGT
Pangolin.XP_017 IIYLLIVWLGPKYMKNRQPFSCRGILVVYNLGLTLLSLYMFYELVTGVWEGRYNFFCQGT
ChPangolin.XP_0 IIYLLIVWLGPKYMKNRQPFSCRGILVVYNLGLTLLSLYMFYELVTGVWEGRYNFFCQGT
Mole.XP_0373757 VIYLLIVWLGPKYMKNRQAFSCRGILVVYNLGLTLLSLYMFCELVTGVWEGKYNFFCQDT
Bos.NP_00104006 ILYLLIVWLGPKYMKTRQPFSCRGILVVYNLGLTLLSLYMFCELVTGVWEGQYNFFCQGT
BlueWhale.XP_03 ILYLLIVWLGPKYMKNRPPFSCRGILVVYNLGLTLLSLYMFCELVTGVWEGQYNFFCQGT
Dolphin.XP_0198 ILYLLIVWLGPKYMKNRPPFSCRGILVLYNLGLTLLSLYMFCELVTGVWEGQYNFFCQGT
Gallus.NP_00118 VLYLLIVWLGPKYMRNKQPFSCRGILVVYNLGLTLLSLYMFYELVTGVWEGGYNFYCQDT
Duck.NP_0012973 VLYLLIVWLGPKYMRNKQPFSCRGILVVYNLGLTLLSLYMFYELVTGVWEGGYNFFCQDT
Falcon.XP_03724 VLYLLIVWLGPKYMRNKQPFSCRGILVVYNLGLTLLSLYMFYELVTGVWEGGYNFFCQDT
Turtle.XP_03775 VLYLLIVWLGPKYMQNKQPFSCRGILVVYNVGLTLLSLYMFYELVTGVWEGGYNFFCQDT
Komodo.KAF72468 VLYLLIVWLGPKYMQKRPPFSCRGILVVYNLGLTLLSLYMFYELVTGVWEGGYNFFCQDT
Xenopus.NP_0010 ALYLFIVWRGPKYMQNRPPVSCRGILVVYNLGLTLLSLYMFYELVTGVWEGGYNFFCQDT
Salmo.NP_001130 LMYLLIVWLGPKYMRHRQPVSCQGLLVLYNLALTLLSFYMFYEMVSAVWQGGYNFYCQDT
Pike.AFR36912.1 VIYLLIVWAGPKFMRHRQPLSCRGFLVVYNLGLALLSLYMFSEMVSAVWVGGYHFYCQDT
```

```
Croaker.NP_0012 VMYLVIVWMGPKYMKHRQPYSCRGLLVLYNLGLTLLSFYMFYELVTAVWHGGYNFYCQDI
Rockfish.XP_037 IMYLLIVWMGPKYMKHRQPYSCRGLLVLYNLGLTLLSFYMFYELVSAVRHGGYNFYCQDT
AmSole.BBL33563 VMYLLIVWLGPKYMKHRQPYSCRGLLVVYNLGLTLLSFYMFYELVGAVWHGDYNFYCQNT
Eel.XP_03525699 VAYLLIVWMGPKYMKNRQPFSCRGLLVVYNLGLTLLSLYMFCELVNGMWQGNYNFFCQDT
Sturgeon.XP_033 LLYLFVVWIGPKYMKNKEPFSCRGVLVVYNIGLTLLSFYMFYELVTGAWQGGYNFFCQDT
Zebrafish.NP_95 VMYLLIVWMGPKYMKNRQAYSCRALLVPYNLCLTLLSLYMFYELVMSVYQGGYNFFCQNT
Eshark.XP_00789 LIYLFVVWAGPKYMKNKAPVSCRGTLVVYNIGLTLLSLYMFYELLTGVWEGGYSIFCQDT
WhaleShark.XP_0 LLYLFVVWAGPKYMQNKAPLSSRGILVVYNIGLTLLSLYMFCELVRGVWDGGYSIFCQNT
sp|Q9GZR5.1|ELO -----------------EPFQMRLVLIIYNFGMVLLNLFIFRELFMGSYNAGYSYICQSV
XP_008683076.1  TLYLLFVWLGPKWMKDREPFQMRLVLIFYNFGMVLLNLFIFRELFMGSYNAGYSYICQSV
XP_002714591.1  TLYLLFVWLGPKWMKDREPFQMRLVLIIYNFGMVLLNLFIFRELLMGSYNAGYSYICQSV
sp|Q9EQC4.2|ELO -----------------EPFQMRLVLIIYNFGMVLLNLFIFRELFMGSYNAGYSYICQSV
XP_032765767.1  TLYLLFVWLGPKWMKDREPFQMRLVLIIYNFGMVLLNLFIFRELFMGSYNAGYSYICQSV
XP_011363206.1  TLYLLFVWLGPKWMKDREAFQMRLVLILYNFGMVLLNFFIFRELFMGSYNAGYSYVCQSV
AAI42460.1      TLYLLFVWLGPKWMKDREPFQMRLVLILYNFGMVLLNLFIFRELLMGSYNAGYSYICQTV
XP_007104948.1  TLYLLFVWLGPKWMKDREPFQMRLALIIYNFGMVLLNLFIFRELFMGSYNAGYSYICQSV
XP_001366145.1  TLYLLFVWLGPKWMKNREPFQMRLVLIVYNFGMVLLNMFIFRELFLASTAAGYSYICQSV
XP_028902935.1  TLYLLFVWLGPKWMKNKEPFQMRLVLILYNFGMVLLNLFIFKELFLGSYAAGYSYICQSV
XP_046770079.1  TIYLLTVWLGPKWMKTREPFQLRFLLVVYNFGMVLLNFFIFKELFLSSRARGYSYVCQTV
XP_005299664.1  TLYLLTVWLGPKWMRRREPFQLRFLLVSYNFGMVLLNFYIFKELFLASRARGYSYVCQTV
XP_026520959.1  TLYLLAVWLGPKWMKTREPFQFRFLLIFYNFGMVLLNFYIFKELLLSSRARGYSYICQSV
XP_002936253.1  TAYLLVVWLGPKFMKNREPFQLRYLLIAYNFGMVILNFFIFKELFLGAKAAGYSYICQPV
ADJ95235.1      CLYLLFLWAGPKYMQNREPFQLRKTLIVYNFSMVILNFYIAKELLLGARAAGYSYLCQPV
                                  .   :  *  **. :..:*.:::  *:. .     *   **

HumanElovl5     -RTAGESDMKIIRVLWWYYFSKLIEFMDTFFFILRKNNHQITVLHVYHHASMLNIWWFVM
Macaca.NP_00130 -RTAGESDMKIIRVLRWYYFSKLIEFMDTFFFILRKNNHQITVLHVYHHASMLNIWWFVM
SeaLion.XP_0274 -RSAGEADMKIIRVLWWYYFSKLIEFMDTFFFILRKNNHQITVLHVYHHASMLNIWWFVM
Bat.XP_03626954 -RSAGEADMKIIRVLWWYYFSKLIEFMDTFFFILRKNNHQITVLHVYHHASMLNIWWFVM
FruitBat.XP_037 -RSAGEADMKIIRVLWWYYFSKLIEFMDTFFFILRKNNHQVTVLHVYHHASMLNIWWFVM
Mouse.NP_599016 -RSAGESDMKIIRVLWWYYFSKLIEFMDTFFFILRKNNHQITVLHVYHHATMLNIWWFVM
Rat.NP_599209.1 -RSAGESDMKVIRVLWWYYFSKLIEFMDTFFFILRKNNHQITVLHVYHHATMLNIWWFVM
Beaver.JAV42152 -RSAGESDMKIIQVLWWYYFSKLIEFMDTFFFILRKNNHQITVLHVYHHASMLNIWWFVM
Pangolin.XP_017 -RSSGEADVKVIRVLWWYYFSKLIEFMDTFFFILRKNNHQITVLHVYHHVSMLNIWWFVM
ChPangolin.XP_0 -RSSGEADVKVIRVLWWYYFSKLIEFMDTFFFILRKNNHQITVLHVYHHVSMLNIWWFVM
Mole.XP_0373757 -RSAGEADMKIIHVLWWYYFSKLIEFMDTFFFILRKNNHQITVLHVYHHASMLNIWWFVM
Bos.NP_00104006 -RSGGEADMKIIRVLWWYYFSKLIEFMDTFFFILRKNNHQITVLHVYHHASMLNIWWFVM
BlueWhale.XP_03 -RSRGEADMKIVRVLWWYYFSKLIEFMDTFFFILRKNNHQVTVLHVYHHASMLNIWWFVM
Dolphin.XP_0198 -RSGGEADMKIIRVLWWYYFSKLIEFMDTFFFILRKNYHQVTVLHVYHHASMLNIWWFVM
Gallus.NP_00118 -HSGGEADMKIIRVLWWYYFSKLIEFMDTFFFILRKNNHQITVLHVYHHATMLNIWWFVM
Duck.NP_0012973 -HSGGEADMKIIRVLWWYYFSKLIEFMDTFFFILRKNNHQITVLHVYHHATMLNIWWFVM
Falcon.XP_03724 -HSGGEADMKIIRVLWWYYFSKLIEFMDTFFFILRKNNHQITVLHVYHHATMLNIWWFVM
Turtle.XP_03775 -HSGGEADMKIVRVLWWYYFSKLIEFMDTFFFILRKNHYQITVLHVYHHASMLNIWWFVM
Komodo.KAF72468 -HSGGEADMKIIRVLWWYYFSKLIEFMDTFFFILRKNNHQITVLHVYHHATMLNIWWFVM
Xenopus.NP_0010 -NSGGDADTKIVRVLWWYYFSKLIEFMDTFFFILRKNNHQITVLHVYHHASMLNIWWFVM
Salmo.NP_001130 -HSAGETDTKIINVLWWYYFSKVIEFMDTFFFILRKNNHQITFLHIYHHASMLNIWWFVM
Pike.AFR36912.1 -RSAGEADTRIIHVLWWYYFSKLIEFMDTFFFILRKNNHQITFLHIYHHASMLNIWWFVM
Croaker.NP_0012 -HSAQEVDNKIINVLWWYYFSKLIEFMDTFFFILRKNNHQITFLHIYHHASMLNIWWFVM
Rockfish.XP_037 -HSAQEVDNKIMNVLWWYYFSKLIEFMDTFFFIVRKNNHQITFLHIYHHASMLNIWWFVM
AmSole.BBL33563 -HSAPEVDKKVINVLWWYYFSKLIEFMDTFFFILRKNNHQITFLHIYHHASMLNIWWFVM
Eel.XP_03525699 -HSAGEADTKIINVLWWYYFSKLIEFMDTFFFILRKNNHQITFLHVYHHASMPSIWWFVM
Sturgeon.XP_033 -HSGGEADMKIVRVLWWYYFSKLIEFMDTFFFILRKNNHQITFLHVYHHATMPNIWWFVM
Zebrafish.NP_95 -HSGGDADNRMMNVLWWYYFSKLIEFMDTFFFILRKNNHQITFLHVYHHATMLNIWWFVM
Eshark.XP_00789 -HSAGKADLKIVRVLWWYYFSKVIEFMDTFFFILRKNYHQITILHVYHHATMFNIWWFVM
WhaleShark.XP_0 -HNAGEADLKIIRVLWWYYFSKVIEFMDTFFFILRKNYHQITVLHVYHHATMFNIWWFVM
sp|Q9GZR5.1|ELO DYSNNVHEVRIAAALWWYFVSKGVEYLDTVFFILRKKNNQVSFLHVYHHCTMFTLWWIGI
XP_008683076.1  DYSDNVNEVRIAAALWWYFVSKGVEYLDTVFFILRKKNNQVSFLHVYHHCTMFTLWWIGI
XP_002714591.1  DYSNNVNEVRIAAALWWYFVSKGVEYLDTVFFILRKKNNQVSFLHVYHHCTMFTLWWIGI
sp|Q9EQC4.2|ELO DYSNDVNEVRIAGALWWYFVSKGVEYLDTVFFILRKKNNQVSFLHVYHHCTMFTLWWIGI
XP_032765767.1  DYSNDVNEVRIAAALWWYFVSKGVEYLDTVFFILRKKNNQVSFLHVYHHCTMFTLWWIGI
XP_011363206.1  DYSENVHEVRIASALWWYFVSKGIEYLDTVFFILRKKNNQVSFLHVYHHCTMFTLWWIGI
AAI42460.1      DYSDNVHEVRIAAALWWYFISKGIEYLDTVFFILRKKNNQVSFLHVYHHCTMFTLWWIGI
XP_007104948.1  DYSDNVHEVRIAAALWWYFISKGIEYLDTVFFILRKKNNQVSFLHVYHHCTMFTLWWIGI
XP_001366145.1  NYSDDVNEVRIAGALWWYFVSKGVEYLDTVFFILRKKNNQVSFLHVYHHCTMFTLWWIGI
XP_028902935.1  DYSNNVHEVRIAAALWWYFVSKGIEYFDTVFFILRKKNNQVSFLHVYHHCTMFTLWWIGI
XP_046770079.1  DYSDNVYEVRIAAALWWYYVSKGIEYLDTVFFILRKKFNQISFLHVYHHFTMFTLWWIGI
XP_005299664.1  DYSDNIYEVRIAGALWWYFVSKGIEYLDTIFFILRKKFNQISFLHVYHHFTMFTLWWIGI
XP_026520959.1  DYSDNEYEVRIAGALWWYYISKGIEYLDTIFFILRKKFNQISFLHVYHHFTMFTLWWIGI
XP_002936253.1  DYSDDENEVRVASALWWYYVSKGVEYFDTVFFILRKKFNQISFLHVYHHCTMFTLWWIGI
ADJ95235.1      SYSNDVNEVRIASALWWYYISKGVEYLDTVFFILRKKINQVSFLHVYHHCTMFILWWIGI
                  .    : ::  .* **:.** :*::**.***:**:  *::.**:*** :*  :**: :

HumanElovl5     NWVPCGHSYFGATLNSFIHVLMYSYYGLSSV-PSMRPYLWWKKYITQGQLLQFVLTIIQT
```

Spinocerebellar ataxia 38: structure-function analysis shows ELOVL5 G230V is proteotoxic, conformationally altered and a mutational hotspot

Ferrero et al.

```
Macaca.NP_00130 NWVPCGHSYFGATLNSFIHVLMYSYYGLSSV-PSMRPYLWWKKYITQGQLLQFVLTIIQT
SeaLion.XP_0274 NWVPCGHSYFGATLNSFIHVLMYSYYGLSSV-PSVRPYLWWKKYITQGQLLQFVLTIIQT
Bat.XP_03626954 NWVPCGHSYFGATLNSFIHVLMYSYYGLSSV-PSMRPYLWWKKYITQGQLIQFVLTIIQT
FruitBat.XP_037 NWVPCGHSYFGATLNSFIHVLMYSYYGLSSV-PSMRPYLWWKKYITQGQLLQFVLTIVQT
Mouse.NP_599016 NWVPCGHSYFGATLNSFIHVLMYSYYGLSSI-PSMRPYLWWKKYITQGQLVQFVLTIIQT
Rat.NP_599209.1 NWVPCGHSYFGATLNSFIHVLMYSYYGLSSV-PSMRPYLWWKKYITQGQLVQFVLTIIQT
Beaver.JAV42152 NWVPCGHSYFGATLNSFIHVLMYSYYGLSSV-PSMRPYLWWKKYITQGQLIQFVLTIIQT
Pangolin.XP_017 NWVPCGHSYFGATLNSFIHVLMYSYYGLSSV-PSMRPYLWWKKYITQGQLLQFVLTIIQT
ChPangolin.XP_0 NWVPCGHSYFGATLNSFIHVLMYSYYGLSSV-PSMRPYLWWKKYITQGQLLQFVLTIIQT
Mole.XP_0373757 NWVPCGHSYFGATLNSFIHVLMYSYYALSSV-PSMRPYLWWKKYITQGQLLQFVLTIIQT
Bos.NP_00104006 NWVPCGHSYFGATLNSFIHVLMYSYYGLSSI-PSMRPYLWWKKYITQGQLLQFVLTIIQT
BlueWhale.XP_03 NWVPCGHSYFGATLNSFIHVLMYSYYGLSSV-PSMRPYLWWKKYITQGQLLQFVLTIIQT
Dolphin.XP_0198 NWVPCGHSYFGATLNSFIHVLMYSYYGLSSV-PSMRPYLWWKKYITQGQLVQFVLTILQT
Gallus.NP_00118 NWVPCGHSYFGATLNSFIHVLMYSYYGLSAV-PAMRPYLWWKKYITQGQLIQFVLTIFQT
Duck.NP_0012973 NWVPCGHSYFGATLNSFIHVLMYSYYGLSAV-PAMRPYLWWKKYITQGQLTQFVLTIFQT
Falcon.XP_03724 NWVPCGHSYFGATLNSFIHVLMYSYYGLSAV-PAMRPYLWWKKYITQGQLIQFVLTIFQT
Turtle.XP_03775 NWVPCGHSYFGATLNSFIHVLMYSYYGLSAI-PAMRPYLWWKKYITQGQLIQFVLTIFQT
Komodo.KAF72468 NWVPCGHSYFGATLNSFIHVLMYSYYGLSAI-PSMRPYLWWKKYITQGQLIQFVLTIFQT
Xenopus.NP_0010 NWVPCGHSYFGATLNSFIHVLMYSYYGLSAI-PAMRPYLWWKKYITQCQLTQFVLTMTQT
Salmo.NP_001130 NWVPCGHSYFGASLNSFVHVLMYSYYGLSAV-PAIRPYLWWKKYITQGQLIQFFLTMSQT
Pike.AFR36912.1 NWVPCGHCYFGACLNSFIHVLMYSYYGLSAI-PTMRPYLWWKKYITQGQLIQFFLTMTQT
Croaker.NP_0012 NWVPCGHSYFGASLNSFVHVVMYSYYGLSAI-PAMRPYLWWKKYITQLQLTQFFLTMSQT
Rockfish.XP_037 NWVPCGHSYFGASLNSFVHVVMYSYYGLSAI-PAIRPYLWWKKYITQFQLIQFFLTMFQT
AmSole.BBL33563 NWIPCGHSFFGASLNSFVHVVMYSYYGLSAI-PAMRPYLWWKRYITQLQLIQFFLTVSHT
Eel.XP_03525699 NWVPCGHSYFGASLNSFIHVLMYSYYGLSAV-PALRPYLWWKKYITQGQLIQFVMTMIQT
Sturgeon.XP_033 NWVPCGHSYFGASFNSFIHVLMYSYYGLSAI-PAVRPYLWWKKYITQGQLIQFVLTMFQT
Zebrafish.NP_95 NWVPCGHSYFGATFNSFIHVLMYSYYGLSAV-PALRPYLWWKKYITQGQLVQFVLTMFQT
Eshark.XP_00789 NWVPCGHSFFGATLNSFIHVLMYSYYGLSAI-PAMRPYLWWKKYITQGQLLQFVMTIIQT
WhaleShark.XP_0 NWVPCGHSFFGATLNSFIHVLMYSYYGLSAI-PAMRPYLWWKKYITQGQLLQFVLTIIQT
sp|Q9GZR5.1|ELO KWVAGGQAFFGAQLNSFIHVIMYSYYGLTAFGPWIQKYLWWKRYLTMLQLIQFHVTIGHT
XP_008683076.1  KWVAGGQAFFGAQLNSFIHVIMYSYYGLTAFGPWIQKYLWWKRYLTMLQLIQFHVTIGHT
XP_002714591.1  KWVAGGQAFFGAQMNSFIHVIMYSYYGLTAFGPWIQKYLWWKRYLTMLQLVQFHVTIGHT
sp|Q9EQC4.2|ELO KWVAGGQAFFGAQMNSFIHVIMYSYYGLTAFGPWIQKYLWWKRYLTMLQLVQFHVTIGHT
XP_032765767.1  KWVAGGQAFFGAQMNSFIHVIMYSYYGLTAFGPWIQKYLWWKRYLTMLQLVQFHVTIGHT
XP_011363206.1  KWVAGGQAFFGAQLNSFIHVIMYSYYGLTAFGPWIQKYLWWKRYLTMLQLVQFFVTIGHT
AAI42460.1      KWVAGGQAFFGAQINSFIHVIMYSYYGLAAFGPWIQKYLWWKRYLTMLQLVQFHVTIGHT
XP_007104948.1  KWVAGGQAFFGAQMNSFIHVIMYSYYGLTAFGPWIQKYLWWKRYLTMLQLVQFHVTIGHT
XP_001366145.1  KWVAGGQAFFGAQLNSFIHVIMYSYYGLTAFGPWIQKYLWWKRYLTILQLVQFYVTIGHT
XP_028902935.1  KWVAGGQAFFGAQLNSFIHVIMYSYYALAALGPQIQKYLWWKRYLTMLQLVQFHVTIGHT
XP_046770079.1  KWVAGGQAFFGAQMNAFIHVIMYMYYGLAACGPKVQKYLWWKRYLTILQLVQFHVTIGHT
XP_005299664.1  KWVPGGQAFFGAQMNSFIHVVMYMYYGLAACGPKFQKYLWWKRYLTILQLIQFHVTLGHT
XP_026520959.1  KWVAGGQAFFGAHINALIHVIMYMYYGLAACGPKFHKYLWWKRYLTIMQLLQFHVTIGHT
XP_002936253.1  KWVAGGQSFFGAHMNALIHVVMYLYYGLAACGPHLQKYLWWKRYLTILQLVQFHVTIGHT
ADJ95235.1      KWVPGGQSFFGAGINSSIHVLMYGYYGLAAFGPKIQKFLWWKKYLTIIQMIQFHVTIGHA
                :*:. *:.:*** :*: :**:** **.*::  * .: :****:*:*  *: ** :*: ::

HumanElovl5     SCGVIWPCTFPLGWLYFQIGYMISLIALFTNFYIQTYNKKGAS-------RRKDHLKDHQ
Macaca.NP_00130 SCGVIWPCTFPLGWLYFQIGYMISLIALFTNFYIQTYNKKGAS-------RRKDHLKDHQ
SeaLion.XP_0274 SCGVLWPCTFPLGWLYFQIGYMISLITLFTNFYVQTYNKKGAS-------RRKEHLKDHQ
Bat.XP_03626954 TCGVIWPCSFPLGWLYFQIGYMISLITLFTNFYIQTYNKQGAS-------RRKEHLKDHQ
FruitBat.XP_037 SCGVVWPCSFPLGWLYFQIGYMISLIILFTNFYIQTYNKKGAS-------RRKERLRDHQ
Mouse.NP_599016 TCGVFWPCSFPLGWLFFQIGYMISLIALFTNFYIQTYNKKGAS-------RRKDHLKGHQ
Rat.NP_599209.1 SCGVIWPCSFPLGWLYFQIGYMISLIALFTNFYIQTYNKKGAS-------RRKEHLKGHQ
Beaver.JAV42152 SCGVLWPCSFPLGWLYFQIGYMISLIALFTNFYIQTYNKKGAS-------RRRDHLKDHL
Pangolin.XP_017 SCGVVWPCTFPLGWLYFQIGYMISLIVLFTNFYVQTYNKKGAS-------RRREYLKDHQ
ChPangolin.XP_0 SCGVVWPCAFPLGWLYFQIGYMISLIVLFTNFYVQTYNKKGAS-------RRREYLKDHQ
Mole.XP_0373757 TCGVIWRCPFPLGWLYFQIGYMISLITLFTNFYIQTYNKKGAS-------RRKEHLKEHQ
Bos.NP_00104006 SCGVIWPCTFPLGWLYFQIGYMISLITLFTNFYIQTYNKKGVS-------RRREHQKDHQ
BlueWhale.XP_03 SCGVIWPCAFPLGWLYFQIGYMISLIALFTNFYIQTYNKKGAS-------RRREHQKDHQ
Dolphin.XP_0198 SCGVIWPCAFPLGWLYFQIGYMISLIALFTNFYIQTYNKKGAS-------RRRE----HQ
Gallus.NP_00118 SCGVVWPCAFPQGWLYFQIFYMISLIILFTNFYIQTYNKKASS-------RRKE----YQ
Duck.NP_0012973 SCGVVWPCAFPQGWLYFQISYMISLIILFTNFYIQTYNKKASS-------RRKE----YQ
Falcon.XP_03724 SCGVVWPCAFPQGWLYFQISYMISLIILFTNFYIQTYNKKASS-------RRKE----YQ
Turtle.XP_03775 SCGVVWPCAFPMGWLYFQIFYMISLIILFTNFYIQTYKKKASS-------RRKE----YQ
Komodo.KAF72468 SCGVVWPCPFPLGWLYFQIGYMITLIILFTNFYIQTYNKKAAC-------RRKE----YQ
Xenopus.NP_0010 TCAMIWPCKFPMGWLYFQNCYMISLIILFGNFYIKTYNKKTSS-------RRKE----YQ
Salmo.NP_001130 ICAVIWPCGFPRGWLFFQIFYMASLIAFFSNFYIQTYKKHRVS-------QKEY----HQ
Pike.AFR36912.1 ICAVIWPCGFPKGWLYFQISYMVTLIALFSNFYIQTYKKRSVS-------QKKEC---HR
Croaker.NP_0012 LCAVVWPCGFPMGWLYFQISYMVTLIFLFSNFYVQTYKKHSVS-------LKKE----HQ
Rockfish.XP_037 MCAVVWPCGFPKGWLSFQISYMVTLIFLFSNFYIQAYKKHSGS-------LKKE----HQ
AmSole.BBL33563 MCAVIWPCGFPMGWLFFQISYMVTLIILFSNFYIQTYQKHSRS-------LQKD----HR
Eel.XP_03525699 SCAVVWPCGFPRGWLYFQISYMVTLIALFSNFYIQTYQKQGAF-------RRKE----HQ
Sturgeon.XP_033 TCAVFRPCGFPMGWLYFQICYLIVLIVLFSNFYIQTYKKRGVS-------RRKE----CQ
```

Spinocerebellar ataxia 38: structure-function analysis shows ELOVL5 G230V is proteotoxic, conformationally altered and a mutational hotspot

Ferrero et al.

```
Zebrafish.NP_95 SCAVVWPCGFPMGWLYFQISYMVTLILLFSNFYIQTYKKRSGS-------RKSD----YP
Eshark.XP_00789 SFGVVWPCGFPSGWLYFQIGYMISLIVLFTNFYIQTYQKKSAS-------RTKD----CL
WhaleShark.XP_0 SFGVIWPCGFPSGWLYFQIGYMISLIILFTNFYIQTYKKKSAF-------KSKD----CL
Human ELOVL4     ALSLYTDCPFPKWMHWALIAYAISFIFLFLNFYIRTYKEPKKP------KAGKT----AM
XP_008683076.1  ALSLYTDCPFPKWMHWALIAYAISFIFLFLNFYVRTYKEPKKA------KTGKT----AV
XP_002714591.1  ALSLYTDCPFPKWMHWALIAYAISFIFLFLNFYIRTYNEPKKP------KTGKT----AM
sp|Q9EQC4.2|ELO ALSLYTDCPFPKWMHWALIAYAISFIFLFLNFYTRTYNEPKQS------KTGKT----AT
XP_032765767.1  ALSLYTDCPFPKWMHWALIAYAISFIFLFLNFYTRTYNEPKKS------KTGKT----AT
XP_011363206.1  ALSLYTDCPFPKWMHWALIAYAISFIFLFLNFYVRTYNEPKKS------KTGKT----AV
AAI42460.1      ALSLYTDCPFPKWMHWALIVYAVSFIFLFLNFYVRTYKEPKKA------KPGKT----AT
XP_007104948.1  ALSLYTDCPFPKWMHWALIAYAVSFICLFLNFYVRTYKEPKKA------KPGKT----AA
XP_001366145.1  ALSLYTDCPFPKWMHWALIVYAISFIFLFLNFYYQTYNDPKTS------KNRTA----AA
XP_028902935.1  AMSLYTNCPFPRWMHWALIVYAISFIFLFLNFYYQTYNQPKRSA-----KTGKT----AV
XP_046770079.1  ALSIYIDCPFPKWMHWGVIFYAITFIFLFGNFYYRTYKLPKEP------VKNGK----IA
XP_005299664.1  AMSIYIDCPFPKWMHWGVMIYAITFIFLFGNFYYRTYKMPKEP------IKNGK----IL
XP_026520959.1  ALSIYIDCPFPKWMHWGVIFYAASFIVLFGNFYYRTYKMPKQPKELKQKEKNGK----IT
XP_002936253.1  ALSLYIDCPFPKWMHWALIVYAITFIILFVNFYYRTYNAPKAPA-----KSGKS----LI
ADJ95235.1      GHSLYTGCPFPAWMQWALIGYAVTFIILFGNFYYQTYRRTPRSA----HKVAKP----VT
                  .:   * **         *   :* :* *** ::*.

HumanElovl5     NGSMAAVNG------------------HTNS-FSPLENN-------VKPRKLRKD--
Macaca.NP_00130 NGSVAAVNG------------------HTNS-FSPLENN-------VKPRKLRKD--
SeaLion.XP_0274 NGSVAAVNG------------------HTNS-F-SLENN-------VKPRKQRKD--
Bat.XP_03626954 NGSLTAANG------------------HTNS-FSSLENN-------VKLRKQRKD--
FruitBat.XP_037 NGSVTAANG------------------HTNS-FSSLGNS-------TKPRKQRKD--
Mouse.NP_599016 NGSVAAVNG------------------HTNS-FPSLENS-------VKPRKQRKD--
Rat.NP_599209.1 NGSMTAVNG------------------HTNN-FASLENS-------VTSRKQRKD--
Beaver.JAV42152 NGSATVVNG------------------HTNT-FSSLENN-------VKPRKQRKD--
Pangolin.XP_017 NGSMAAMNG------------------HAGS-FSSLGSS-------AKPRKLRKD--
ChPangolin.XP_0 NGSMAAVNG------------------HAGS-FSSLGNS-------VKPRKLRKD--
Mole.XP_0373757 NGSVAAVNG------------------HTNS-FSTLENN-------VKLRKQRKD--
Bos.NP_00104006 NGSVAAVNG------------------HISS-FSSLENN-------VKPRKQRKD--
BlueWhale.XP_03 NGSVTAVNG------------------HTSS-FSSLENN-------VKPRKQRKD--
Dolphin.XP_0198 NGSVTAVNG------------------HTSS-FSSLEHS-------VKPRKQRKD--
Gallus.NP_00118 NGSTATVNG------------------YTNS-FSSLENN-------VKQRKQRKD--
Duck.NP_0012973 NGSAATVNG------------------YTNS-FSSLENN-------VKQRKQRKD--
Falcon.XP_03724 NGSTATVNG------------------YTNS-SSSLENN-------VKQRKQRKD--
Turtle.XP_03775 NGSTAAVNG------------------YTNS-FSSLENN-------VKQRKQRKD--
Komodo.KAF72468 NGSTAAVNG------------------YTNS-FSSLENN-------VKQRKQRKD--
Xenopus.NP_0010 NGSASAVNG------------------HTNS-FSSLEDN-------VKQRKQRQD--
Salmo.NP_001130 NGSVDSLNG------------------HANG-VTPTE-T-------ITHRKVRVD--
Pike.AFR36912.1 NGSASPLNG------------------HVSG-VTPIE-T-------ITHRKVRAD--
Croaker.NP_0012 NGSPVSTNG------------------HANG-TPSLE-H-------AAHKKLRVD--
Rockfish.XP_037 NGSPISTNG------------------HANG-TPSME-R-------TAYQKLRMD--
AmSole.BBL33563 NGSPASRNG------------------HANG-KPSAE-Q-------PAHKKLRVD--
Eel.XP_03525699 NGSAAAMNG------------------HSNG-VSPAE-D-------LTRRKLRVD--
Sturgeon.XP_033 NGSAASLNG------------------HANG-VSSVD-N-------VTQRKLRKD--
Zebrafish.NP_95 NGS---VNG------------------HTNG-VMSSE-K-------IKHRKARAD--
Eshark.XP_00789 NGS---ANG------------------HTNG-FSAQTDH-------YSAKKTH----
WhaleShark.XP_0 NGSTIAANG------------------HTNG-LSTQMDH-------YSEKKTH----
sp|Q9GZR5.1|ELO NGI--SANG----------------------VSKSEKQLMIENG-KKQKNGKAKGD
XP_008683076.1  NGI--SANG----------------------VSKSEKQLVIENG-KKQKNGKAKGE
XP_002714591.1  NGI--SANG----------------------VNKSDNQLVIENG-KKQKNGKAKGE
sp|Q9EQC4.2|ELO NGI--SSNG----------------------VNKSEKAL--ENG-KPQKNGKPKGE
XP_032765767.1  NGI--SANG----------------------VNKSEKQLVLENG-KPQKNGKPKGE
XP_011363206.1  NGI--SANG----------------------VNKSEK-LVMENG-KKQKNGKAKGE
AAI42460.1      NGI--SANG----------------------VNKSENHLVVENG-KKQKNGKAKGE
XP_007104948.1  NGI--SANG----------------------VNQSENHLVVENG-KKQKNGKAKRE
XP_001366145.1  NGI--SANG----------------------ISKSEKQLVMENG-KKKHNGKAKGE
XP_028902935.1  NGI--TTNG----------------------VSKSESQSVVENG-KKKKNGKAKGE
XP_046770079.1  NGA--VANG----------------------VSKPENNPVVENG-KKQKKGKAKGE
XP_005299664.1  NGA--VANG----------------------VSKLENNLVVENG-KKQKKEKAKGE
XP_026520959.1  NGV--IANG----------------------KSKPENGAVMENG-KVQKK--AKGE
XP_002936253.1  NGK-TSVNGKSSVNGKCQINGKLMNGAVNGAVSKQDNKVGQENG-RKRRKGRAKRE
ADJ95235.1      NGVSMATNG----------------------YNKLQD--VEENGLKQQKKGRAKRE
                **     **                                       ::
```

Spinocerebellar ataxia 38: structure-function analysis shows ELOVL5 G230V is proteotoxic, conformationally altered and a mutational hotspot

Ferrero et al.

Supplementary Table 10 MSA of ELOVL5 (7 sequences) + ELOVL2 (7 sequences) + ELOVL4 (7 sequences)

```
CLUSTAL format alignment by MAFFT (v7.505); L-INS-I method; Blosum 62 matrix.
human_ELOVL5    --------------MEHFDASLSTYFKALLGPRDTRVKGWFLLDNYIPTFICSVIYLLIV
mus_ELOV5       --------------MEHFDASLSTYFKAFLGPRDTRVKGWFLLDNYIPTFVCSVIYLLIV
rat_ELOVL5      --------------MEHFDASLSTYFRALLGPRDTRVKGWFLLDNYIPTFVCSAIYLLIV
bos_ELOVL5      --------------MEHFDASLSTYFRAWLGPRDTRVEGWFLLDNYVPTLVCSILYLLIV
gallus_ELOVL5   --------------MERLDKTINSYLDVWLGPRDPRVKGWLLLENYTPTFIFSVLYLLIV
frog_ELOVL5     --------------MEVLDKAVNGYIDHLLGPKDPRVKGWLLLDNYVPTIFFTALYLFIV
salmo_ELOVL5    --------------MEAFNHKLNTYIDSWMGPRDERVQGWLLLDNYPPTFALTLMYLLIV
human_ELOVL2    -----------MEHLKAFDDEINAFLDNMFGPRDSRVRGWFMLDSYLPTFFLTVMYLLSI
bos_ELOVL2      -----------MEHLKAFDDEVNAFLDNMFGPRDSRVRGWFMLDSYLPTFSLTVLYLLSI
mus_ELOVL2      -----------MEQLKAFDNEVNAFLDNMFGPRDSRVRGWFLLDSYLPTFILTITYLLSI
rat_ELOVL2      -----------MEQLKAFDNEVNAFLDNMFGPRDSRVRGWFLLDSYLPTFTLTIVYLLSI
gallus_ELOVL2   -----------MEHLKAFDQEVNAFVDYMFGPRDARVRGWLLLDSYLPTFFLTVAYLLCI
frog_ELOVL2     -----------MEHIEKLDQEVNAFVDYLFGPRDTRVRGWLMLDSYLPTLFLTLLYLLSI
salmo_ELOVL2    -----------MNHLQSLDERLNKLFYFLFEDRDSRVRGWLLLDSYLPTLSLTILYLLSV
human_ELOVL4    MGLLDSEPGSVLNVVSTALNDTVEFYRWTWSIADKRVENWPLMQSPWPTLSISTLYLLFV
mus_ELOVL4      MGLLDSEPGSVLNAMSTAFNDTVEFYRWTWTIADKRVADWPLMQSPWPTISISTLYLLFV
rat_ELOVL4      MGLLDSEPGSVLNAVSTAFNDTVEFYRWTWSIADKRVEDWPLMQSPWPTLSISTLYLLFV
bos_ELOVL4      MGLLDSEPGSVLNAVSTALNDTVEFYRWTLSITDKRVENWPLMQSPLPTLCISTLYLLFV
gallus_ELOVL4   MGAAAAEPPRAAGLVSSVLNDTLEFYRWTWSIRDKRVDDWPLMQSPFPTLTISTIYLLTV
frog_ELOVL4     ----------MLNPVSDVIQRTLRLYDWCLSISDKRVEKWPLMQSPLPTLAISTVYLLVV
salmo_ELOVL4    -----------MELTTHLINDTMNLYKWALTIADKRVEKWPLMDNPLPTLAISSSYLLFL
                                                 * **  * :::.  **:  :  **: :

human_ELOVL5    WLGPKYMRNKQPFSCRGILVVYNLGLTLLSLYMFCELVTGVWEGKYNFFCQGTR-TAGES
mus_ELOV5       WLGPKYMKNRQPFSCRGILQLYNLGLTLLSLYMFYELVTGVWEGKYNFFCQGTR-SAGES
rat_ELOVL5      WLGPKYMKNRQPFSCRGILVVYNLGLTLLSLYMFYELVTGVWEGKYNFFCQGTR-SAGES
bos_ELOVL5      WLGPKYMKTRQPFSCRGILVVYNLGLTLLSLYMFCELVTGVWEGQYNFFCQGTR-SGGEA
gallus_ELOVL5   WLGPKYMRNKQPFSCRGILVVYNLGLTLLSLYMFYELVTGVWEGGYNFYCQDTH-SGGEA
frog_ELOVL5     WRGPKYMQNRQPVSCRSILVVYNLGLTLLSFYMFYELVTGVWEGGYNFFCQDTH-SGGDA
salmo_ELOVL5    WLGPKYMRHRQPVSCQGLLVLYNLALTLLSFYMFYEMVSAVWQGGYNFYCQDTH-SAGET
human_ELOVL2    WLGNKYMKNRPALSLRGILTLYNLGITLLSAYMLAELILSTWEGGYNLQCQDLT-SAGEA
bos_ELOVL2      WLGNRCMRNRPALSLRGILTLYNLGITLLSAYMLAELILSSWEGGYNLQCQDLT-SAGEA
mus_ELOVL2      WLGNKYMKNRPALSLRGILTLYNLAITLLSAYMLVELILSSWEGGYNLQCQNLD-SAGEG
rat_ELOVL2      WLGNKYMKNRPALSLRGILTLYNLGITLLSAYMLVELVLSSWEGGYNLQCQNLD-SAGEG
gallus_ELOVL2   WLGNKFMKNRQPFSLKAHLIVYNLGITLLSLYMLIELILATWEGGYNLQCQNLH-SAGEA
frog_ELOVL2     WLGTKYMKDRPAFSLRGHLIVYNLGVTLLSFYMLIELILSTWQGAYNLQCQNLD-SAGEA
salmo_ELOVL2    YLGSKYMRNRPAYSLKGVLQVYNFSVTMLSLYMLVELVSATLSAGYRLQCQGLH-EAGEA
human_ELOVL4    WLGPKWMKDREPFQMRLVLIIYNFGMVLLNLFIFRELFMGSYNAGYSYICQSVDYSNNVH
mus_ELOVL4      WLGPKWMKDREPFQMRLVLIIYNFGMVLLNLFIFRELFMGSYNAGYSYICQSVDYSNDVN
rat_ELOVL4      WLGPKWMKDREPFQMRLVLIIYNFGMVLLNLFIFRELFMGSYNAGYSYICQSVDYSNDVN
bos_ELOVL4      WLGPKWMKDREPFQMRLVLILYNFGMVLLNLFIFRELLMGSYNAGYSYICQTVDYSDNVH
gallus_ELOVL4   WLGPKWMKTREPFQLRFLLVVYNFGMVLLNFFIFKELFLSSRARGYSYVCQTVDYSDNVY
frog_ELOVL4     WLGPVFMKNREPFRLRYLLIAYNFGMVILNFFIFKELFLGAKAAGYSYICQTVDYSDDEN
salmo_ELOVL4    WLGPKYMQNREPFQLQKTLIVYNFSMVIFNFFICKELFLAARAAGYSYICQSVDYSDDPN
                : *   *: : .   :  *  **:.:.::. ::  *:. .     *   **      .

human_ELOVL5    DMKIIRVLWWYYFSKLIEFMDTFFFILRKNNHQITVLHVYHHASMLNIWWFVMNWVPCGH
mus_ELOV5       DMKIIRVLWWYYFSKLIEFMDTFFFILRKNNHQITVLHVYHHATMLNIWWFVMNWVPCGH
rat_ELOVL5      DMKVIRVLWWYYFSKLIEFMDTFFFILRKNNHQITVLHVYHHATMLNIWWFVMNWVPCGH
bos_ELOVL5      DMKIIRVLWWYYFSKLIEFMDTFFFILRKNNHQITVLHVYHHASMLNIWWFVMNWVPCGH
gallus_ELOVL5   DMKIIRVLWWYYFSKLIEFMDTFFFILRKNNHQITVLHVYHHATMLNIWWFVMNWVPCGH
frog_ELOVL5     DTKIIRVLWWYYFSKLIEFMDTFFFILRKNNHQITVLHVYHHASMLNIWWFVMNWVPCGH
salmo_ELOVL5    DTKIINVLWWYYFSKVIEFMDTFFFILRKNNHQITFLHIYHHASMLNIWWFVMNWVPCGH
human_ELOVL2    DIRVAKVLWWYYFSKSVEFLDTIFFVLRKKTSQITFLHVYHHASMFNIWWCVLNWIPCGQ
bos_ELOVL2      DIRVARVLWWYYFSKLIEFLDTIFFVLRKKTSQVTFLHVYHHASMFNIWWCVLNWIPCGQ
mus_ELOVL2      DVRVAKVLWWYYFSKLVEFLDTIFFVLRKKTNQITFLHVYHHASMFNIWWCVLNWIPCGQ
rat_ELOVL2      DIRVAKVLWWYYFSKLVEFLDTIFFVLRKKTSQITFLHVYHHASMFNIWWCVLNWIPCGQ
gallus_ELOVL2   DIRVAKVLWWYYFSKVIEFADTIFFVLRKKSSQITFLHVYHHATMFNIWWCVLNWIPCGQ
frog_ELOVL2     DVRVAKVLWWYYFSKAIEFMDTIFFVLRKKNSQITFLHVYHHATMFNIWWCVLNWIPCGQ
salmo_ELOVL2    DLRVAKVLWWYYFSKVIEFLDTIFFVLRKKNSQITFLHVYHHASMFNIWWCVLNWIPCGQ
human_ELOVL4    EVRIAAALWWYFVSKGVEYLDTVFFILRKKNNQVSFLHVYHHCTMFTLWWIGIKWVAGGQ
mus_ELOVL4      EVRIAGALWWYFVSKGVEYLDTVFFILRKKNNQVSFLHVYHHCTMFTLWWIGIKWVAGGQ
```

Spinocerebellar ataxia 38: structure-function analysis shows ELOVL5 G230V is proteotoxic, conformationally altered and a mutational hotspot

Ferrero et al.

```
rat_ELOVL4      EVRIAAALWWYFVSKGVEYLDTVFFILRKKNNQVSFLHVYHHCTMFTLWWIGIKWVAGGQ
bos_ELOVL4      EVRIAAALWWYFISKGIEYLDTVFFILRKKNNQVSFLHVYHHCTMFTLWWIGIKWVAGGQ
gallus_ELOVL4   EVRIAAALWWYYVSKGIEYLDTVFFILRKKFNQISFLHVYHHFTMFTLWWIGIKWVAGGQ
frog_ELOVL4     EVRVASALWWYYVSKGVEYFDTVFFILRKKFNQISFLHVYHHCTMFTLWWIGIKWVAGGQ
salmo_ELOVL4    EVRIAAALWWYFISKGVEYLDTVFFILRKKFNQVSFLHVYHHCTMFTLWWIGIKWVAGGQ
                : ::  .****:.** :*: **.**:***:  *::.**:*** :*:.:**  ::*:. *:

human_ELOVL5    SYFGATLNSFIHVLMYSYYGLSSV-PSMRPYLWWKKYITQGQLLQFVLTIIQTSCGVIWP
mus_ELOV5       SYFGATLNSFIHVLMYSYYGLSSI-PSMRPYLWWKKYITQGQLVQFVLTIIQTTCGVFWP
rat_ELOVL5      SYFGATLNSFIHVLMYSYYGLSSV-PSMRPYLWWKKYITQGQLVQFVLTIIQTSCGVIWP
bos_ELOVL5      SYFGATLNSFIHVLMYSYYGLSSI-PSMRPYLWWKKYITQGQLLQFVLTIIQTSCGVIWP
gallus_ELOVL5   SYFGATLNSFIHVLMYSYYGLSAV-PAMRPYLWWKKYITQGQLIQFVLTIFQTSCGVVWP
frog_ELOVL5     SFFGATLNSFIHVLMYSYYGLSAI-PAIRPYLWWKKYITQCQLTQFVLTMTQTTCAMIWP
salmo_ELOVL5    SYFGASLNSFVHVLMYSYYGLSAV-PAIRPYLWWKKYITQGQLIQFFLTMSQTICAVIWP
human_ELOVL2    SFFGPTLNSFIHILMYSYYGLSVF-PSMHKYLWWKKYLTQAQLVQFVLTITHTMSAVVKP
bos_ELOVL2      SFFGPTLNSFIHILMYSYYGLSVF-PSMHKYLWWKKYLTQAQLVQFLLTITHTMSAVVRP
mus_ELOVL2      SFFGPTLNSFIHILMYSYYGLSVF-PSMHKYLWWKKYLTQAQLVQFVLTITHTLSAVVKP
rat_ELOVL2      SFFGPTLNSFIHILMYSYYGLSVF-PSMHRYLWWKKYLTQAQLVQFVLTITHTLSAVVKP
gallus_ELOVL2   SFFGPTLNSFIHVLMYSYYGLSVI-PSMRKYLWWKKYLTQAQLIQFLLTIVHTLSAAVKP
frog_ELOVL2     SFFGPTLNSFIHVLMYSYYGLSVI-PSMHKYLWWKKYLTQAQLVQFLLTITHTLSAAVKP
salmo_ELOVL2    SFFGPTLNSFIHVCMYSYYGLSTI-PSMQKYLWWKRYLTQAQLIQFILTITHTLSAIVVP
human_ELOVL4    AFFGAQLNSFIHVIMYSYYGLTAFGPWIQKYLWWKRYLTMLQLIQFHVTIGHTALSLYTD
mus_ELOVL4      AFFGAQMNSFIHVIMYSYYGLTAFGPWIQKYLWWKRYLTMLQLVQFHVTIGHTALSLYTD
rat_ELOVL4      AFFGAQMNSFIHVIMYSYYGLTAFGPWIQKYLWWKRYLTMLQLVQFHVTIGHTALSLYTD
bos_ELOVL4      AFFGAQINSFIHVIMYSYYGLAAFGPWIQKYLWWKRYLTMLQLVQFHVTIGHTALSLYTD
gallus_ELOVL4   AFFGAQMNAFIHVIMYMYYGLAACGPKVQKYLWWKRYLTILQLVQFHVTIGHTALSIYID
frog_ELOVL4     SFFGAHMNALIHVVMYLYYGLAACGPHLQKYLWWKRYLTILQLVQFHVTIGHTALSLYID
salmo_ELOVL4    SFFGAHMNAAIHVLMYLYYGLASFGPKIQKYLWWKKYLTVIQMVQFHVTIGHTALSLYMD
                ::**. :*: :*: ** ****:   * :: *****:*:*  *: ** :*: :*   .

human_ELOVL5    CTFPLGWLYFQIGYMISLIALFTNFYIQTY----NKKGASRRKDHLKDHQNGSMAAVNGH
mus_ELOV5       CSFPLGWLFFQIGYMISLIALFTNFYIQTY----NKKGASRRKDHLKGHQNGSVAAVNGH
rat_ELOVL5      CSFPLGWLYFQIGYMISLIALFTNFYIQTY----NKKGASRRKEHLKGHQNGSMTAVNGH
bos_ELOVL5      CTFPLGWLYFQIGYMISLITLFTNFYIQTY----NKKGVSRRREHQKDHQNGSVAAVNGH
gallus_ELOVL5   CAFPQGWLYFQIFYMISLIILFTNFYIQTY----NKKASSRRKE----YQNGSTATVNGY
frog_ELOVL5     CKFPMGWLYFQNSYMISLIILFTNFYLKTY----NKKTSSRRKE----YQNGSASAVNGY
salmo_ELOVL5    CGFPRGWLFFQIFYMASLIAFFSNFYIQTY----KKHRVSQKEY----HQNGSVDSLNGH
human_ELOVL2    CGFPFGCLIFQSSYMLTLVILFLNFYVQTY----RKKPMKKDMQ----EPPAGKEVKNGF
bos_ELOVL2      CGFPLGCLIFQSSYMMTLVILFLNFYIQTY----RKKPMKKAME----E--AGKEVKNGF
mus_ELOVL2      CGFPFGCLIFQSSYMMTLVILFLNFYIQTY----RKKPVKKELQ----E----KEVKNGF
rat_ELOVL2      CGFPFGCLIFQSSYMMTLVILFLNFYIQTY----RKKPMKKEMP----EGAAGKEVKNGF
gallus_ELOVL2   CGFPFGCLMFQSSYMATLVILFVNFYIKTY----QKAPSRTAAK----ETPVTTEVKNGY
frog_ELOVL2     CGFPFGCLMFQSSYMTTLVMLFVNFYLKAY----KKRPSKSDPN----GIPVLTEMKNGY
salmo_ELOVL2    CGFPVGCLLFQFSYMATLVILFVNFYVQTY----RKRRPEE-------------SIKSSR
human_ELOVL4    CPFPKWMHWALIAYAISFIFLFLNFYIRTY----KEPKKPKAGKT---AMNGISA--NGV
mus_ELOVL4      CPFPKWMHWALIAYAISFIFLFLNFYTRTY----NEPKQSKTGKT---ATNGISS--NGV
rat_ELOVL4      CPFPKWMHWALIAYAISFIFLFLNFYTRTY----NEPKKSKTGKT---ATNGISA--NGV
bos_ELOVL4      CPFPKWMHWALIVYAVSFIFLFLNFYVRTY----KEPKKAKPGKT---ATNGISA--NGV
gallus_ELOVL4   CPFPKWMHWGVIFYAITFIFLFGNFYYRTY----KLPKEPVKNGK---IANGAVA--NGV
frog_ELOVL4     CPFPKWMHWALIVYAITFIILFANFYYRTY----NAPKAATKSGKS--LTNGKTS-VNGK
salmo_ELOVL4    CEFPHWMHYALICYALTFIALFGNFYYQTYRRTPRQPREPKASKA---LSNGVS---NGL
                * **         *  ::: :* *** ::*    .                      ..

human_ELOVL5    TNSFSPL-------------------------------ENNVKPRKLRK--D
mus_ELOV5       TNSFPSL-------------------------------ENSVKPRKQRK--D
rat_ELOVL5      TNNFASL-------------------------------ENSVTSRKQRK--D
bos_ELOVL5      ISSFSSL-------------------------------ENNVKPRKQRK--D
gallus_ELOVL5   TNSFSSL-------------------------------ENNVKQRKQRK--D
frog_ELOVL5     TNSFSSL-------------------------------EDNVKQRKQRQ--N
salmo_ELOVL5    ANGVTPT-------------------------------E-TITHRKVRV--D
human_ELOVL2    SKAYFTA-------------------------------ANGVMNKKAQ----
bos_ELOVL2      SKAYFST-------------------------------ANGVISKKAQ----
mus_ELOVL2      PKAHLIV-------------------------------ANGMTDKKAQ----
rat_ELOVL2      PKAHSIA-------------------------------ANGVTDKKVQ----
gallus_ELOVL2   HKNYFAA-------------------------------SNGFLSNKKAQ---
```

Spinocerebellar ataxia 38: structure-function analysis shows ELOVL5 G230V is proteotoxic, conformationally altered and a mutational hotspot

Ferrero et al.

```
frog_ELOVL2      HKDLINA-------------------------------SNGVLHKKEQ----
salmo_ELOVL2     PNGHSVS-------------------------------TNGTSFKKRK----
human_ELOVL4     SKSEKQLMI-----------------------------ENGKKQKNGKAKGD
mus_ELOVL4       NKSEKAL-------------------------------ENGKPQKNGKPKGE
rat_ELOVL4       NKSEKQLVL-----------------------------ENGKPQKNGKPKGE
bos_ELOVL4       NKSENHLVV-----------------------------ENGKKQKNGKAKGE
gallus_ELOVL4    SKPENNPVV-----------------------------ENGKKQKKGKAKGE
frog_ELOVL4      SSMNGKSPVNGKSQMNGKSLMNGAVNGAVSKQDYKVGQENGRKRRKGRAKRE
salmo_ELOVL4     SKGLGNGAVVGGGKVEEKERSSGVDN------------GNGRRKRKGRAKRD
```
